# A unifying theory for cognitive abnormalities in functional neurological disorders, fibromyalgia and chronic fatigue syndrome: systematic review

**DOI:** 10.1136/jnnp-2017-317823

**Published:** 2018-05-07

**Authors:** Tiago Teodoro, Mark J Edwards, Jeremy D Isaacs

**Affiliations:** 1 Neurosciences Research Centre, Molecular and Clinical Sciences Research Institute, St George’s, University of London, London, UK; 2 Department of Neurology, St George’s University Hospitals NHS Foundation Trust, London, UK; 3 Instituto de Medicina Molecular Faculdade de Medicina, Universidade de Lisboa & Serviço de Neurologia Hospital de Santa Maria, Lisboa, Portugal

**Keywords:** functional neurological disorder, memory, cognition, chronic fatigue syndrome, pain

## Abstract

**Background:**

Functional cognitive disorder (FCD) describes cognitive dysfunction in the absence of an organic cause. It is increasingly prevalent in healthcare settings yet its key neuropsychological features have not been reported in large patient cohorts. We hypothesised that cognitive profiles in fibromyalgia (FM), chronic fatigue syndrome (CFS) and functional neurological disorders (FNDs) would provide a template for characterising FCD.

**Methods:**

We conducted a systematic review of studies with cognition-related outcomes in FM, CFS and FND.

**Results:**

We selected 52 studies on FM, 95 on CFS and 39 on FND. We found a general discordance between high rates of subjective cognitive symptoms, including forgetfulness, distractibility and word-finding difficulties, and inconsistent objective neuropsychological deficits. Objective deficits were reported, including poor selective and divided attention, slow information processing and vulnerability to distraction. In some studies, cognitive performance was inversely correlated with pain, exertion and fatigue. Performance validity testing demonstrated poor effort in only a minority of subjects, and patients with CFS showed a heightened perception of effort.

**Discussion:**

The cognitive profiles of FM, CFS and non-cognitive FND are similar to the proposed features of FCD, suggesting common mechanistic underpinnings. Similar findings have been reported in patients with mild traumatic brain injury and whiplash. We hypothesise that pain, fatigue and excessive interoceptive monitoring produce a decrease in externally directed attention. This increases susceptibility to distraction and slows information processing, interfering with cognitive function, in particular multitasking. Routine cognitive processes are experienced as unduly effortful. This may reflect a switch from an automatic to a less efficient controlled or explicit cognitive mode, a mechanism that has also been proposed for impaired motor control in FND. These experiences might then be overinterpreted due to memory perfectionism and heightened self-monitoring of cognitive performance.

## Introduction

Practitioners in several settings report an increase in patients presenting with non-dementia cognitive symptoms in recent years.^1–3^ Notably, a range of benign cognitive symptoms are experienced by between 5% and 32% of healthy young adults.^4^ The term functional cognitive disorder (FCD) (or functional memory disorder) has been proposed to describe the subjective experience of cognitive dysfunction in the absence of underlying brain pathology.^5–8^ In common with historical practice in other functional neurological disorders (FNDs), the approach of cognitive specialists towards FCD has largely been to ‘rule out’ organic causes, rather than making an affirmative diagnosis on the basis of distinctive clinical features.^9^

Typical symptoms of FCD include forgetting tasks while preparing to execute them, inability to retrieve overlearnt memories, disrupted flow of thoughts and conversations, prospective memory failures and absent-mindedness.^5 6 9^ These burdensome symptoms are believed to occur in the absence of relevant objective deficits on neuropsychological assessment, that is, a discrepancy between subjective experience and objective performance, which is a canonical feature of FNDs.^8 10^ However, neuropsychological data on large cohorts of patients with FCD have not been reported. FCD is not a trivial disorder, with many patients continuing to experience distressing cognitive symptoms for months to years.^8^

Theoretically, FCD is proposed to be underpinned by psychological factors such as memory perfectionism, overinterpretation of attentional lapses and heightened self-monitoring for cognitive errors.^5 6 11^ However, the empirical evidence supporting this model is modest.

FCD is considered part of the spectrum of FND (also known as conversion disorder).^6^ FNDs in general are characterised by both ‘internal’ inconsistency and incongruity with other neurological disorders (‘external' inconsistency).^12^ With respect to FCD, internal inconsistency can be demonstrated, for example, by patients being able to provide detailed accounts of their memory lapses, while distinctive phenomenological differences with organic cognitive disorders constitute external inconsistency.^13^

People with FND most commonly present with abnormalities of movement control (eg, weakness, tremor and dystonic posturing) or attacks that resemble epilepsy (dissociative seizures/non-epileptic attacks). Theories regarding the underlying neurobiology of FND suggest a central role for misdirected attention towards movement/sensation.^14^ Of note, people with FND presenting with movement disorder or dissociative seizures commonly report cognitive difficulties.^9^

There is ongoing debate regarding the relevance of the growing pathophysiological understanding of FND to people who have chronic pain or chronic fatigue, who are typically diagnosed with fibromyalgia (FM) or chronic fatigue syndrome (CFS). FM and CFS can be regarded as functional somatic syndromes, a group of conditions affecting different body systems, but which are argued to have overlapping clinical and aetiological characteristics.^15^ In our clinical practice, it is very common to see patients with FND and comorbid FM and/or CFS. Importantly, FM and CFS are also associated with subjective cognitive difficulties, sometimes referred to as ‘brain fog’.^6 16–18^

The relationship between archetypal FCD and cognitive symptoms in the context of FM, CFS and FND remains unclear. Furthermore, the extent to which cognitive difficulties in these conditions are separate or overlapping disorders has not previously been studied. However, the extensive literature on cognitive symptomatology in these conditions affords an opportunity to compare them and to consider whether the cognitive symptoms of FND, FM, and CFS provide a template for understanding the emerging concept of FCD.

We conducted a systematic review of cognitive findings in patients with FM, CFS and FND, in order to define their key neuropsychological characteristics. Our hypothesis was that the cognitive profiles associated with functional motor, fatigue and pain syndromes would be similar to each other and to that proposed for isolated FCD. This would support the existence of a shared causal mechanism contributing to cognitive symptoms in these conditions with important implications for diagnosis and treatment.

## Methods

We performed a systematic review of studies with cognition-related outcomes in FND, FM and CFS. The protocol was registered with the PROSPERO database (CRD42017072044). Outcomes included self-reported cognitive difficulties and deficits on objective neuropsychological assessment.

More specifically, we planned to analyse and compare evidence regarding: cognitive symptoms; objective neuropsychological abnormalities (memory, attention and executive functions, information processing, language and social cognition); consistency on repeat assessment; performance validity testing (PVT); and concordance between self-reported symptoms and objective cognitive deficits. These domains were defined based on our aims, previous knowledge and preliminary review of the literature, including relevant review articles.

We searched PubMed and hand-searched reference lists for articles published in English language. JDI, MJE and TT defined search strategy and selection criteria. TT searched PubMed, hand-searched relevant reference lists and selected studies for inclusion in the final qualitative analysis. Further details regarding study identification (including ‘search terms’) can be found in the online (supplementary PRISMA diagrams for FND, FM and CFS studies).

Study quality was assessed based on: use of a control group, inclusion of PVT and blinding of neuropsychological assessment. A summary of the methodology and main findings of each selected study for each condition can also be found in the online (supplementary summary tables from FND, FM and studies).

## Results: evidence for abnormal cognition in FM, CFS and FND

For our final qualitative analysis (table 1), we selected 39 studies on FND including functional movement disorders (FMDs) and non-epileptic attacks (NEAs), 52 studies on FM and 95 on CFS. Further details regarding study selection, including PRISMA flow diagrams, can be found in the online supplementary materials. Study characteristics were highly heterogeneous. In general, the evidence was scarcer for FND, including only one study assessing the neuropsychological profile of patients with FMD.^19^ We characterise each study in more detail in the supplementary materials (supplementary summary tables from FND, FM and studies). Here we present a summary of the main findings, organised by topic and condition (table 1).

**Table 1:** Summary of main findings per condition and domain

Parameter	Functional cognitive disorder***	Fibromyalgia	Chronic fatigue syndrome	Functional neurological disorder†
Cognitive symptoms	Memory Language (word finding)	Attention/concentration Memory Language (word finding)	Attention/concentration Memory Language (word finding) Reasoning	Attention/concentration Memory Language (word finding)
Objective neuropsychological deficits				
Memory		Vulnerability to distraction Explicit worse than implicit memory*	Impaired registration and consolidation* Working memory disruption by abnormal attention/information processing*	Working memory**
Attention/concentration		Selective/divided attention* Bias towards emotionally negative information**	Divided attention Executive function of attention** Bias towards threatening stimuli*	Attention** Attentional bias towards social threatening stimuli**
Executive functions		Cognitive inhibition**		
Information processing			Slow	Slow**
Language		Verbal fluency*	Verbal fluency**	
Social cognition		Alexithymia** Recognising others’ emotions*		Alexithymia* Affect expression and recognition**
Consistency on repeat assessment			Increased performance variability	
Factors related with neuropsychological deficits		Pain*	Fatigue**	Psychopathology**
Discrepancies between symptoms and objective deficits	Symptoms>objective deficits	Symptoms>objective deficits	Symptoms>objective deficits	Symptoms>objective deficits**
Performance validity/effort testing	A minority fail validity testing	Only a minority fail validity testing*	Rare failures on validity testing*	Overall, only a minority fail validity testing
Neurobiology of cognitive impairment	No structural damage	No structural damage* Dysfunction of a fronto-parieto-temporal network involved in attention, memory and executive functions, as well in emotion and pain processing	No structural damage* Dysfunction of the working memory network	No structural damage
Others	Memory perfectionism Overinterpretation of attentional lapses Heightened self-monitoring for cognitive errors Low memory self-efficacy		Heightened perception of effort	

*, some evidence but inconsistent between studies; **, some evidence but particularly inconsistent and/or scarce; ***Proposed features.

†Non-epileptic attacks and functional movement disorders.

### Cognitive symptoms

#### Fibromyalgia

Patients with FM reported cognitive difficulties including forgetfulness, distractibility, speech/language difficulties and disorganised thinking.^20–24^ The proportion of patients reporting cognitive symptoms ranged from around 50% to 90%.^20 23 24^ This prevalence was higher than in healthy controls^21^ or controls with rheumatic disorders.^22–24^ Contributing factors included pain, fatigue, depressed mood and poor sleep.^22^

#### Chronic fatigue syndrome

Patients with CFS often experienced cognitive symptoms, including poor memory, impaired attention and word-finding and reasoning difficulties.^25–39^ The proportion of affected patients ranged from around 70 up to 90%.^25–28^ Cognitive difficulties were more prevalent than in healthy subjects.^24 27^ Coexisting fatigue,^28 38 40^ depression,^26 28 38 40 41^ anxiety^26 31 38 41^ and pain^29^ contributed to but did not entirely account for the severity of cognitive symptoms.^26^

#### Functional neurological disorder

A relatively small number of studies have investigated cognitive symptoms in patients with FND (including FMD^19^ and NEA^42–45^), with only one^19^ enrolling healthy controls. Patients with FMD reported more cognitive complaints than healthy subjects (50% vs 9.1%).^19^ Patients who had NEA reported more prominent cognitive difficulties in memory,^42^ attention/concentration^42^ and language^42 43^ domains than controls with epilepsy. Cognitive symptoms were correlated with the presence of depressed mood^42 44^ and post-traumatic stress disorder (PTSD).^45^

### Objective neuropsychological abnormalities

#### Memory

##### Fibromyalgia

Selective memory impairment was observed in tests that included stimulus competition,^46^ did not allow information rehearsal,^47^ were vulnerable to distraction^48^ and/or required sustained effort.^49^ Grisart *et al* 50 reported an impairment of explicit memory contrasting with a preservation of automatic memory processes. A further study reported abnormalities in implicit memory.^51^

Abnormal working memory was correlated with pain^48 52 53^ and anxiety^48^ but not with depression.^48 52 53^ Episodic memory impairment was correlated with pain^51 52^ and depression,^49^ but other studies did not find a link with comorbidities.^50 54^

##### Chronic fatigue syndrome

Working memory was often reported to be abnormal only in more effortful tasks, and this pattern was attributed to poor attention and slow information processing.^34 55–58^

Memory registration and consolidation were predominantly impaired relative to memory retrieval,^31 59–61^ which might reflect disruption of information acquisition and encoding by distraction.^59^ However, poor retrieval has also been described.^62 63^

Predominant impairment of verbal relative to visual memory has been described in some studies.^28 64–66^ However, other studies observed that both verbal and visual domains were affected,^67–69^ while others failed to demonstrate significant impairment of any memory domain.^70–75^

##### Functional neurological disorder

When compared with healthy subjects, patients with FMD had an isolated deficit on one verbal memory test,^19^ and patients who had NEA had a selective impairment of spatial working memory, associated with poor attention.^76^ In comparison with normative data, patients who had NEA demonstrated memory abnormalities,^77 78^ including impairment of working memory associated with poor attention and processing speed.^79^ However, normal memory performance has also been reported.^80^ Patients who had NEA performed similarly,^80–85^ similarly/better^43 79^ or better^44 86 87^ than patients with epileptic seizures (ESs).

Memory impairment was correlated with longer disease duration^87^ and psychopathology,^87^ including PTSD (along with substance abuse and use of psychopharmacological medication),^45^ low mood^44^ and anxiety.^43^

In summary, the available evidence is heterogeneous but does not support a global impairment of memory in FM, CFS or FND. However, some specific abnormalities have been described, which might reflect the effects of increased vulnerability to distraction on memory performance.

### Attention and executive function

Attention, executive function and working memory are closely interlinked. Indeed, storage and processing of items in working memory depends on attentional mechanisms. Moreover, executive function includes the ability to manage sources of distraction.^17^

#### Fibromyalgia

Abnormal attention has been related to an increased vulnerability of memory and other cognitive processes to distraction.^46 48 52 88–90^ However, some studies argue against a generalised impairment of attention.^91–95^

Miró *et al* 88 observed a selective impairment of ‘alerting’ and ‘executive control’ but not of ‘orienting’ components of attention (Attentional Network Test-Interactions). Impaired cognitive inhibition has been related to an inability to filter distracting information, underpinning the vulnerability to distraction.^46 47 96^ However, a specific impairment in cognitive inhibition was not confirmed in two studies.^97 98^ Overall, the evidence for abnormal executive function is mixed.^91 93–95 99 100^

Pain might exhaust attentional resources, thereby increasing susceptibility to distraction. An association between performance on cognitive tests probing attention and pain^89^ and measures of central sensitisation^90^ has been described. However, another study did not confirm this relationship.^88^ Additionally, attentional bias towards emotionally negative information may capture additional processing resources.^101^

#### Chronic fatigue syndrome

Patients with CFS do not appear to show generalised abnormalities of attention. However, prominent impairment of divided attention has been reported.^102^ Abnormalities on the Paced Auditory Serial Addition Test (PASAT) and Paced Visual Serial Addition Test have been repeatedly observed in CFS.^31 57 62 63 69 75 103–106^ Abnormal performance on these tests may also reflect poor divided attention.^107^

Impaired divided attention might account for poor performance on more effortful attentional tasks.^34 108 109^ Patients with CFS might be particularly prone to distraction by irrelevant stimuli, as suggested by impairment on the Stroop interference task.^27 32 110^ However, this finding was not confirmed in all studies.^25 35 111 112^ Attentional bias to threat^113 114^ and towards emotionally negative information^115^ have been observed. However, others did not confirm a bias towards illness-related information.^116 117^

A selective impairment of executive function of attention has been described.^118^ Abnormal executive attention has been related to cause slowing of information processing^118^ and an attentional bias to threat.^113 114^ Other studies have observed abnormalities in executive functions.^60 66 71 75 119 120^ Finally, impairments of selective and sustained attention and cognitive inhibition have been reported in subjects with comorbid FM.^121 122^

#### Functional neurological disorder

In comparison with healthy subjects, patients who had NEA, but not those with FMD, have been reported to have poor attention.^19 76 81^ This was associated with abnormal working memory,^76^ while executive functions were generally normal.^76 81^ In comparison with normative data, patients who had NEA demonstrated abnormalities in attention (associated with poor processing speed and working memory).^42 77 79^ In comparison with patients with ES, patients who had NEA performed similarly on tests of attention^43 44 80 83 85^ and executive function,^80 82 87^ with just one study reporting worse performance on attention.^79^

Overall, evidence across FM, CFS and FND suggests an impairment of selective and/or divided attention.

### Information processing

#### Fibromyalgia

Evidence for impaired information processing as probed by PASAT is mixed, with some studies reporting abnormalities^46 48^ and others normal performance.^93 94^ On 10 measures of information processing speed, patients with FM only performed worse than controls with memory complaints in Stroop colour and word naming; this was proposed to reflect delayed lexical access.^93^ Some studies did not confirm slower information processing or psychomotor speed.^52 94 95^ Reduced processing speed in mental arithmetic has been correlated with pain and opiate medication but not with depression, anxiety, fatigue and sleep disturbance.^123^

#### Chronic fatigue syndrome

Impaired information processing, as measured using PASAT, has been repeatedly reported.^31 57 62 63 69 103–106^ Information processing is related to attention, as it is thought to depend on how fast the attentional system operates. Notably, there was an association between poor divided attention and an impairment of information processing in more challenging settings, where patients need to allocate attention resources to multiple tasks.^31 55^ However, several authors failed to confirm an impairment of information processing^36 39 41 71 119 124^ and/or psychomotor slowing.^25 39 64^ When present, poor information processing was independent of depression,^31 57 62 63 69 103 106^ and the contribution of fatigue was inconsistent.^31 106 109 125^

#### Functional neurological disorder

In comparison with healthy subjects, patients who had NEA, but not those with FMD, showed poor information processing.^19 81^ In comparison with normative data, patients who had NEA also showed slow processing speed, along with poor attention and working memory.^79^ Patients who had NEA performed similarly to patients with ES in tests where information processing plays a role.^43 79 81 83 85^ On tests of psychomotor speed, patients who had NEA performed less well than healthy subjects^81^ and similarly to^81 87^ or worse^82^ than patients with ES.

### Language and other cognitive domains

#### Fibromyalgia

Verbal fluency provides a measure of how quickly and efficiently stored knowledge about words can be accessed.^17^ Patients with FM showed abnormal verbal fluency (and/or ‘verbal knowledge’).^52 91 93 126^ Delayed lexical access has been proposed to contribute to difficulties finding words or understanding word meaning.^126^ It has been linked with psychomotor slowing^99^ and with coexisting depression.^49^ However, other studies did not support these associations.^52 92 94^

#### Chronic fatigue syndrome

Evidence regarding abnormal verbal fluency is conflicting, with some studies reporting impairment,^125 127 128^ others only selective abnormalities^34 129^ and others essentially normal performance.^38 39 64 73 102 110 130^ CFS twins performed similarly to non-fatigued twins and healthy subjects in tests of language and reasoning.^112^ Performance on the Boston Naming Test has been consistently normal,^60 127 128^ and several other studies report normal higher cognitive functions, including language^63^ and abstract reasoning,^103 130 131^ although this last finding is not uniform.^109^ Language impairments including poor verbal fluency have been related to abnormal executive functioning but not with coexisting psychiatric disorders or use of psychoactive medication.^128^

#### Functional neurological disorder

Patients with FMD and healthy subjects performed similarly on verbal fluency tests.^19^ In comparison with normative data, patients who had NEA showed either naming and/or fluency abnormalities,^42 78 79^ or normal performances on a composite language score including naming, verbal comprehension and vocabulary.^80^ In comparison with patients with ES, patients who had NEA showed similar^79 82 83^ or better^43 44 78 81 85 87^ performance on language assessment (including confrontational naming, verbal fluency, verbal comprehension and/or writing speed). Longer disease duration was correlated with poorer scores in a language composite including naming, fluency, reading and comprehension.^87^

### Social cognition

Social cognition refers to the ability to decipher information about the intentions and affective states of others. This capacity is crucial for the implementation of appropriate behaviour during social interactions. We did not find studies assessing social cognition in CFS.

#### Fibromyalgia

Patients with FM showed impaired recognition of others’ emotions, impaired representation of other people’s affective mental states and higher levels of alexithymia but normal empathy quotients.^91^ These deficits were independent of executive function and depression/anxiety. Poor emotional face recognition has been associated with pain and, to a lesser degree, with depression, anxiety and alexithymia.^132^ Abnormal social cognition has been related to interoceptive impairment,^133^ as well as with an interference of nociception with cognitive and emotional processing, due to the partial overlap of brain networks involved in pain processing and face recognition.

#### Functional neurological disorder

Alexithymia is characterised by an inability to identify emotions at a cognitive level and has been repeatedly demonstrated in FND,^76 134 135^ although not uniformly.^136^ Patients who had NEA showed worse affect expression and recognition,^43^ impaired ability to switch between emotion and non-emotion face categorisation,^137^ positive attentional bias towards socially threatening stimuli^138^ and an overall greater intensity of emotional experience and diminished positive emotional behaviour on exposure to pleasant, neutral and unpleasant pictures.^139^ As an exception, normal facial expression recognition has also been described.^135^

### Consistency on repeat assessment

#### Chronic fatigue syndrome

Patients with CFS showed an increased intraindividual variability on performance on repeated neuropsychological testing.^140^ Significant variability of self-reported mental fatigue was also observed on repeated assessment.^141^

These findings can be regarded as evidence for internal inconsistency, which is considered a cardinal feature of functional neurological symptoms in general.^6^

### Discrepancies between self-reported cognitive difficulties and objective assessment

#### Fibromyalgia

Although self-reported difficulties are prominent, evidence for objective cognitive impairment is far more contradictory, suggesting an overestimation of cognitive problems. Unfortunately, only a handful of studies directly compared symptoms with objective performance. In two studies, prominent memory symptoms were disproportionate to objective impairment,^48 94^ particularly after controlling for fatigue, pain and depression.^94^ However, another observed a correlation between memory difficulties and objective performance.^142^ Cognitive symptoms have been associated with objective abnormalities in attention, processing speed, memory and executive functions.^52 98^

#### Chronic fatigue syndrome

Prominent reported difficulties in memory,^25 38 124^ attention/concentration,^25 124^ information processing^124^ and general cognitive function^30 38 131 143^ contrast with little or no deficit on objective assessment.^25 30 38 124 131 143^ Notably, prominent symptoms have been associated with impairment in only a *minority* of patients.^28^ Others observed a restricted pattern of neuropsychological impairment, involving attention/concentration, information processing and/or memory domains in the context of multiple, multimodal cognitive symptoms.^26 63 73^ Finally, a correlation between subjective and objective deficits has also been described, involving memory, attention and processing speed.^27 31–34 55 108 111 144^

‘Metamemory’ corresponds to self-knowledge about one’s own memory capabilities. Impossibly high standards of personal performance might contribute to discrepancies between self-reported and objective abnormalities. This has been related to a tendency to underestimate actual performance relative to expectations of ‘normal’ performance.^35^ Contrary to this hypothesis, two studies observed normal estimations of memory abilities and corresponding objective performance.^30 60^

#### Functional neurological disorder

A discrepancy between prominent symptoms and relatively normal objective performance has been observed in a few studies.^19 43 44^ However, in one of these, this dissociation was observed in only one among several cognitive domains.^43^ Moreover, another study found discrepancies in patients who had NEA and in those with ES.^42^

### Performance validity testing (PVT)

PVT was rarely included in test batteries assessing cognition in patients with FM or CFS. This is important in determining the cause of any underperformance on neuropsychological assessment. However, the few studies including PVT did not demonstrate a generalised lack of effort.

#### Fibromyalgia

Only a minority of patients with FM demonstrated suboptimal effort in the few studies that included PVT. However, the majority of participants performed normally on validity testing.^94 145–148^

The percentage of patients with FM passing PVT ranged from 63% in Johnson-Greene *et al* 148 and Brooks *et al*,^147^ which included subjects receiving or seeking disability benefits or with possible secondary gains, to up to 100% in Iverson *et al*,^145^ where patients were not involved in disability benefits assessments. In Gervais *et al*,^146^ 65% patients with FM who were on or seeking benefits performed in the normal range on PVT, and this percentage increased to 96% among those not seeking or receiving benefits. Poor performance on PVT was correlated with prominent cognitive symptoms,^146^ Millon Clinical Multiaxial Inventory-III scores, including somatoform, depression and anxiety subscales^147^ and pain and poor sleep.^148^ In one study, patients with FM showed prominent memory complaints but normal objective performance after excluding participants who demonstrated poor effort and controlling for pain, fatigue and depression.^94^

#### Chronic fatigue syndrome

In Busichio *et al* 75 the Test of Memory Malingering did not reveal lack of effort in any patient with CFS. In Constant *et al*,^68^ only 2/25 patients with CFS scored under the consistency threshold on PVT. Notably, these studies reported objective impairments of attention, information processing, memory and motor speed.^68 75^ A couple of other studies reported lack of evidence of poor motivation or effort.^67 71 118 140 149^

A different but related observation is a heightened perception of effort or fatigue after repeated or more challenging cognitive testing, which has been correlated with worse performance on cognitive testing.^36 131 149 150^

#### Functional neurological disorder

Performance validity testing in studies of patients with FND has yielded heterogeneous results. Several studies reported worse performance than in controls,^19 81 151 152^ while others reported performance that was normal^79^ or similar to neurological disease control groups.^151^

The percentage of patients passing on PVT ranged from 48.8%^153^ to 100%,^79^ with most studies suggesting that only a minority proportion of patients with FND fail effort testing.^151 154 155^ Increased failure in comparison with controls was reported by Drane *et al* 153 but not confirmed by Dodrill^82^ and other authors.^19 155^ Additionally, subjects with FND rarely scored below chance on forced-choice tests,^81 152 154^ which suggests that malingering is uncommon, at least in those patients with FND who are recruited to observational studies. Finally, the observation of ‘isolated’ effort deficits, contrasting with normal scores in many other neuropsychological tests also requiring ‘effort’,^19^ raises the hypothesis that some tests of performance validity may be measuring a specific cognitive deficit rather than incomplete effort.

## Discussion

FND, FM and CFS appear to be associated with similar, prominent cognitive symptoms, including forgetfulness, distractibility and word-finding difficulties. In contrast, generalised neuropsychological deficits have not been demonstrated.

Nevertheless, our systematic analysis of the literature reveals several specific abnormalities in FM, CFS and, to a lesser extent, FND. Poor selective and divided attention and slow information processing are reported across these disorders (table 1). Memory symptoms might therefore reflect increased vulnerability to distraction, which interferes with memory registration.

Coexisting psychopathology is not robustly associated with cognitive difficulties.^26 31 48 52 53 57 62 63 69 103 106 123 128^ However, both subjective cognitive symptoms and objective cognitive deficits correlate with pain in FM and with mental or physical exertion and fatigue in CFS.

Overall, these conditions show a mismatch between prominent cognitive symptoms and, at best, only selective objective abnormalities. Importantly, the few studies including PVT reported poor effort in only a small proportion of patients, with the exception of patients with FM receiving or seeking disability benefits, where one-third failed PVT.

The cognitive profiles of FM, CFS and non-cognitive FND share several features with the recently proposed FCD (table 1), including a disproportion between self-reported memory and word-finding difficulties and objective neuropsychological deficits and a strong bias towards symptoms with a likely attentional basis.

Some key features proposed to underpin FCD, including memory perfectionism, overinterpretation of attentional lapses and heightened self-monitoring for cognitive errors, have not been widely investigated in these disorders. However, in the CFS literature, there is some evidence for unrealistic expectations of cognitive performance,^34^ while in FM, there are well-described alterations in interoceptive awareness.^132^ These findings suggest the possibility of common risk factors, but empirical evidence confirming the relevance of these findings remains sparse.

Interestingly, neuropsychological assessment in FCD has not shown deficits in straightforward tests of attention,^8 10^ suggesting that it is a lack of attentional reserve rather than inattention per se that underpins the subjective experience of cognitive impairment. To date, however, the more complex tests of attention used in the literature on FND, CFS and FM have not been reported in an FCD cohort.

**Figure 1:**
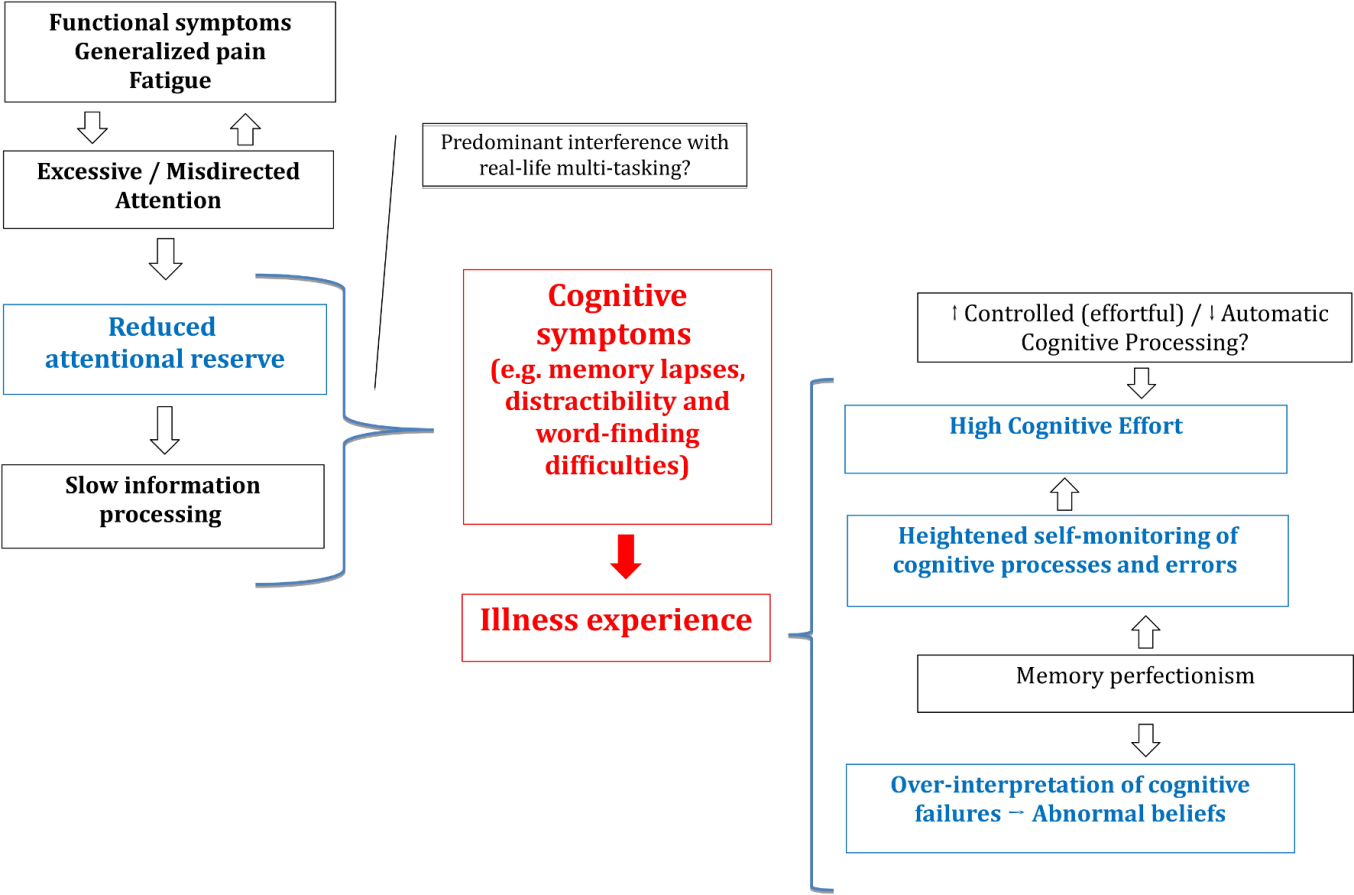
Proposed mechanisms underpinning cognitive difficulties across the spectrum of functional cognitive disorders spectrum.

Reduced attentional reserve could increase susceptibility to distraction and slow information processing. Heightened distractibility and attentional lapses could then contribute to the subjective experience of memory impairment (figure 1). Abnormal attention might have a disproportionate impact on real-life multitasking, but be more difficult to measure using standard neuropsychological evaluation.^102^

Excessive attention towards the body, severe pain and fatigue might underlie the decrease in externally directed attention. In the context of alexithymia, difficulties in recognising somatic markers of emotions might promote body monitoring and further exhaust attentional resources (figure 1).

Memory perfectionism and heightened self-monitoring of cognitive processes and errors might then lead to overinterpretation of such attentional lapses in daily life which, in turn, could drive advice-seeking from healthcare professionals (figure 1). Interestingly, the ability to ‘normalise’ somatic experiences inversely correlates with health seeking behaviour in FM.^156^ Perfectionism may be relevant in FM and CFS. Women with FM do not have greater perfectionist traits overall, but perfectionism correlates with poor health status.^157^ In CFS, self-critical perfectionism has been shown to predict fatigue and pain levels.^158^ However, the concept of memory perfectionism as a core feature of, or risk factor for, FCD needs further elucidation.

Another interesting observation is the heightened perception of effort after repeated or more challenging cognitive testing in patients with CFS.^36 131 149 150^ Heightened perception of effort is proposed to reflect an interpretive bias for somatic information,^116^ a greater operational demand to control and monitor cognitive processes^159^ or a sense of insufficiency resulting from unrealistically high expectations about one’s performance, leading to an overestimation of exertion and fatigue.^35^

If present across this group of disorders, a heightened perception of cognitive effort may also contribute to the experience of illness, due to the extreme effort invested in otherwise trivial daily tasks. The experience of illness would then be decoupled, to a degree, from objective cognitive performance. Therefore, abnormal effort may well constitute a piece in the jigsaw of FCD.

Cognitive effort can be regarded as a cost of *controlled* cognitive processes.^160^ A switch from an *automatic* to a *controlled* mode of cognitive processing may therefore underlie the increase in cognitive effort. Notably, this would mirror what is observed in FMD, where a switch from an automatic to an explicit (‘conscious’) mode of motor control impairs motor performance.^161^

In addition, the hypothetical switch from an automatic to a highly controlled (and effortful) cognitive operational mode could be a maladaptive attempt to compensate for ‘memory failures’ that were invested with pathological significance. Paradoxically, an exaggerated investment of effort could also contribute to the failure on performance validity testing by a minority of patients. Indeed, motivation depends on a cost–benefit analysis,^162^ and a ‘high effort’ operational mode could lead to earlier disengagement due to unacceptably high performance costs.

Although our systematic review focused on FND, FM and CFS, there are other conditions where prominent cognitive difficulties contrast with inconsistent neuropsychological deficits and an absence of structural lesions on conventional neuroimaging.

Postconcussion syndrome (PCS) is characterised by a constellation of ‘non-specific’ neuropsychiatric symptoms following a concussion or mild traumatic brain injury. Similarly to FND, FM and CFS, patients report poor concentration, mental slowness, forgetfulness and word-finding difficulties.^163^

Neuropsychological assessment in PCS does not confirm widespread cognitive deficits.^164^ However, specific abnormalities in selective and divided attention, information processing and working memory have been described, again mirroring observations in FND, FM and CFS.^165–168^ This similarity was corroborated by direct comparisons between PCS and CFS.^61^

Whiplash syndrome follows a hyperflexion, hyperextension or hyperlateroversion of the neck without external head injury or loss of consciousness. Patients typically describe chronic pain involving neck/shoulders. Cognitive difficulties and other associated symptoms are similar to PCS.^169–171^ Notwithstanding the controversy regarding the inconsistency of objective neuropsychological deficits, a meta-analysis reported objective abnormalities in attention, working memory, immediate and delayed recall, visuomotor tracking and cognitive flexibility.^172^

Functional imaging studies in the disorders investigated here, as well as in PCS, have described dysfunction of a fronto-parieto-temporal network postulated to be involved in attention, memory and executive functions as well as in emotion and pain processing.^36 173–179^ This would provide a neurobiological basis for the proposed interaction between attention-related cognitive functions and abnormalities with pain and emotional processing as well as movement control.

Interestingly, negative expectations regarding the consequences of a head injury and recall bias have both been implicated in self-reported symptoms after concussion.^180 181^ This gains particular relevance given the proposed role of abnormal predictions or expectations in the pathogenesis of functional neurological symptoms.^14^

### Limitations

Evidence for the presence and characteristics of cognitive abnormalities in FNDs, FM and CFS is remarkably heterogeneous.

Sample sizes were usually small, and a control group of healthy subjects was often lacking. In Vercoulen *et al*,^28^ objective cognitive impairment in a minority of patients with CFS was sufficient to drive between-group differences towards statistical significance. In these disorders, population heterogeneity means that small sample sizes and the use of average scores (rather than, eg, the proportion of individuals performing below threshold) might conceivably lead to false-positive results.

Notably, cognition in FND has been far less studied than in FM and CFS. Moreover, among 39 studies on FND, 36 focused on NEA, 1 in neurological ‘medically unexplained symptoms’^154^ and only 2 on FMD.^19 134^ Although we decided to use the more encompassing term ‘FND’, available evidence does not allow us to conclude much about the cognitive profile of FMD. However, it would be reasonable to expect a degree of overlap among FND subtypes.

Patients with FM, CFS and FND are often treated with medications such as benzodiazepines or opioids, which can have cognitive side effects. Unfortunately, the possible contribution of medication to cognitive performance was infrequently assessed. Although blinding in neuropsychological evaluations was rare, the increased availability of computerised evaluations might have helped reduce observer bias.

Despite the ongoing discussion about the discordance between subjective and objective abnormalities, relatively few studies investigated discrepancies within the same sample. In addition, PVT was rarely included in neuropsychological assessment, despite the pervasive belief that patients ‘exaggerate’ their symptoms, do not apply sufficient effort in cognitive testing or may be seeking secondary gains.

The variety of methods used for PVT was striking. Furthermore, the definition of ‘poor effort’ was heterogeneous, including: scoring below chance in forced-choice tests, scoring below/above a prespecified cut-off proposed to distinguish ‘poor effort’ and scoring lower than controls in PVT. It is important to highlight that poor effort does not necessarily equate to malingering or a factitious disorder. For example, patients with FM are frequently affected by performance anxiety, and one can imagine that it may interfere with falsely ‘challenging’ effort tasks. Another contributor could be ‘cogniphobia’, a concept akin to ‘kinesiophobia’ that reflects a reluctance to engage in any cognitive task, due to a fear that doing so may aggravate other symptoms.^182^

Finally, we acknowledge that there is ongoing debate about the aetiology and diagnosis of CFS and FM, in particular the relevance or not of ‘organic’ and ‘non-organic’ factors.^183 184^ There is considerable debate, particularly with regard to CFS, about how to define and diagnose the condition, and indeed whether there may be a number of different conditions present within groups of patients diagnosed with CFS. We accept that these debates are important and as yet unresolved and that heterogeneity in the CFS and FM populations reviewed here might have reduced the power of studies to identify consistent deficits.

## Conclusion

Given the considerable heterogeneity in the literature and its methodological shortcomings, further research is warranted before reaching firm conclusions about the nature of cognitive difficulties in FND, CFS and FMD or the existence of an overarching ‘functional cognitive disorder spectrum’, also encompassing conditions such as PCS.

However, the available evidence does not support the existence of separate cognitive disorders in CFS, FM and FND. Rather, these conditions share common cognitive symptoms with an emphasis on attentional dysfunction. The newly emerging concept of FCD shares similar symptoms and hypothesised mechanistic underpinnings. Our understanding of these cognitive disorders and the development of novel interventions might be facilitated by regarding them as related conditions whose cognitive symptoms fall within the FCD spectrum, rather than as discrete entities. While evidence-based treatment for FCD remains lacking, interventions for fatigue, pain and functional non-cognitive neurological symptoms are available.^185–187^ Therefore, practitioners assessing patients with FCD should look for these features, which may be missed if the consultation is exclusively focused on cognitive complaints. Clinical trials in FCD might reasonably also include subjects with CFS, FM or FND-related cognitive difficulties, which will generate larger sample sizes and lead to faster development of evidence-based treatment for this increasingly prevalent and distressing disorder.

10.1136/jnnp-2017-317823.supp1 Supplementary file 1

10.1136/jnnp-2017-317823.supp2 Supplementary file 2

10.1136/jnnp-2017-317823.supp3 Supplementary file 3

10.1136/jnnp-2017-317823.supp4 Supplementary file 4

10.1136/jnnp-2017-317823.supp5 Supplementary file 5

10.1136/jnnp-2017-317823.supp6 Supplementary file 6
