# Supplementary material for: A unifying theory for cognitive abnormalities in functional neurological disorders, fibromyalgia and chronic fatigue syndrome: systematic review
Source: J Neurol Neurosurg Psychiatry. 2018 May 7;89(12):1308–19. doi: 10.1136/jnnp-2017-317823 (PMC6288708; doi:10.1136/jnnp-2017-317823)
Supplement: Supplementary file 2 [file jnnp-2017-317823supp002.pdf]

PRISMA 2009 Flow Diagram: Fibromyalgia

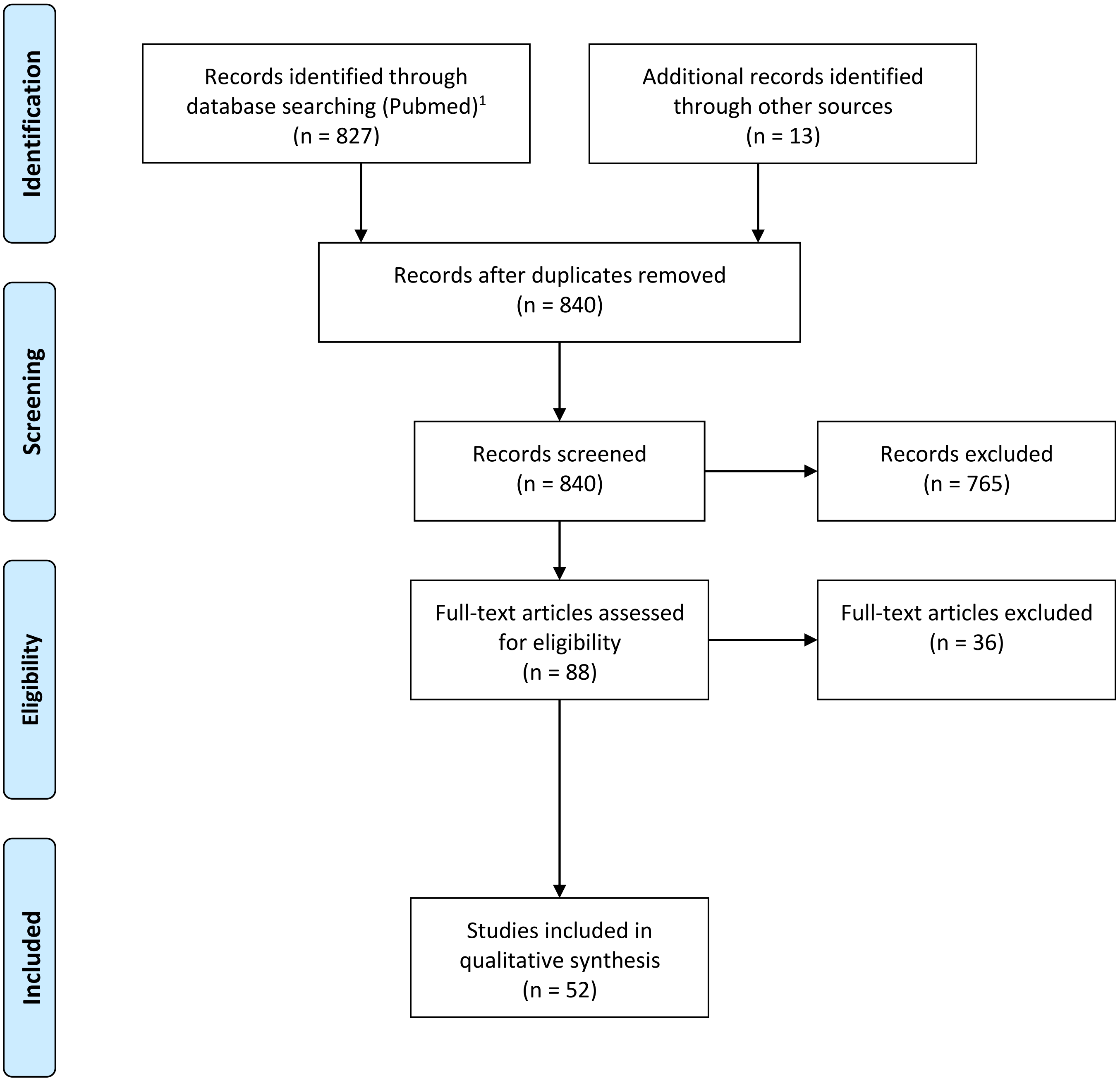

([1]) Details of database-search strategy:

- Database: Pubmed
- Search date: 22/12/2015
- Key words: ("fibromyalgia"[All fields] or "fibro" [All fields]) AND ("cognition"[All fields] OR "cognitive"[All fields] OR "memory"[All fields] OR "fog"[All fields])

From: Moher D, Liberati A, Tetzlaff J, Altman DG, The PRISMA Group (2009). *P*referred *R*eporting *I*tems for *S*ystematic Reviews and *M*eta-*A*nalyses: The PRISMA Statement. PLoS Med 6(7): e1000097. doi:10.1371/journal.pmed1000097

For more information, visit www.prisma-statement.org.
