# Supplementary material for: A unifying theory for cognitive abnormalities in functional neurological disorders, fibromyalgia and chronic fatigue syndrome: systematic review
Source: J Neurol Neurosurg Psychiatry. 2018 May 7;89(12):1308–19. doi: 10.1136/jnnp-2017-317823 (PMC6288708; doi:10.1136/jnnp-2017-317823)
Supplement: Supplementary file 3 [file jnnp-2017-317823supp003.pdf]

PRISMA 2009 Flow Diagram:
Functional Neurological Disorders

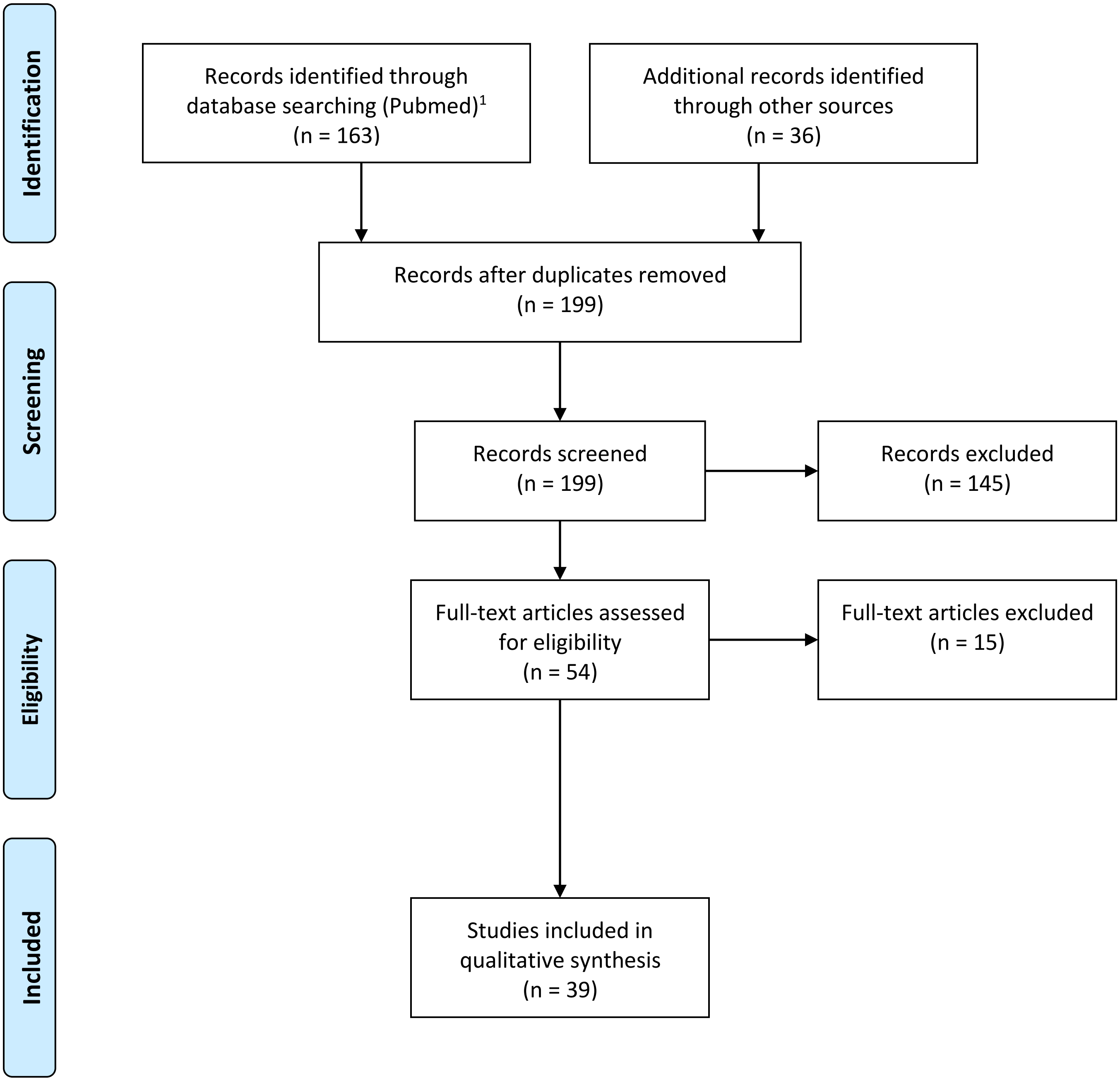

([1]) Details of database-search strategy:

- Database: Pubmed
- Search dates:
    - functional movement disorders (FMD): 22/12/2015
    - non-epileptic attacks (NEA): 30/01/2016

From: Moher D, Liberati A, Tetzlaff J, Altman DG, The PRISMA Group (2009). *P*referred *R*eporting *I*tems for *S*ystematic Reviews and *M*eta-*A*nalyses: The PRISMA Statement. PLoS Med 6(7): e1000097. doi:10.1371/journal.pmed1000097

- Key words:
    - For FMD: *(“psychogenic movement disorders”[All fields] OR “functional movement disorders”[All fields]) AND ("cognition"[All fields] OR "cognitive"[All fields] OR "memory"[All fields] OR "fog"[All fields])*
    - For NEA: *(“non-epileptic”[All fields] OR “nonepileptic”[All fields] OR “non epileptic” [All fields] OR “dissociative seizure”[All fields] OR“dissociative seizures”[All fields] OR “dissociative attack”[All fields] OR “dissociative attacks”[All fields] OR “conversion seizure”[All fields] OR “conversion seizures”[All fields] OR “conversion attack”[All fields] OR “conversion attacks”[All fields] OR “hysterical seizure”[All fields] OR “hysterical attack”[All fields] OR “pseudoseizure”[All fields] OR “pseudo seizure”[All fields] OR “pseudo-seizure”[All fields]) AND ("cognition"[All fields] OR "cognitive"[All fields] OR "memory"[All fields] OR "fog"[All fields])*
