# Supplementary material for: A unifying theory for cognitive abnormalities in functional neurological disorders, fibromyalgia and chronic fatigue syndrome: systematic review
Source: J Neurol Neurosurg Psychiatry. 2018 May 7;89(12):1308–19. doi: 10.1136/jnnp-2017-317823 (PMC6288708; doi:10.1136/jnnp-2017-317823)
Supplement: Supplementary file 4 [file jnnp-2017-317823supp004.pdf]

## CFS studies - quality and summary of main findings

| Study | Title | CFS group (n) | Control group (n) | Performance validity testing | Blinded assessment (B - blinded; U - unblinded; C - computerized) | Main objective | Neuropsychological tests | Main findings |
|---|---|---|---|---|---|---|---|---|
| *Altay 1990* | The neuropsychological dimensions of postinfectious neuromyasthenia (chronic fatigue syndrome): a preliminary report | CFS (21) | | | U | To investigate whether subjective cognitive complaints in postinfectious neuromyasthenia could be quantified objectively. | Attention and concentration: Trails A and B, The Digit symbol subtest of the Wechsler Adult Intelligence Scale-Revised (WAIS-R); abstract reasoning (general intellectual ability) - the Similarities subtest of the WAIS-R | CFS patients scored better than their age matched norms on tests of concentration, attention and abstraction. There was a discrepancy between subjective cognitive complaints and objective performances. |
| *Attree 2014* | Psychosocial factors involved in memory and cognitive failures in people with myalgic encephalomyelitis/chronic fatigue syndrome | CFS (87) | | | U | To explore the association between a number of different variables (self-efficacy, social support, anxiety, depression, and fatigue) and self-reported cognitive performance in CFS. | | Fatigue, depression, and general self-efficacy were directly associated with cognitive failures and retrospective (but not prospective) memory. |
| *Beaumont 2012* | Reduced Cardiac Vagal Modulation Impacts on Cognitive Performance in Chronic Fatigue Syndrome | CFS (30) | HC (40) | | C | The objective of this study was to examine the relationship between HRV and cognitive performance in patients with CFS. | Digit Symbol test, Spatial Working Memory task and Stroop-Colour-Word test. | Patients with CFS showed no deficits in performance accuracy, but were significantly slower than healthy controls. CFS was further characterized by low and unresponsive HRV; greater heart rate (HR) reactivity and prolonged HR-recovery after cognitive challenge. Fatigue levels, perceived effort and distress did not affect cognitive performance. HRV was consistently associated with performance indices and significantly predicted variance in cognitive outcomes. |

| | | | | | | | | |
|---|---|---|---|---|---|---|---|---|
| *Blackwood 1998* | Effects of exercise on cognitive and motor function in chronic fatigue syndrome and depression | CFS (10) | Depression (10); HC (10) | Subjective judgement | U | To examine the muscular, cognitive, symptomatic, and effort perception responses to physical exertion in patents with chronic fatigue syndrome. | Working memory / auditory attention (digit span, from WAIS-R); psychomotor speed (digit symbol, also from WAIS-R); word fluency (FAS test); selective attention and sustained attention (telephone search and lottery tasks from the test of everyday attention). | Patients with chronic fatigue syndrome show a specific sensitivity to the effects of exertion on effortful cognitive functioning. After exertion they showed a greater decrease on everyday tests of focused  and sustained attention than HC, as well as greater deterioration than depressed patients on the focused attention task. This occurs despite subjective and objective evidence of effort allocation. |
| *Brimacombe 2002* | Immunological Variables Mediate Cognitive Dysfunction in Gulf War Veterans but Not Civilians with Chronic Fatigue Syndrome | CFS (68) (40 Gulf war veterans and 28 civilians); healthy unmedicated control group (64) (33 Gulf war veterans and 31 civilians) | | | U | To explore the relationship between immunological variables and cognitive and functional status measures and a diagnosis of chronic fatigue syndrome (CFS) in civilians and veterans. | NP battery included 18 cognitive measures designed to assess attention, concentration, information processing, executive functions, learning and memory and visual-spatial functions, as well as motor speed: simple and complex reaction time tests; the Paced Auditory Serial Addition Test; the Wechsler Adult Intelligence Scale-Revised Digit Span subtest; Trail Making Test A and B; the computerized version of the Category Test; the Verbal Fluency Test; the California Verbal Learning Test; the Continuous Visual Memory Test; the Judgment of Line Orientation Test; the Rey-Osterrieth Complex Figure copy component, and the Grooved Pegboard Test. | We found that one particular cognitive factor – namely speed and accuracy of doing a reaction time task – predicted CFS caseness independently of any of the other variables studied. |

| | | | | | | | | |
|---|---|---|---|---|---|---|---|---|
| *Busichio 2004* | Neuropsychological deficits in patients with chronic fatigue syndrome | CFS (141) | HC (76) | TOMM | U | To help determine the degree of neuropsychological dysfunction across domains in CFS. | Memory - CVLT , Rey-Osterreith Complex Figure Test, Digit Span forward subtest of the WAIS–R; Attention/concentration - Digit Span subtest (total score), Digit Symbol subtest from the WAIS–R; Speed of information processing - total PASAT score, CPT, Trails Making Test A; Motor speed - The Grooved Pegboard task and the Simple Reaction Time task; Executive functioning - The Digit Span backwards subtest of the WAIS–R, the Trails Making Test B, Rey-Osterreith Complex Figure—copy and the Category Test. | Overall, it appears that CFS patients are having most difficulty concentrating and processing information when required to respond motorically. The findings in the present investigation further suggest that CFS patients have difficulty when completing a complex information-processing task. CFS group was also more likely than the healthy cohort to fail at least one test (defined as scoring below the 5th percentile of the healthy mean) in the domains of attention, processing speed, and motor speed, but not in the domains of memory and executive processing. |
| *Capuron 2006* | Cognitive Dysfunction Relates to Subjective Report of Mental Fatigue in Patients with Chronic Fatigue Syndrome | CFS (43) | nonfatigued subjects (53) age-, sex-, and race/ethnicity-matched | | C | The ascertain if mental fatigue contributes for the heterogeneity in findings regarding cognitive impairment in CFS patients. | NP assessment consisted in CANTAB battery encompassing: psychomotor coordination and motor speed - reaction time (RTI); Reasoning and planning abilities - The Stockings of Cambridge (SOC) task; Memory - The spatial working memory, The pattern recognition memory test, The spatial recognition memory test; Attention - The attentional shift intra/extra dimensional shift task, The rapid visual information processing test | In CFS patients with significant mental fatigue, sustained attention performance was impaired only in the final stages of the test, indicating greater cognitive fatigability in these patients. CFS patients with low mental fatigue displayed performance comparable to nonfatigued subjects on all tests of the CANTAB battery. These findings show strong concordance between subjective complaints of mental fatigue and objective measurement of cognitive impairment in CFS patients and suggest that mental fatigue is an important component of CFS-related cognitive dysfunction. |

| | | | | | | | | |
|---|---|---|---|---|---|---|---|---|
| *Caseras 2006* | Probing the Working Memory System in Chronic Fatigue Syndrome: A Functional Magnetic Resonance Imaging Study Using the n-Back Task | CFS (17) | HC (12) | | C | To examine the neural correlates of working memory in patients with CFS. | n-Back Task | Both groups performed comparably well and activated the verbal working memory network during all task levels. However, during the 1-back condition, patients with CFS showed greater activation than control subjects in medial prefrontal regions, including the anterior cingulate gyrus. Conversely, on the more challenging conditions, patients with CFS demonstrated reduced activation in dorsolateral prefrontal and parietal cortices. Furthermore, on the 2- and 3-back conditions, patients but not control subjects significantly activated a large cluster in the right inferior/medial temporal cortex. Trend analyses of task load demonstrated statistically significant differences in brain activation between the two groups as the demands of the task increased. |
| *Christodoulou 1998* | Relation between neuropsychological impairment and functional disability in patients with chronic fatigue syndrome | CFS (53) | HC who did not exercise regularly (32) | | U | To examine the relation between neuropsychological impairment and functional disability in patients with chronic fatigue syndrome, and determine whether the relation is independent of psychiatric factors. | Verbal memory - California verbal learning test (CVLT); Visual memory - Rey-Osterreith complex figure (ROCF); Attention and concentration - paced auditory serial addition test (PASAT) and F and B digit span from WAIS-R | A relation was found between cognitive impairment and functional disability which could not be explained entirely on the basis of psychiatric factors. CFS patients with a higher numbers of failing neuropsychological test scores reported significantly more days of general inactivity in the past month. This result remained significant even after partialling out the contribution of the presence of a comorbid axis I psychiatric episode and the overall level of depressive symptomology. Patients with failing verbal memory scores were particularly functionally disabled compared with those with passing scores. |

| | | | | | | | | |
|---|---|---|---|---|---|---|---|---|
| *Claypole 2001* | Cognitive Compromise Following Exercise in Monozygotic Twins Discordant for Chronic Fatigue Syndrome: Fact or Artifact? | monozygotic twin with CFS (22) | monozygotic twin without CFS (22) | | U | To examine the effects of exhaustive exercise on cognitive functioning among twin pairs discordant for chronic fatigue syndrome (CFS). | The Wechsler Adult Intelligence Scale–Revised Digit Span Forward and Backward subtests were administered to examine brief auditory attention span and the more effortful concentration and working memory. The Hopkins Verbal Learning Test consisting of three trials of free recall of a 12-item semantically categorized word list evaluated verbal learning and memory. The Digit Vigilance Test evaluated visual motor speed of information processing. Finally, word fluency was assessed with the Controlled Oral Word Association Test and category fluency with animal naming. | The preexercise neuropsychological test performance of CFS twins tended to be slightly below that of the healthy twin controls on all measures. However, twins with CFS did not demonstrate differential decrements in neuropsychological functioning after exercise relative to their healthy co-twins. |
| *Claypole 2007* | A Twin Study of Cognitive Function in Chronic Fatigue Syndrome: The Effects of Sudden Illness Onset | monozygotic twin with CFS (22) | monozygotic twin without CFS (22) | | B | To investigate neuropsychological deficits in CFS while controlling for confounders including genetic and environmental influences and the effects of comorbid depression and mode of illness onset. | Wechsler Adult Intelligence Scale; The motor functioning domain - Halstead–Reitan Finger Tapping Test, Grooved Pegboard Test, Simple Reaction Time Test; Speed of information processing domain - parts of Stroop Color and Word Test and Paced Auditory Serial Addition Task; Verbal memory domain - Rey Auditory Verbal Learning Test, Verbal Memory Index of the Wechsler Memory Scale—Revised, which includes Logical Memory Subtests I and II and Verbal Paired Associates I and II, Visual memory domain - Rey–Osterrieth Complex Figure Test, Benton Visual Retention Test; Executive functioning | Neuropsychological performance of fatigued twins was worse than that of their healthy co-twins in four of the six cognitive domains evaluated, including speed of information processing, verbal memory, and motor and executive functioning. Neither lifetime nor current major depression affected cognitive function in the twins with CFS. Sudden onset CFS was associated with a significant decrement in speed of information processing. |

| | | | | | | | | |
|---|---|---|---|---|---|---|---|---|
| | | | | | | | domain - Trail Making Test Part B ,  Stroop Color and Word Test, Controlled Oral Word Attention Test, Wisconsin Card Sorting Test | |

| | | | | | | | | |
|---|---|---|---|---|---|---|---|---|
| *Constant 2011* | Cognitive deficits in patients with chronic fatigue syndrome compared to those with major depressive disorder and healthy controls | CFS (25) | MDD (25); HC (25) | Amsterdam Short Memory Test; indexes from Word Memory Test and Doors Test | C | To compare the cognitive performance of patients with CFS and patients with depression. | Attention - Phasic Alertness Task of the TEA battery; Working memory - working memory task of the TEA battery, paced auditory serial attention test; Episodic memory - word memory test (WMT); | Patients with CFS had slower phasic alertness, and also had impaired working, visual and verbal episodic memory compared to controls. They were, however, no more sensitive than the other groups to suggestibility or to fatigue induced during the cognitive session. Cognitive impairments in MDD patients were strongly associated with depression and subjective fatigue; in patients with CFS, there was a weaker correlation between cognition and depression (and no correlation with fatigue). In conclusion, the authors claimed that these findings confirms the presence of an objective impairment in attention and memory in patients with CFS but with good mobilization of effort and without exaggerated suggestibility. |
| *Cook 2005* | Exercise and Cognitive Performance in Chronic Fatigue Syndrome | CFS-only (20) | CFS FM (19); CON (26) | | C | To determine the effect of submaximal steady-state exercise on cognitive performance in patients with chronic fatigue syndrome (CFS) | Automated neuropsychological assessment matrices (ANAM), which is comprised of: simple reaction time (SRT), running memory (RM), memory recall (MR), math processing (MP), and matching to sample (MS) | CFS patients without comorbid FM exhibit subtle cognitive deficits in terms of speed of processing, performance variability and efficiency that are not improved or exacerbated by light exercise. |

| | | | | | | | | |
|---|---|---|---|---|---|---|---|---|
| | | | | | | alone, CFS with comorbid fibromyalgia FM (CFS FM), and sedentary healthy controls (CON). | | |
| *Cope 1995* | Cognitive Functioning and Magnetic Resonance Imaging in Chronic Fatigue | CFS (26) | HC (18); Psych C (depressed) (13) | | U | To examines whether cognitive dysfunction in chronic fatigue may be accounted for by depression and anxiety or is due to brain pathology evident on magnetic resonance imaging (MRI). | National Adult Reading Test; Wechsler Adult Intelligence Scale - Revised; Wechsler Memory Scale- Revised; Warrington Recognition Memory Test; sell-assessment of memory; Graded Naming Test; Word Stem Completion, a test of implicit memory; supraspan; verbal fluency; speed of information processing. | No substantial differences in performance were shown between subjects with chronic fatigue and controls. Subjective cognitive dysfunction increased with psychopathology. White-matter lesions were found in a minority from all groups. Improvement in fatigue and depression coincided with improved performance on cognitive measures. |
| *Creswell 2002* | Underlying self-esteem in chronic fatigue syndrome | CFS (24) | chronic ilness (24); HC (24) | | U | To investigate underlying self-esteem using information-processing measures and overtly administered measures of self-esteem. | Emotional Stroop Test using neutral, positive, and negative trait words were administered. | Participants with CFS reported lower self-esteem than the two comparison groups on overt measures. Overt responses, however, did not fully account for the full extent of the interference effect from the negative word Stroop compared to the positive word Stroop. |

| | | | | | | | | |
|---|---|---|---|---|---|---|---|---|
| *Daly 2001* | Neuropsychological Function in Patients With Chronic Fatigue Syndrome, Multiple Sclerosis, and Depression | CFS (29) | MS (24); major depressive disorder (23); healthy controls (25) | | C | To compare the neuropsychological performance of patients with CFS, MS, and depression. Psychiatric symptomatology, including depression and anxiety, were also evaluated in the same individuals to determine their relation to cognitive performance. | Attention - Digit Span Forward; language - Boston Naming Test and verbal fluency; memory - Russell version of the Wechsler Memory Scale and a word list learning test; set shifting and conceptualization (aspects of executive function) - Stroop Interference Test and Proverb Interpretation; spatial ability - figure copying from the WMS and cube copying from the Consortium to Establish a Registry for Alzheimer's Disease battery; overall IQ - vocabulary subtest of the Wechsler Adult Intelligence Scale. The computerized battery: attention - continuous performance task; associate learning test - verbal memory; pattern memory test - nonverbal memory; spatial ability - pattern matching and hand–eye coordination test; set shifting - task adaptation of a switching-attention test. | CFS group was impaired on all four of the memory tasks as well as in language tasks (category fluency for animals), suggesting difficulties with both language skills and planning and organizational abilities related to executive function. After covarying the cognitive test scores by a measure of depression, the patient groups continued to differ from controls primarily in the area of memory. The findings support the view that the cognitive deficits found in CFS cannot be attributed solely to the presence of depressive symptomatology in the patients. |
| *DeLuca 1993* | Information Processing Efficiency in Chronic Fatigue Syndrome and Multiple Sclerosis | CFS (12) | MS (11); HC (11) | | U | To compare the cognitive performance of subjects with chronic fatigue syndrome (CFS), multiple sclerosis (MS), and healthy controls. | Paced Auditory Serial Addition Test (PASAT) (information processing speed and eficiency); Digit Span Test forward and backwards (attention/concentration); Similarities Test (Verbal Abstract Reasoning); Vocabulary subtest of the WAIS-R (verbal intelligence). | These results indicate that subjects with CSF and subjects with MS show significant impairment on a test of complex concentration when compared with appropriate controls. No significant correlations were found between BDI scores and PASAT total scores, or between BDI and Digit Span Test. The data suggest that subjects with CFS and subjects with MS have difficulty on tasks that require the simultaneous processing of complex cognitive information. Selective impairment in information processing efficiency may lie at the root of other cognitive complaints made by |

| | | | | | | | | |
|---|---|---|---|---|---|---|---|---|
| | | | | | | | | patients with CFS. |
| *DeLuca 1995* | Neuropsychological impairments in chronic fatigue syndrome, multiple sclerosis, and depression | CFS (26) | MS (12); D (14); HC (20) | | U | To examine the degree and nature of cognitive impairments in CFS. | Digit span subtest of the WAIS-R - a test of "auditory span" of attention; paced auditory serial addition test (PASAT) - a test of information processing speed; trail making test -a test of visuomotor scanning and concentration; booklet category test (BCT) - complex problem solving and conceptual reasoning; vocabulary, arithmetic, similarities, and block design, subtests of the WAIS-R-tests of intellectual functions; Rey complex figure test - visual memory; California verbal learning test (CVLT) - verbal list learning and memory; logical memory subtest of the Wechsler memory scale revised - verbal paragraph recall. | The chronic fatigue syndrome group did not differ from the depressed group in overall neuropsychological performance, but differed from the multiple sclerosis and control groups. The most significant impairment was in information processing speed in the chronic fatigue syndrome group. Depression and anxiety were not related to neuropsychological performance. |

| | | | | | | | | |
|---|---|---|---|---|---|---|---|---|
| *DeLuca 1997* | Cognitive functioning is impaired in patients with chronic fatigue syndrome devoid of psychiatric disease | CFS (36) (15 no-psy; 21 psy) | HC (31) | | U | To examine the effect of the presence or absence of psychiatric disease on cognitive functioning in chronic fatigue syndrome. | Paced auditory serial addition test (PASAT) (complex information processing efficlency); vocabulary, arithmetic, and digit span subtests of the WAIS-R (intellectual and attentional skills); Rey-Osterreith complex figure test (ROCFT) (visual memory and visual-constructional ability); California verbal learning test (CVLT) (verbal memory). | The overall result showed that, relative to HC, cognition was impaired in the CFS-nopsych group (chronic fatigue syndrome subjects without a lifetime or concurrent psychiatric disorder) (Rey fig - imediate and delayed recall; CVLT, PASAT, DS-B). Patients with CFS with a concurrent axis 1 psychiatric diagnosis (CFS-psych group) did not differ from controls on individual neuropsychological tests. Further, the CFS-nopsych group also performed significantly below the CFS-psych group on several measures of memory and concentration (Rey fig - imediate and delayed recall; CVLT (short free recall), DS-B).. These data suggest that cognitive impairment in chronic fatigue syndrome cannot simply be explained by the presence of psychiatric state, and are contrary to expectations based on a model of depression induced cognitive impairment in chronic fatigue syndrome. |
| *DeLuca 1997* | Sudden vs Gradual onset of chronic fatigue syndrome differentiates individuals on cognitive and psychiatric measures | CFS (36) (11 gradual, 25 sudden) | HC (31) | | U | To examine the influence of mode of CFS onset on neuropsychological performance and psychiatric status. | Paced Auditory Serial Addition Test (PASAT), Vocabulary, Arithmetic, and Digit Span subtests of the WAIS-R, the Rey-Osterreith Complex Figure Test (ROCFT), and the California Verbal Learning Test (CVLT). | The rate of concurrent psychiatric disease was significantly greater in the CFS-gradual group relative to the CFS-sudden group. While both CFS groups showed a significant reduction in information processing ability(i.e. PASAT) relative to controls, impairment in memory was more severe in the CFS-sudden group (particularly verbal memory). |
| *DeLuca 2004* | The Nature of Memory Impairment in Chronic Fatigue Syndrome | CFS no psych (29); CFS psych (22) | HC (30); RA (19) | | C | To examine whether memory impairment in chronic fatigue syndrome (CFS) is due to deficits in acquisition, storage, or retrieval. | Verbal learning and memory were assessed using a modified version of the Selective Reminding Test, which has been used with other populations to determine whether learning and memory impairment are related to difficulties in acquisition, retrieval, or both; Visual learning and memory was | Impaired verbal learning and memory in CFS is primarily a result of deficient acquisition. |

| | | | | | | | | |
|---|---|---|---|---|---|---|---|---|
| | | | | | | | evaluated using the 7/24 test; complex information processing was assessed by means of the Paced Auditory Serial Addition Test (PASAT). | |
| *DeLuca 2004* | Working memory deficits in chronic fatigue syndrome: Differentiating between speed and accuracy of information processing | CFS no Psych (29); CFS Psych (22) | healthy subjects (29); rheumatoid arthritis patients (18) | | C | To examine the relative influence of speed of information processing versus working memory ability, CFS participants with psychiatric comorbidity (CFS–Psych) and CFS without a psychiatric history (CFS–noPsych) were examined on tests of visual and auditory processing speed and visual and auditory working memory. | Information processing speed tasks: Complex information processing speed (modifications to the PASAT) - The Auditory Threshold Serial Addition Test; Visual Threshold Serial Addition Test; Reaction time tasks measuring information processing speed. Reaction time was measured with a series of simple and choice reaction time tasks. Working memory accuracy tasks: verbal WM task; visuospatial WM task. | Compared to healthy controls (HC) and a group of participants with rheumatoid arthritis (RA), the CFS–noPsych group displayed significantly reduced performance on tests of information processing speed, but not on tests of working memory. No significant differences were observed between the CFS–Psych group and any other group in the study. |
| *DiClementi 2001* | Information processing in chronic fatigue syndrome A preliminary investigation of suggestibility | CFS (21) | HC (21) | | U | This study examines the effects of certain types of information processing on the subjective experience of cognitive deficits in persons with chronic fatigue syndrome (CFS). | Stroop Neuropsychological Screening Test; North American Adult Reading Test; Wechsler Memory Scale-Revised; Logical Memory Subtest (Immediate Recall Only); | CFS patients and healthy controls did not differ significantly on either the WMS-R logical memory immediate recall or the NAART estimated FSIQ. Thus, the two groups were equivalent in terms of both intellectual functioning and memory. There was a significant between-groups difference in which the CFS group performed more poorly than the healthy controls on a measure of automatic processing, the Stroop test. Participants with CFS showed significantly greater suggestibility on a global measure of |

| | | | | | | | | |
|---|---|---|---|---|---|---|---|---|
| | | | | | | | | suggestibility than the healthy control group. |
| *Dobbs 2001* | Working memory deficits associated with chronic fatigue syndrome | CFS (20) | HC (20) | | U | The present study investigated the cognitive functioning of chronic fatigue patients. | Forward Digit Span Test (storage capacity); Digit Span Test Backward (a measure of manipulation of information in working memory without temporal constraints); Trails A (simple sequencing task); Trails B (sequencing task that requires multiple shifts between sequences); Dobbs and Rule working memory task (emphasizing more intensive manipulation of information with temporal demands); sustained selective attention task (the resources task) (index of the ability to resist interference from background noise during processing) | Deficits were restricted to more demanding tasks, requiring resistance to interference and efficient switching between processing routines. The overall results clearly implicate deficits in the control aspects of central executive function in CFS. |

| | | | | | | | | |
|---|---|---|---|---|---|---|---|---|
| *Fiedler 1996* | A Controlled Comparison of Multiple Chemical Sensitivities and Chronic Fatigue Syndrome | CFS (18) | patients whose sensitivities to multiple low level chemical exposures began with a defined exposure (MCS) (23); patients with sensitivities to multiple chemicals without a clear date of onset (CS) (13); normal controls (18) | | C | To determine the characteristics that differentiated subjects with multiple chemical sensitivities (MCS), chemical sensitivities (CS), and chronic fatigue syndrome (CFS) and to evaluate the psychiatric and neuropsychological complaints of these groups relative to normal controls. | Simple Reaction Time, Continuous Performance Test, Digit Span, Stroop Color Word Task, Digit Symbol, Hand-Eye Coordination, Grooved Pegboard, California Verbal Learning Test, Continuous Visual Memory Test, Visual Reproduction I and II | Although MCS, CS, and CFS patients reported numerous neuropsychological symptoms, neuropsychological test results did not account for the level of impairment implied by the symptom reports. The only test showing significant differences between MCS and normal controls was a complex test of visual memory (CVMT). |
| *Fischler 1996* | Comparison of 99m Tc HMPAO SPECT scan between chronic fatigue syndrome, major depression and healthy controls: an exploratory study of clinical correlates of regional cerebral blood flow | CFS (16) | Major Depression (19); HC (20) | | U | To explore the relationship between neuropsychological symptomatology and cerebral blood flow in the chronic fatigue syndrome (CFS). | Finger tapping test, Buschke selective reminding test, Memory for location test, TMT-B, DS-B (WAIS). | 99mTc HMPAO SPECT scan reveals statistically significant positive correlations between frontal blood flow on the one hand and objectively and subjectively assessed cognitive impairment, self-rating of physical activity limitations and total score on Hamilton Depression Rating Scale on the other. A pathophysiological role of frontal blood flow in the cognitive impairment and physical activity limitations in CFS was hypothesized. A comparison of cerebral blood flow between CFS, major depression (MD) and healthy controls (HC) was performed. A lower superofrontal perfusion index is demonstrated in MD as compared with both CFS and HC. There is neither a global nor a marked regional hypoperfusion in |

| | | | | | | | | |
|---|---|---|---|---|---|---|---|---|
| | | | | | | | | CFS compared with HC. Asymmetry (R > L) of tracer uptake at parietotemporal level is demonstrated in CFS as compared with MD |
| *Fuentes 2010* | Intraindividual Variability in Cognitive Performance in Persons With Chronic Fatigue Syndrome | CFS (14) | healthy individuals (16) | The Victoria Symptom Validity Test | C | To examine intraindividual variability as well as level of performance in cognitive functioning. A battery of cognitive measures was administered to 14 CFS patients and 16 healthy individuals on 10 weekly occasions. | Reaction time task, visual search task, Stroop, word recognition, Weekly Vocabulary | CFS patients were slower but not less accurate than healthy persons. The CFS group showed greater intraindividual variability (as measured by intraindividual standard deviations and coeficients of variation) than the healthy group, although the results varied by task and time frame. Intraindividual variability was found to be stable across time and correlated across tasks at each testing occasion. Intraindividual variability also uniquely differentiated the groups. intraindividual variability appears to be a meaningful indicator of cognitive functioning in CFS patients. |

| | | | | | | | | |
|---|---|---|---|---|---|---|---|---|
| *Gaudino 1997* | Post-Lyme Syndrome and Chronic Fatigue Syndrome | CFS (25) | patients with PLS (38); healthy controls (56) | | U | To examine the neuropsychiatric differences inCFS and PLS to enhance understanding of how mood, fatigue, and cognitive performance interrelate in chronic illness. | General cognitive functioning - Wechsler Adult Intelligence Scale-Revised (WAISR) Vocabulary and Information subtests; attention - WAIS-R Digit Span and Digit Symbol subtests and the Trail-Making Test; verbal memory - Selective Reminding Test (SRT) and the Wechsler Memory Scale-Revised Logical Memory subtest; visual memory - Benton Visual Retention Test; Verbal fluency - Controlled Oral Word Association; The Finger Tapping test - fine motor speed. | Patients with CFS and PLS were similar in several somatic symptoms and in psychiatric profile. Patients with CFS reported more flulike symptoms than patients with PLS. Patients with PLS but not patients with CFS performed significantly worse than controls on tests of attention, verbal memory, verbal fluency, and motor speed. Patients with PLS without a premorbid history of psychiatric illness did relatively worse on cognitive tests than patients with PLS with premorbid psychiatric illness compared with healthy controls.<br>Despite symptom overlap, patients with PLS show greater cognitive deficits than patients with CFS compared with healthy controls. This is particularly apparent among patients with PLS who lack premorbid psychiatric illness. |
| *Gotts 2015* | The Association between Daytime Napping and Cognitive Functioning in Chronic Fatigue Syndrome | CFS (118) | | | U | This study aimed to examine self-reported sleep in patients with CFS and explore whether sleep quality and daytime napping, demographic characteristics and levels of anxiety and depression, predicted cognitive functioning, daytime fatigue severity and levels of daytime sleepiness. | Trail Making Test | Napping, particularly in the afternoon is associated with poorer cognitive functioning and more daytime sleepiness in CFS. |

| | | | | | | | | |
|---|---|---|---|---|---|---|---|---|
| *Grafnan 1993* | Analysis of neuropsychological functioning in patients with chronic fatigue syndrome | CFS (20) | HC (17) | Subjective judgement | U | To examine objective cognitive performance including memory in CFS. | General intelectual functioning - Wechsler adult intelligence scale revised; timing and reaction time tasks - Simple reaction time, Serial reaction time test, Time wall, Time clock; problem solving and planning - Tower of London, Tower of Hanoi, Twenty questions; Memory - Wechsler memory scale revised; Experimental paired associate test; Hasher frequency monitoring task; Story memory; Word fluency; | The results on objective testing indicated that the CFS patients had some mild memory impairment, but only on tasks requiring conceptually driven encoding and retrieval processes. There were no associations between the nature of the precipitating illness, self ratings of fatigue, physical findings, or laboratory determination and objective memory performance or self report of memory functioning. These generally negative results indicate that memory impairment in CFS patients is typically mild and involves memory processes that participate in conceptualising information. |
| *Hou 2008* | Attentional bias towards health-threat information in chronic fatigue syndrome | CFS (14) | HC (18) | | C | To investigate whether individuals with chronic fatigue syndrome (CFS) show an attentional bias towards health threat information. | Visual probe task | Compared to a healthy control group, the CFS group showed an enhanced AB towards heath-threat stimuli relative to neutral stimuli. The AB was not influenced by the type of stimulus (pictures vs. words). |
| *Hou 2014* | Attention processes in chronic fatigue syndrome: Attentional bias for health-related threat and the role of attentional control | CFS (27) | HC (35) | | C | To investigate attentional bias towards health-threat stimuli in CFS and to examine whether individuals with CFS have impaired executive attention, and whether this was related to attentional bias. | Visual Probe Task; Attention Network Test | Compared to the control group, the CFS group showed greater attentional bias for health-threat words than pictures; and the CFS group was significantly impaired in executive attention. Furthermore, CFS individuals with poor executive attention showed greater attentional bias to health-threat related words, compared not only to controls but also to CFS individuals with good executive attention. Thus, this study revealed a significant relationship between attentional bias and executive attention in CFS: attentional bias to threat was primarily evident in those with impaired executive attention control. |

| | | | | | | | | |
|---|---|---|---|---|---|---|---|---|
| *Ickmans 2013* | Cognitive performance is of clinical importance, but is unrelated to pain severity in women with chronic fatigue syndrome | women with CFS (29) | healthy controls (17) | | C | This study investigated the relationship between cognitive performance and pain, fatigue, self-reported symptoms and health status in women with CFS. | Psychomotor vigilance task, Stroop task, Operation span task | The data indicate that adult female CFS patients with objectively demonstrated cognitive problems self-report more cognitive problems and vice versa. These findings are very important, since people with CFS are often suspected to exaggerate their symptoms, and this study proves the opposite by demonstrating the presence of a relationship between selfreported cognitive problems and results of performance-based cognitive testing in adult female CFS patients. Furthermore, while the level of fatigue in the previous week(s) was not correlated with cognitive performance in these patients, higher levels of fatigue at the time of cognitive testing were associated with less sustained attention. Finally, this study is the first to examine the relationship between cognitive performance and pain severity in CFS patients. In contrast to the findings in several other chronic pain populations, the results of the present study reveal no relationship between pain severity and cognitive functioning. |
| *Ickmans 2014* | Can Recovery of Peripheral Muscle Function Predict Cognitive Task Performance in Chronic Fatigue Syndrome With and Without Fibromyalgia? | CFS FM (30) | CFS only (18); healthy and inactive controls (30) | | C | To examine whether recovery of upper limb muscle function could be a significant predictor of cognitive performance in patients with CFS and patients with CFS and comorbid fibromyalgia, and compare cognitive performance in both groups. | Stroop task; psychomotor vigilance task (PVT); operation span (OSPAN) task | Participants in the CFS FM group but not those in the CFS group showed significantly decreased cognitive performance compared with the control group. Recovery of upper limb muscle function was found to be a significant predictor of cognitive performance in patients with CFS. |

| | | | | | | | | |
|---|---|---|---|---|---|---|---|---|
| *Ickmans 2015* | Associations Between Cognitive Performance and Pain in Chronic Fatigue Syndrome: Comorbidity with Fibromyalgia Does Matter | CFS (18) | CFS+FM (30); HC (30) | | C | To examine the association between cognitive performance and self-reported as well as experimental pain measurements in CFS patients with and without FM. | Stroop task, psychomotor vigilance task (PVT) and operation span (OSPAN) task. | CFS+FM group showed significantly decreased RTs, cognitive inhibition, and selective and sustained attention in comparison with the control group. Although the CFS group also showed worse results on cognitive tests compared with the control group, these differences were only significant for simple RT. In the CFS+FM group, the capacity of pain inhibition was significantly associated with cognitive inhibition. Self-reported pain was significantly associated with simple reaction time in CFS-only patients. The CFS+FM but not the CFS-only group showed a significantly lower PPT and enhanced TS compared with controls. The results underline disease heterogeneity in CFS by indicating that a measure of endogenous pain inhibition might be a significant predictor of cognitive functioning in CFS patients with FM, while self-reported pain appears more appropriate to predict cognitive functioning in CFS patients without FM |
| *Johnson 1994* | Cognitive Functioning of Patients with Chronic Fatigue Syndrome | CFS (22) | age- and education-matched healthy subjects (21) | | U | To assess verbal memory in patients with CFS subjects. | California Verbal Learning Test (CVLT) | There were no differences between the subjects with CFS and the healthy subjects on any of the CVLT variables, including acquisition, retrieval, rate of forgetting, or recognition of the list of words (table I). These results suggest that there is no primary verbal memory impairment in subjects with CFS. It was hypothesized that despite patients' complaints o fmemory impairment, verbal memory may not be the primary issue. |

| | | | | | | | | |
|---|---|---|---|---|---|---|---|---|
| *Johnson 1996* | Selective impairment of auditory processing in chronic fatigue syndrome: a comparison with multiple sclerosis and healthy controls | CFS (20) | Multiple Sclerosis (20); sedentary healthy controls (20) | | C (partially) | The most consistent deficit observed in individuals with Chronic Fatigue Syndrome has been in efficiency of information processing. To examine the possibility of a modality-specific impairment, the present study examined subjects with Chronic Fatigue Syndrome, multiple sclerosis, and healthy controls on an auditory versus visual-paced serial-addition test. | Digit Span Forward and Digit Span Backward of the Wechsler Adult Intelligence Scale-Revised (WAIS-R); Paced Auditory Serial Addition Test; Paced Visual Serial Addition Test; | Patients Chronic Fatigue Syndrome showed impaired performance on the Paced Auditory Serial Addition Test. However, subjects with Chronic Fatigue Syndrome did not score lower than healthy subjects on the Paced Visual Serial Addition Test, a task that requires processing of material presented visually. These data suggest a selective impairment in individuals with Chronic Fatigue Syndrome in processing auditory material. In contrast to the Chronic Fatigue Syndrome group, the Multiple Sclerosis group was equally impaired in the processing of both the visual and auditory Serial Addition Tests. |
| *Johnson 1997* | The Effects of Fatigue on Neuropsychological Performance in Patients With Chronic Fatigue Syndrome, Multiple Sclerosis, and Depression | CFS (15) | multiple sclerosis (15); depression (14); healthy, sedentary controls (15) | | U | To examine the effects of fatigue on neuropsychological performance were examined in patients with CFS. To produce fatigue by a challenging cognitive task over time, participants were exposed repeatedly to the Paced Auditory Serial Addition Test during a demanding 3-hr neuropsychological testing session. | Paced Auditory Serial Addition Test | Overall PASAT performance was significantly reduced for CFS and DEP participants compared to controls whereas mean performance did not differ across the three fatiguing illness groups. Degree of improvement across trials (i.e., practice effect) for the groups did not differ from controls. |

| | | | | | | | | |
|---|---|---|---|---|---|---|---|---|
| *Johnson 1998* | Memory Dysfunction in Fatiguing Illness: Examining Interference and Distraction in Shortterm Memory | CFS (23) | MS (15); D (14); HC (17) | | C | To examine the hypothesis that three fatiguing illness groups are more vulnerable to semantic interference and distraction in short-term memory and this vulnerability is related to recall deficits in long-term memory. | Proactive interference; Digit span subtest from the WAIS-R (attention and short-term memory); North American Adult Reading Test (verbal intelligence); Short-term Memory Distractor Task; | There were no significant differences in build-up and release from proactive interference relative to controls, although the CFS and MS groups recalled significantly fewer words overall. All three fatigue groups evidenced recall impairment after a brief distractor. Furthermore, brief distraction resulted in impaired immediate and delayed recall in the MS and CFS groups compared to controls. Results indicate that fatiguing illness groups, particularly MS and CFS, are vulnerable to limited disruption in short-term memory processing and this can affect recall |
| *Joyce 1996* | Memory, attention, and executive function in chronic fatigue syndrome | CFS (20) | matched normal controls (20) | | C | To assess cognitive function in CFS. | CANTAB computerised tests of visuospatial memory, attention, and executive function, and verbal tests of letter and category fluency and word association learning; word association learning subtest of the Wechsler memory scale revised; | Performance of patients with CFS was impaired, predominantly in the domain of memory. However pattern of abnormalities was different from patients with amnesic syndrome or dementia. They performed normally on tests of spatial and pattern recognition memory, simultaneous and delayed matching to sample, and pattern-location association learning. They were impaired on tests of spatial span, spatial working memory, and a selective reminding condition of the pattern-location association learning test. An executive test of planning was normal. In an attentional test, eight subjects with chronic fatigue syndrome were unable to learn a response set; the remainder exhibited no impairment in the executive set shifting phase ofthe test. Patients with chronic fatigue syndrome were also impaired on verbal tests of unrelated word association learning and letter fluency. In conclusion, patients with chronic fatigue syndrome showed reduced attentional capacity resulting in impaired performance on effortful tasks requiring planned or self ordered |

| | | | | | | | | |
|---|---|---|---|---|---|---|---|---|
| | | | | | | | | generation of responses from memory. |
| *Kane 1997* | Neuropsychological and psychological functioning in chronic fatigue syndrome | CFS (17) | HC (17) | | U | To compare CFS and HC in terms of attention, memory, processing efficiency and learning. | 6 measures of attention, memory, and word-finding ability (gordon task - sustained attention, PASAT, information processing speed and efficiency, digit symbol subtest from WAIS-R; verbal learning and memory, non-verbal learning and memory, word finding); | No significant differences in cognitive performance between CFS and closely matched HC (CFS patients performed worse in only 2 of 16 measures - immediate recall and digit symbol subtest of WAIS-R). However, CFS patients differed markedly from controls in reported psychological distress. |

| | | | | | | | | |
|---|---|---|---|---|---|---|---|---|
| *Krupp 1994* | Cognitive Functioning and Depression in Patients With Chronic Fatigue Syndrome and Multiple Sclerosis | CFS (20) | MS (20); HC (20) | | U | To assess cognitive function in patients with chronic fatigue syndrome (CFS) and multiple sclerosis (MS) and to evaluate the role of depressive symptoms in cognitive performance. | Premorbid verbal ability' - Information and Vocabulary subtests of the Wechsler Adult Intelligence Scalesrevised (WAIS-R) and the reading portion ofthe Wide Range Achievement Test-revised; Spatial-constructional skills - Object Assembly and Block Design WAIS-R subtests; Attention and concentration - Stroop Color and Word Test and the WAIS-R Digit Span subtest; Visuomotor search - Trail Making Test parts A and B, Digit Symbol subtest, Symbol-Digit Modalities Test; flexibility in shifting from one task to another - Symbol-Digit Modalities Test (oral); Abstract reasoning - Booklet Category Test; Verbal memory functions - six-trial version of the Selective Reminding Test and the paired associate learning and logical memory measures of the Wechsler Memory Scale-revised; visual memory - Benton Visual Retention Test; verbal fluency - Controlled Oral Word Association Test; motor speed - Finger Oscillation Test. | Patients with CFS compared with MS have more depressive symptoms but less cognitive impairment. Relative to controls, a subset of CFS subjects did poorly on tests of visuomotor search and on the logical memory measure of the Wechsler Memory Scale - revised.Poor performance of logical memory in CFS appears to be related to depression, while visuomotor deficits in CFS are unrelated. Cognitive deficits in patients with MS are more widespread compared with those in patients with CFS and are independent of depressive symptoms. |

| | | | | | | | | |
|---|---|---|---|---|---|---|---|---|
| *Lakein 1997* | Patients with Chronic Fatigue Syndrome and Accurate Feeling-of-Knowing Judgments | CFS (17) | HC (17) | | C | To assess meetamemory in patients with CFS. Metamemory has been defined as knowledge of one's own memory performance and stored knowledge. Aspects of metamemory have been referred to as feeling-of-knowing (FOK) or the tip-of-the-tongue (TOT) experience. | Trivia Information Quiz (TIQ) - This test was developed to assess the long-term memory of previously learned information and to detect any discrepancies between recall and recognition. | Most CFS patients tested in this study complained of cognitive deficits, as is typical of such individuals. Their complaints included memory problems, word generation difficulty, and easy distractibility when asked to complete more than one task concurrently. The present studies, though, revealed that CFS patients performed just as well as their matched controls on all of the measures of metamemory, as assessed by the TIQ task. Both groups answered the same number of questions, thereby showing no differences in their abilities to access long-term semantic factual information. Importantly, confidence level ratings did not differ significantly between groups. Moreover, both groups showed equivalent abilities to Figure 3. Accuracy of Response: Trivia Information Quiz (TIQ). There were no between-group differences in accuracy. |
| *LaManca 1998* | Influence of Exhaustive Treadmill Exercise on Cognitive Functioning in Chronic Fatigue Syndrome | women with CFS (19) | CON (20) | | U | The purpose of this study was to determine the effect of exhaustive exercise on cognitive performance of patients with chronic fatigue syndrome (CFS) and sedentary healthy controls (CON). A test battery consisting of 4 cognitive tests (CTB) was given pre-, immediately post-, and 24 hours post-treadmill exercise to exhaustion. | Stroop Color and Word Test (cognitive speed and disinhibition); "Symbol Digit Modalities Test" ( psychomotor speed and vigilance); Trail Making Test (TMT) (psychomotor speed and attention/concentration); Serial 13s Test (STT); | No differences were seen on the CTB pre-exercise. CFS patients improved at a slower rate than controls on the Symbol Digit Modalities Test (SDMT), Stroop Word Test (SWT), and Stroop Color Test (SCT). When compared with controls a lower number of correct responses was seen for the CFS immediately postexercise on the SDMT, SWT, and SCT, and 24 hours postexercise on the SDMT, SWT, and SCT. They concluded that after physically demanding exercise, CFS subjects demonstrated impaired cognitive processing compared with healthy individuals. |

| | | | | | | | | |
|---|---|---|---|---|---|---|---|---|
| *Lange 2005* | Objective evidence of cognitive complaints in Chronic Fatigue Syndrome: A BOLD fMRI study of verbal working memory | Study 1 - CFS (6); Study 2 - CFS (19) | Study 1 - HC (7); Study 2 - HC (15) | Indirect cues | C | In two Blood Oxygen Level Dependent (BOLD) functional Magnetic Resonance Imaging (fMRI) studies, they compared BOLD signal changes between Controls and individuals with CFS who had documented difficulties in complex auditory information processing (auditory monitoring test measuring simple attention) (Study 1) and those who did not (Study 2) in response to performance on a simple auditory monitoring and a complex auditory information processing task | modified Paced Auditory Serial Addition Test - mPASAT | Individuals with CFS are able to process challenging auditory information as accurately as Controls but utilize more extensive regions of the network associated with the verbal WM system. Individuals with CFS appear to have to exert greater effort to process auditory information as effectively as demographically similar healthy adults. These findings provided objective evidence for the subjective experience of cognitive difficulties in individuals with CFS. |

| | | | | | | | | |
|---|---|---|---|---|---|---|---|---|
| *Lawrie 2000* | The difference in patterns of motor and cognitive function in chronic fatigue syndrome and severe depressive illness | CFS (30) | MDD (20); healthy controls (15) (matched group-wise for age and sex) | | C (partially) | We have examined a group of CFS patients with measures of isometric strength, subjective effort, mood and an appropriate series of neuropsychological tests, in the morning and evening, and compared them with groups of both depressed and healthy controls. | CFS and HC groups - Block Design subtest of WAIS-R, Verbal Fluency, Trails A andB, Paired Associates Learning, the Boston Naming Test, the 2- and 4-second versions of the Paced Auditory Serial Addition Test (PASAT), and the California Verbal Learning Test (CVLT). CFS and HC groups completed a number of tests in both the morning and evening on matched, parallel versions of: digit span forwards and backwards; reaction time measures from the Cambridge Neuropsychological Test Automated Battery (CANTAB); and a sustained hand grip test; Immediately after the latter, subjects were asked to complete the Borg effort scale. The remaining cognitive and motor tasks were completed by all subjects in both the morning and evening: Digit Symbol Substitution Test (DSST), Auditory Verbal Learning Test, including the delayed recall and recognition sections (AVLT). | CFS patients generally performed worse on cognitive tests than healthy controls, but better than patients with MDD. Both patient groups had markedly impaired motor function compared with healthy controls. MDD subjects showed a significantly greater diurnal improvement in maximal voluntary contraction than healthy controls. |
| *Maher-Edwards 2011* | Metacognitions and negative emotions as predictors of symptom severity in chronic fatigue syndrome | CFS (96) | | | | The aim of this study was to determine whether metacognitions are associated with symptom severity in CFS. | Metacognitions Questionnaire | Metacognitions and negative emotions were positively correlated with measures of symptom severity. Metacognitions were a better predictor of symptom severity than anxiety and depression. Lack of cognitive confidence predicted both mental and physical factors of the CFQ and physical functioning independently of negative emotions. Beliefs about the need to control thoughts predicted the mental factor of the CFQ independently of |

| | | | | | | | | |
|---|---|---|---|---|---|---|---|---|
| | | | | | | | | negative emotions and lack of cognitive confidence. |
| *Mahurin 2004* | Cognitive Processing in Monozygotic Twins Discordant for Chronic Fatigue Syndrome | CFS twin (21) | non-CFS twin pair (21); HC (21) | | C | To further explore possible genetic contributions to cognitive dysfunction in CFS, a cotwin control study of monozygotic twins was performed, in which one member of the pair had CFS and the other twin was healthy. | Computer-based NeuroCognitive Assessment Battery - selected tests emphasized measurement of information-processing speed and efficiency, as well as verbal abilities, memory, and reasoning: The Verbal Memory Test (recent verbal memory); The Visual Memory Test (recent visual memory); The Vocabulary Test; Logical Reasoning Test; Visual Conceptual Reasoning Tes; The Finger Tapping Test (motor speed and coordination); The Simple Reaction Time Test ( simple attention, cognitive speed, and response time); The Choice Reaction Time Test - (selective attention, speed of cognitive choice, and time of response to an unpredictable stimulus); Stroop Color–Word Test (Interference subtest) (selective attention and response speed); The Adaptive Rate Visual Processing Test (sustained attention, information-processing speed, and response time) | Random effects regression showed no difference between CFS and healthy twins on any of the cognitive tests. Further, the twin groups did not differ from the HC group on any content-dependent measure. In contrast, both sets of twins performed worse than the HC group on all speed-dependent tests except Finger Tapping. Self-rated fatigue and dysphoric mood were only weakly correlated with cognitive performance. These data point toward a shared genetic trait related to information processing that is manifest in the CFS context. |

| | | | | | | | | |
|---|---|---|---|---|---|---|---|---|
| *Majer 2008* | Neuropsychological Performance in Persons With Chronic Fatigue Syndrome: Results From a Population-Based Study | CFS (58) | HC (104) | | C | To examine the neuropsychological function characterized in subjects with chronic fatigue syndrome (CFS) at the same time controlling for relevant confounding factors. | Neuropsychological Testing - Wide Range Achievement Test 3 (WRAT-3) (General intellectual ability); Seven CANTAB tests were used to measure specific neuropsychological domains: Psychomotor Speed - RT test; Attention - RVIP test; Working Memory - The Spatial Working Memory test; The Pattern Recognition Memory (PRM) test; The Spatial Recognition Memory (SRM) test; Executive Function - The Stockings of Cambridge task; The Intra/Extra Dimensional Set Shift (IED) task; | Compared with controls, CFS subjects exhibited significant decreases in motor speed as measured in the simple and five-choice movement segments of the CANTAB reaction time task. CFS subjects also exhibited alterations in working memory as manifested by a less efficient search strategy on the spatial working memory task, fewer % correct responses on the spatial recognition task, and prolonged latency to a correct response on the pattern recognition task. A significantly higher percentage of CFS subjects versus controls exhibited evidence of neuropsychological impairment in tasks of motor speed and spatial working memory. |
| *Marcel 1996* | Cognitive deficits in patients with chronic fatigue syndrome | CFS (29) | healthy control subjects (25) | | C (partially) | To compare a group of CFS patients (unselected for cognitive complaints) to a group of age- and education-equivalent controls on a wide variety of cognitive tasks. Psychiatric symptomatology was assessed and a score quantifying its prevalence was used as a covariate of the neuropsychological test scores to determine whether the presence or absence of cognitive impairments in the patients is related to | Standard neuropsychological battery: attention - digit span forward; language ability - Boston Naming Test and verbal fluency; memory - Russell version of the Wechsler Memory Scale and a word list learning test; set shifting and conceptualization (aspects of executive function) - Stroop Interference Test and Proverb Interpretation; figure copying utilized figures from the Wechsler Memory Scale; overall IQ was estimated by means of the Vocabulary subtest of the WAIS. Computerized battery: continuous performance task, based on tests of sustained attention - attentional capacity; associate learning test - verbal memory; pattern memory test - nonverbal | The primary significant differences between patients and controls were found on tests of learning and memory. These differences remained when the degree of psychiatric symptomatology in the subjects was covaried. Patients on and off psychoactive medications did not differ in their performance on these tasks. These results suggest that at least a subset of CFS patients may experience significant impairments in learning and memory. |

| | | | | | | | | |
|---|---|---|---|---|---|---|---|---|
| | | | | | | coexistent psychiatric symptoms. | memory; spatial ability - pattern matching and a hand-eye coordination test; set shifting - adaptation of a switching attention task | |
| *Marshall 1996* | An Assessment of Cognitive Function and Mood in Chronic Fatigue Syndrome | CFS (27) | subjects participating in a study of the effects of allergy season on mood and cognitive function: atopic control subjects (10); healthy control subjects (8) | | | To investigate how CFS correlated with motor speed and speed of cognitive processing, the ability to sustain attention, and daily mood. To ascertain whether CFS patients have cognitive deficits or a mood profile similar to individuals with major depression. Data before before starting terfenadine or placebo were used for these comparisons. | Sustained Attention - Continuous Performance Test - Identical Pairs version (CPT-IP); reaction time tests | CFS patients exhibited slower cognitive processing and motor speed and lower positive affect, as compared to data reported from previous studies of healthy subjects and other patient groups; however, CFS patients did not exhibit deficits in sustained attention in comparison to other groups. The CFS patients' ability to attend to verbal versus figural stimuli and mood ratings were different from those reported in studies of patients with depression. Because of methodological limitations, these findings are preliminary, but they encourage further assessment of cognitive dysfunction and mood in CFS. |

| | | | | | | | | |
|---|---|---|---|---|---|---|---|---|
| *Marshall 1997* | Cognitive Slowing and Working Memory Difficulties in Chronic Fatigue Syndrome | CFS (20) | healthy control subjects (20); patients with a history of major depression or dysthymia (14) matched by age, intelligence, education level, and sex | Indirect cues | U / C (partially) | This study was undertaken to examine further the hypothesis that CFS patients exhibit slowing of motor and cognitive processing speed. A test battery composed of six tests assessing these cognitive functions was given on two consecutive days. CFS patients were tested on two consecutive days to investigate their common complaint that, although they can "rise to the occasion" and do challenging tasks on one day, they typically feel extremely fatigued and ineffective the next day. | Buschke Selective Reminding Test; Continuous-Performance Test-Identical Pairs Version (CPT) (test for sustained attention); Paced Auditory Serial Addition Task (PASAT) (cognitive processing speed, sustained attention, divided attention, and working memory) ; Stroop Color Word Test (cognitive processing and motor speed as well as focused attention); Reaction-Time Tests; Salthouse Reading Span Task (SRST) (working memory) ; Verbal Scholastic Aptitude Test (SAT). | Compared with control subjects, CFS patients consistently scored lower on tests in which motor and cognitive processing speeds were a critical factor, eg, reaction-time tasks. They also had more difficulty on working-memory tests in which rapid cognitive processing speed is also an important factor. The effort made on the first day of testing did not result in a decline in cognitive function on the following day. CFS patients did not qualify as having affective disorder by several different diagnostic criteria. Nonetheless, CFS patients' test performances did not differ from patients with a history of major depression or dysthymia. It was concluded that, although CFS and major depression and dysthymia have distinct clinical features, these disorders have slowed motor and cognitive processing speed in common. |
| *Martin 2010* | Mood volatility with rumination but neither attentional nor interpretation biases in chronic fatigue syndrom e | CFS (33) | HC (33) | | C | This study tested whether: patients with CFS have a bias in the initial orientation of attention to illness-related information, which is enhanced by rumination; CFS individuals have an illness interpretation bias (IB) in their early automatic | All participants underwent either a rumination or distraction induction. They then completed an exogenous cueing task to assess bias to illness and social threat compared with neutral stimuli, as well as a lexical decision task to assess their interpretation of ambiguous words having illness, social threat, or neutral interpretations. | Reaction time data revealed that CFS individuals did not have an attentional bias (AB) in the initial orientation of attention to illness-related material. Nor was there an IB towards illness in CFS individual's automatic response to ambiguous information. However, as hypothesized, CFS individuals showed a greater degree of mood fluctuation following the rumination/distraction induction. They concluded that rumination and distraction lead to greater mood volatility in CFS individuals than in controls, but not to attentional nor |

| | | | | | | | | |
|---|---|---|---|---|---|---|---|---|
| | | | | | | processing of ambiguous information; CFS individuals experience a greater degree of mood fluctuation following rumination and distraction inductions. | | interpretation biases in the early automatic stages of information processing in CFS individuals. |
| *McCue 2002* | Validation of a telephone cognitive assessment test battery for use in chronic fatigue syndrome | CFS (30) | healthy controls (30) matched for age and education | | C | To compare a computerized version of the Cognitive Drug Research (CDR) cognitive assessment test battery and a completely automated telephone version of the same battery. These assessed aspects of attention, working memory and long-term memory. | Simple reaction time; Word presentation; Choice reaction time; Numerical working memory; Word recognition | The CFS group had significantly slower reaction times on all four cognitive measures on both the computerized and telephone tests. The mood data followed similar patterns in the computer and telephone assessments. The results from both forms of the test battery confirmed the pattern and severity of cognitive impairment in CFS. Furthermore, the two methods of testing were similarly sensitive in detecting cognitive deficits. The incapacitating nature of CFS may cause problems for researchers if the restrictions to mobility affect the representativeness of the study group. The findings of the present study support the use of a fully automated telephone cognitive testing system for detecting deficits in CFS. |
| *McDonald 1993* | Cognitive impairment in patients with chronic fatigue: a preliminary study | CF (65) | | | | The aims of the study were: 1) to determine the prevalence of subjective impairment of cognitive functioning in an unselected sample of patients with chronic fatigue; 2) | Concentration was tested with serial 7s, and digit span (DS) forwards and backwards; visual attention - star cancellation test; memory - paired associate learning test from the WMS The forwards DS, easy paired associates, and star cancellation allow for relatively automatic processes to be examined while the remainder | Subjective cognitive impairment was strongly related to psychiatric disorder, especially depressed mood, but not fatigue, anxiety, or objective performance. Simple tests of attention and concentration showed some impairment but this was influenced by both fatigue and depression. Subjects with high levels of fatigue performed less well on a memory task requiring cognitive effort, even in the absence of depression. |

| | | | | | | | | |
|---|---|---|---|---|---|---|---|---|
| | | | | | | to screen for the presence of objective impairment of cognitive functioning; 3) to examine the relationship between psychiatric morbidity-especially depressed mood and anxiety-and cognitive dysfunction, both subjective and objective. | require more cognitive effort. | There was no evidence for mental fatiguability. |
| *Metzger 2002* | Perception of Cognitive Performance in Patients With Chronic Fatigue Syndrome | CFS (40) | HC (40) | Indirect cues | C | This study examined discrepancies between perceived and actual performance by patients with chronic fatigue syndrome (CFS) confronted with a challenging cognitive task. Before and after completing a modified version of the Stroop Color-Word Interference Test, CFS patients and healthy control participants estimated their own performance and the performance that would normally be achieved by someone of equal age and education | Stroop Color-Word Interference Test | After correcting for differences between the groups in depression, there were no differences in actual performance on the Stroop. However, patients with CFS consistently underestimated their performance relative to normal performance. This difference was observed for both depressed and nondepressed subgroups of patients, persisted after adjusting the results for depression, and correlated with patients' ratings of the mental effort and fatigue evoked by the task. The results are discussed in light of cognitive models of CFS that suggest the setting of impossibly high standards of personal performance may contribute to the dynamism of this disease. |

| | | | | | | | | |
|---|---|---|---|---|---|---|---|---|
| | | | | | | level. | | |
| *Michiels 1996* | Cognitive Functioning in Patients with Chronic Fatigue Syndrome | CFS (35) | controls were matched for gender, age, intelligence and education (33) | Subjective judgement of researchers | U | To assess whether: 1. Patients with CFS perform worse on selected tests of psychomotor speed, verbal/ visual memory, and attention as compared to normal controls? If so, what is the specific nature of these cognitive impairments? Is their performance related to depression? 2. What is the percentage of patients with CFS who present with cognitive dysfunction and how severe is this impairment? 3. Can we identify neuropsychological variables that discriminate best between patients with CFS and normal controls? | Finger Tapping Test, Selective Reminding Test, Memory for Location Test, Trail Making Test, WAIS Digit Span, and WAIS Digit Symbol. The short form of the Standard Progressive Matrices and WAIS Vocabulary were administered to estimate intellectual ability. | The patients displayed psychomotor slowing and impaired attention. The learning rate of verbal and visual material for patients with CFS was slower, and delayed recall of verbal and visual information was impaired. Because there was a high variability in cognitive impairment within the CFS group, it would be inappropriate to generalize results to the entire CFS population. Two neuropsychological variables indicating aspects of psychomotor performance and verbal memory were found to discriminate best between patients and controls. |

| | | | | | | | | |
|---|---|---|---|---|---|---|---|---|
| *Michiels 1998* | Attention and verbal learning in patients with Chronic Fatigue Syndrome | CFS (20) | HC (22) | | C (partially) | To evaluate attentional functions more extensively and to clarify the verbal learning ability in patients with CFS. The following questions are addressed: 1. Is there objective evidence for impaired attention and verbal memory among patients with CFS as compared to healthy controls, and what is the nature of this problem? 2. Are depression, anxiety, subjective fatigue, and cognitive complaints related to test performance in this CFS sample? 3. Does medication affect test performance? | WAIS Digit Span, Trail Making Test (TMT), Stroop Test (ST), Simple Reaction Time (SRT), Choice Reaction Time for Single Digits (CRT), and Sequential Reaction Time (SQRT). Verbal memory was evaluated with the California Verbal Learning Test (CVLT). | The results provide evidence for attentional dysfunction in patients with CFS as compared to HC. CFS patients performed more poorly on a span test measuring attentional capacity and working memory. Speeded attentional tasks with a more complex element of memory scanning and divided attention seem to be a sensitive measure of reduced attentional capacity in these patients. Focused attention, defined as the ability to attend to a single stimulus while ignoring irrelevant stimuli, appears not to be impaired. CFS patients were poorer on recall of verbal information across learning trials, and poor performance on delayed recall may be due to poor initial learning and not only to a retrieval failure. |
| *Michiels 1999* | Attention and Information Processing Efficiency in Patients with Chronic Fatigue Syndrome | CFS (29) | HC (22) matched for age, gender, intelligence , and education | | C (partially) | In this study a battery of attentional tests and a verbal memory task were administered to outpatients with Chronic Fatigue Syndrom e (CFS) in order to assess attention and memory function and in particular to | Simple RT paradigm with variable preparatory intervals (PI); Posner' s RT paradigm of covert orientation of attention; Sternberg' s memory scanning paradigm; the Paced Auditory Serial Addition Test and a visual equivalent version; Verbal memory was evaluated with a verbal list-learning task using a Selective Reminding Procedure. | The results show that patients with CFS do not seem to be impaired for modification of phasic arousal level, nor for visual selective attention requiring shifting of attention in the visuospatial field. The results further support the presence of reduced information processing speed and efficiency, and strengthen the evidence of a global non-modality-specific attentional dysfunction in patients with CFS. In this study the poor performance of patients with CFS on recall of verbal information was due to |

| | | | | | | | | |
|---|---|---|---|---|---|---|---|---|
| | | | | | | investigate the rate of cognitive processing independent of motor speed and the possibility of a modality-specific impairment of information processing. | | poor initial storage rather than to a retrieval failure. |
| *Moss-Morris 2003* | Experimental evidence for interpretive but not attention biases towards somatic information in patients with chronic fatigue syndrome | CFS (25) | healthy matched controls (24) | Indirect cues | C | This study tested whether CFS patients have an attentional information processing bias for illness-related information and a tendency to interpret ambiguous information in a somatic fashion. | Modified CFS Stroop task - A modified CFS Stroop task was developed to determine whether somatic relevant words were more distracting to CFS patients than depressed relevant words; National Adult Reading Test; Ambiguous cues task | Although CFS patients were significantly slower in colour naming all of the Stroop word categories than controls, there was no evidence for illness or depressed words creating greater interference than neutral words. However, on the ambiguous cues task, CFS patients made significantly more somatic interpretations than controls and this bias was significantly associated with the extent to which they currently reported symptoms. In conclusion, CFS patients have an interpretive bias for somatic information which may play a part in the maintenance of the disorder by heightening patients' experience of physical symptoms and helping to maintain their negative illness schemas. Although patients did not show an attentional bias in this study, this may be related to the methodology employed. |

| | | | | | | | | |
|---|---|---|---|---|---|---|---|---|
| *Moss-Morris 2011* | The pathway from glandular fever to chronic fatigue syndrome: can the cognitive behavioural model provide the map? | *glandular fever (246) | | | | The purpose of this study was to investigate whether psychological factors could predict the onset of CFS following an acute episode of infectious mononucleosis. | | Of the participants, 9.4% met the criteria for CF at 3 months and 7.8% met the criteria for CFS at 6 months. Logistic regression revealed that factors proposed to predispose people to CFS including anxiety, depression, somatization and perfectionism were associated with new-onset CFS. Negative illness beliefs including perceiving GF to be a serious, distressing condition, that will last a long time and is uncontrollable, and responding to symptoms in an all-or-nothing behavioural pattern were also significant predictors. All-or-nothing behaviour was the most significant predictor of CFS at 6 months. Perceived stress and consistently limiting activity at the time of GF were not significantly associated with CFS. |
| *Neu 2011* | Cognitive impairment in fatigue and sleepiness associated conditions | CFS (pure fatigue) (15) | SAHS (without fatigue) (15); HC (15) | | U | To compare the potential cognitive impairment profiles, assessing easy-to-use routine tasks of attention, memory and psychomotor performance parameters in two patient groups, with an EDS complaint (SAHS patients) or a clinically significant fatigue complaint (CFS patients) respectively. | Rey auditory verbal learning test (AVLT); digit span subtest of WAIS;, digit symbol substitution test and finger tapping test (FTT) were used as cognitive and behavioural measures. | With exception for the digit span, all tests showed lower performances in patient groups. Recall on the AVLT did not differ between the two patient groups, but the digit and symbol spans showed more severe impairment in SAHS patients. Psychomotor performance on the FTT presented with slower hit rates in SAHS than in CFS. EEG theta power was highest in CFS patients. P300 latencies and amplitudes did not differ between groups. Fatigue– and sleepiness–associated conditions can both present with significant and objective impairment of cognitive functioning and behavioural motor performance. In their sample cognitive impairment and psychomotor performance were worse when associated to sleepiness in SAHS than with fatigue in CFS. |

| | | | | | | | | |
|---|---|---|---|---|---|---|---|---|
| *Neu 2014* | Dimensions of pure chronic fatigue: psychophysical, cognitive and biological correlates in the chronic fatigue syndrome | CFS (16) | matched controls (without fatigue) (14) | | U | To investigate associated dimensions of fatigue regarding cognitive impairment, psychomotor performances, muscular effort power and circulating cytokine levels and their relations to symptom intensity in a sample of pure chronic fatigue syndrome (CFS) patients without overlapping objective sleepiness or sleep disorders. | Ppsychomotor vigilance task (PVT); finger tapping test (FTT) (execution motor speed); symbol span or digit symbol substitution test (DSST) (sustained attention, speed of response, and visual scanning); Zazzo's cancellation task (ZCT); | Patients with CFS presented with impaired attention (DSST, ZCT), slower reaction times (PVT) but not with lower hit rates (FTT). |
| *Ocon 2012* | Increasing orthostatic stress impairs neurocognitive functioning in chronic fatigue syndrome with postural tachycardia syndrome | CFS/POTS (16) | control subjects (20) | | C | To investigate whether orthostatic stress causes neurocognitive impairment in CFS/POTS related to decreased CBFV (cerebral blood flow velocity). Participants underwent graded tilt table testing with continuous cardiovascular, cerebrovascular, and respiratory monitoring and neurocognitive testing using an n-back task at each angle. | The n-back task tests working memory, concentration, attention and information processing. | There were no n-back accuracy or RT (reaction time) differences between groups while supine. CFS/POTS subjects responded less correctly during the n-back task test and had slower normalized Rs) at 45, 60 and $75^{\circ}$. Furthermore, at $75^{\circ}$ CFS/POTS subjects responded less correctly and had greater nRT than controls during the 2-, 3- and 4-back tests. Changes in CBFV were not different between the groups and were not associated with n-back task test scores. Thus it was concluded that increasing orthostatic stress combined with a cognitive challenge impairs the neurocognitive abilities of working memory, accuracy and information processing in CFS/POTS, but that this is not related to changes in CBFV |

| | | | | | | | | |
|---|---|---|---|---|---|---|---|---|
| *Petrie 1995* | The impact of catastophic beliefs on functioning in chronic fatigue syndrome | CFS (282) | | | | To investigate the association between catastrophic beliefs and disability in the context of Chronic fatigue syndrome (CFS). | | While not differing on the length of illness or psychological adjustment, subjects demonstrating catastrophic responses evidenced significantly higher levels of fatigue and were more disabled in terms of their ability to work both in their normal occupation and around the house. Catastrophizers also showed greater disability in terms of their sleep and rest, social communication, and recreational activities. |
| *Prasher 1990* | Sensory and cognitive event-related potentials in myalgic encephalomyelitis | myalgic encephalitis - VP1 antigen (12) | HC + enteroviral VP1 antigen (25); HC (25) | | C | To investigate objective evidence of cognitive dysfunction with multi-modality sensory evoked potentials and auditory event-related cognitive potentials in a group of ME patients both with and without the enteroviral antigen, VP1 test positive. | | In contrast to the sensory potentials significant differences in the mean latencies of the cognitive potential N2 and P3 were found. Reaction times were also significantly prolonged but the performance in terms of error was not significantly affected. No significant difference emerged in any of the parameters for the VP1 test. P3 was abnormal in latency or amplitude in 36% of the VP1 positive patients for the frequency discrimination task and 48% for the more difficult duration discrimination task. The abnormalities indicate attentional deficits in some patients and slower speed of information processing in others. The prolonged latencies observed in these patients have not been observed in patients with depression in many other studies. |
| *Ray 1993* | Quality of attention in chronic fatigue syndrome : Subjective reports of everyday attention and cognitive difficulty, and performance on tasks of focused attention | CFS (24) | HC (24) | | U | To compare patients with CFS and matched controls on two questionnaires which assess subjective difficulties with attention and general cognitive functioning, and on | The Stroop Colour-Word Interference Test; The Embedded Figures Test (EFT) | Patients with CFShshowed greater attentional difficulty and cognitive difficulties. However, albeit patients with CFS showed somewhat more interference (CW-C) on the Stroop test than controls, and they had a higher mean solution time for items on the EFT, these differences were not statistically significant. |

| | | | | | | | | |
|---|---|---|---|---|---|---|---|---|
| | | | | | | two tasks requiring focused attention. | | |
| *Ray 1997* | Coping and other predictors of outcome in chronic fatigue syndrome: a 1-year follow-up | CFS (137) | | | | To investigate outcome predictors in patients with CFS. | | Nearly two thirds reported an improvement on direct ratings of change. In analyses with fatigue and functional impairment at follow-up as the criteria, and controlling for earlier status, poorer outcomes were predicted by illness duration, subjective cognitive difficulty, and somatic symptoms; there was no influence of anxiety, depression, or general emotional distress. Fatigue was also predicted by information-seeking, and impairment by behavioral disengagement and a low internal locus of control. The belief that one's actions can influence outcomes modified the relationship between illness accommodation and both fatigue and impairment; adverse outcomes were associated with accommodating to illness only in the context of lower levels of perceived control. Thus, it is suggested that interventions that either discourage avoidance of activity or enhance perceived control could benefit the course of the illness. |
| *Riccio 1992* | Neuropsychological and psychiatric abnormalities in myalgic encephalomyelitis : A preliminary report | Myalgic encephalitis (9) | HC (9) | | U | To investigate neuropsychological deficits in CFS. | National Adult Reading Test (NART); Wechsler Adult Intelligence Scale - Revised (WAIS-R) (general intellectual functioning); Wechsler Memory Scale (WMS): Logical Memory, Paired Associate Learning, Visual Memory; Semantic Processing Test; Verbal Fluency; Grooved Pegboard Test; Modified | Subjects with CFS showed worse performance on two tests of verbal memory. Their personality scores displayed less extraversion and less psychoticism. This is the first report of objective neuropsychological abnormalities in patients with ME, suggesting a discrete deterioration of short-term memory. The findings may also suggest a concurrent psychiatric component of the condition, but the |

| | | | | | | | | |
|---|---|---|---|---|---|---|---|---|
| | | | | | | | Wisconsin Card Sorting Test; Trailmaking A and B | direction of causality remains to be clarified. |
| *Ross 2001* | Divided Attention Deficits in Patients With Chronic Fatigue Syndrome | CFS (17) | normal controls (19) | | U | To investigate abnormalities focusing, sustaining, or dividing attention in patients with CFS, and to related their cognitive profile with other mood state, personality, and neuropsychological measures. | Continuous Performance Test (CPT) - sustained attention and vigilance; General intellectual functioning; Memory tests - The Wechsler Memory Scale–Revised; Hebb's Recurring Digits test; Corsi blocks and supraspan learning tests; The Recurring Figures Test - visual-spatial recognition; The Rey Auditory–Verbal Learning Test (RAVLT) - immediate and long-term verbal memory and the effects of proactive and retroactive interference; verbal fluency - Newcombe test of verbal fluency, Thurstone word fluency test; | CFS patients demonstrated a selective deficit on 3 measures of divided attention. Their performance on the other neuropsychological tests of intelligence, fluency, and memory was no different than that of normal controls despite their reports of generally diminished cognitive capacity. There was an inverse relation between CFS patient fatigue severity and performance on 1 of the divided attention measures. Given these findings, it is probable that CFS patients will report more cognitive difficulties in real-life situations that cause them to divide their effort or rapidly reallocate cognitive resources between 2 response channels (vision and audition). |
| *Sandman 1993* | Memory Deficits Associated with Chronic Fatigue Immune Dysfunction Syndrome | CFS (39); D (23) | HC (129) | | C (partially) | To examine cognitive deficits in CFS patients. | Performed only by CFS patients - Mini-Mental State Examination (MMSE), WAIS-R (Wechsler 1981), Wechsler Memory Scale-Revised (WMS-R), Wisconsin Card Sort Test (WCST), Trail-Making Test (TMT), Boston Naming Test (BNT) and the Visual-Function scale (C4) of the Luria Nebraska Neuropsychological Battery (LNNB). Tests performed by all participants: Recall subtests including metacognitive estimate, free recall, paired | Although the CFS patients had normal neuropsychological profiles, they significantly overestimated their ability (metamemory), performed significantly worse on tests of recall as context increased (e.g., recognition), made more errors when rehearsal was prevented, and had delayed mental scanning as memory load increased. The overall pattern indicated that CFS patients had a significant memory deficit. |

| | | | | | | | | | |
|---|---|---|---|---|---|---|---|---|---|
| | | | | | | | | associates, recognition, and letter-priming subtest.; proactive inhibition; item recognition; semantic memory test; | |
| *Santamarina-Perez 2011* | Length of Illness Does Not Predict Cognitive Dysfunction in Chronic Fatigue Syndrome | CFS (56) (different durations of disease) | | | | The aim of this study was to examine the evolution of cognitive impairment in CFS. Patients were divided into three groups based on the time since the disease was diagnosed: less than 12 months, from 12 to 48 months, and more than 48 months. | Attention and Concentration - Digit Span on the Wechsler Adult Intelligence Scale-Third Edition, Mental Control on the Wechsler Memory Scale-Third Edition, Symbol Digit Modalities Test, Paced Auditory Serial Addition Test; Reaction Time: California Computerized Assessment Package; Memory - Rey Auditory Verbal Learning Test and the ROCF (immediate and delayed recall); Psychomotor Speed: Grooved Pegboard; and Executive Functioning: Stroop Test, the Trail-Making Test (TMT), verbal fluency test, and Tower of London Test. Premorbid intellectual capacity - vocabulary subtest of the WAIS-III. (Wechsler, 1999). | There were no differences between groups in terms of cognitive function. The cognitive impairment in CFS was not found to be more severe with longer disease duration. These data suggest that there is no progressive cognitive impairment in patients with CFS. |

| | | | | | | | | |
|---|---|---|---|---|---|---|---|---|
| *Scheffers 1992* | Attention and short-term memory in chronic fatigue syndrome patients: An event-related potential analysis | CFS (13) | HC (13) | | C | To evaluate attention and short-term memory in CFS patients. Event-related brain potentials (ERPs) were recorded. | To assess attentional and memory deficits in CFS patients, an Attention paradigm was used. Both groups also performed a visual Oddball paradigm, with an RT, before and after the Attention paradigm. Other tests included the Wechsler Adult Intelligence Scale-Revised and the Wechsler Memory Scale. | The patients' RTs were much more variable and, in nine of 13 cases, slower than the mean RT of the controls in both paradigms. The patients' memory performance was not significantly different from that of the controls and there were no group differences in the overall amplitude, latency, or scalp distribution of the N1, P2, N2, or P300 components of the ERP in either paradigm. The ERP and performance data from both paradigms suggest that perceptual, attentional, and short-term memory processes were unaffected in CFS patients and that the differences were limited to response-related processes. |
| *Schmaling 1994* | Cognitive Functioning in Chronic Fatigue Syndrome and Depression: A Preliminary Comparison | CSF (16) | DM (23) | Dot Counting test; Rey´s 15-item test | U | To examine the performance of patients with chronic fatigue syndrome (CFS) and patients in a major depressive episode in order to ascertain Is whether there is objective evidence for impairment in cognitive functioning among patients with CFS, if their cognitive profile dffers from depressed patients and whether depressive symptomatology and subjective levels of fatigue are related with test performance. | Stroop Test; Short Category Test; California Verbal Learning Test (CVLT); Wechsler Memory Scale-Revised (WMS-R) Visual Memory Span and Visual Reproduction subtests; Wechsler Adult Intelligence Scale-Revised (WAIS-R) Vocabulary, Block Design, Digit Span, Digit Symbol subtests; Wisconsin Card Sorting Test; Trail Making Test; Dot Counting Test; and Rey's 15-item test of dissimulation; Estimated IQ scores; | The overall neuropsychological performance of the CFS group was not significantly different from depressed patients, and both groups scored within normal limits on most measures. Variability of neuropsychologic performance was in general unrelated to level of depressive symptoms. |

| | | | | | | | | |
|---|---|---|---|---|---|---|---|---|
| *Schmaling 2003* | Single-Photon Emission Computerized Tomography and Neurocognitive Function in Patients With Chronic Fatigue Syndrome | CFS (15) | HC (15) | | U | To compare functional imaging under control and experimental conditions among patients with CFS and HC and to examine perceived and objective performance on a test of attention and working memory. Single-photon emission computerized tomography scans were completed at rest and when performing the Paced Auditory Serial Addition Test (PASAT). | Paced Auditory Serial Addition Test (PASAT) | No group differences were found for performance on the PASAT despite CFS subjects' perceptions of exerting more mental effort to perform the task. Inspection of the aggregate scans by group and task suggested a pattern of diffuse regional cerebral blood flow among subjects with CFS in comparison with the more focal pattern of regional cerebral blood flow seen among healthy subjects. Between-group region-of-interest analysis revealed that although CFS subjects showed less perfusion in the anterior cingulate region, the change in CFS subjects' activation of the left anterior cingulate region during the PASAT was greater than that observed for healthy subjects. |
| *Schmaling 2015* | Neurocognitive complaints and functional status among patients with chronic fatigue syndrome and fibromyalgia | CFS (50) | CFS/FM (43) | | U | To conduct a longitudinal examination of cognitive complaints and functional status in patients with chronic fatigue syndrome (CFS) alone and those who also had fibromyalgia (CFS/FM). | | Patients with CFS/FM reported significantly worse physical functioning, more bodily pain, and more cognitive difficulties (visuo-perceptual ability and verbal memory) than patients with CFS alone. |

| | | | | | | | | |
|---|---|---|---|---|---|---|---|---|
| *Schrijvers 2009* | Psychomotor functioning in chronic fatigue syndrome and major depressive disorder: A comparative study | CFS (no D) (38) | D (32); HC (38) | | C | To compare fine psychomotor functioning in CFS and D. | Two computerized copying tasks differing in complexity - a line-copying task that mainly required motor effort and a figure-copying task requiring additional cognitive efforts; | Overall, both patient groups performed more slowly than the controls. Compared to CFS patients, patients with MDD needed significantly more time to copy the single lines but no such between-group performance difference was observed for the figure reproductions. In this latter copying task, the increasing complexity of the figures resulted in prolonged reaction times for all three participant groups with the effect being larger and the magnitude similar for the two patient groups. In conclusion, both the MDD and CFS patients tested demonstrated an overall fine motor slowing, with the motor component being more affected in the MDD patients than in the CFS patients while both patient groups showed similar cognitive impairments. |
| *Servatius 1998* | Impaired associative learning in chronic fatigue syndrome | CFS (12) | HC (14) | | C | To measure sensory reactivity and acquisition of the classically conditioned eyeblink response. | | Patients with CFS exhibited normal sensitivity and responsivity to acoustic stimuli. However, CFS patients displayed impaired acquisition of the eyeblink response using a delayed-type conditioning paradigm. Sensitivity and responsivity to the airpuff stimulus were normal. In the absence of sensory/motor abnormalities, impaired acquisition of the classically conditioned eyeblink response indicates an associative deficit. These data suggest organic brain dysfunction within a defined neural substrate in CFS patients. |
| *Short 2002* | Cognitive functioning in Chronic Fatigue Syndrome and the role of depression, anxiety, and fatigue | CFS (23) | HC (23) | | U | This study was designed to investigate the role of depression, anxiety, and fatigue in Chronic Fatigue Syndrome (CFS) sufferers' objective and subjective | WAIS-R; Wechsler Memory Scale—Revised (WMS-R); PASAT; Stroop Colour–Word Interference Test—Victoria Version; Rey–Osterrieth Complex Figure Test (ROCFT) | CFS sufferers did not demonstrate any impairment in objective cognitive functioning compared to the control group, and objective performance was not related to their higher levels of depression or their level of fatigue. Depression scores only accounted for a small amount of the variance in CFS sufferers' lower subjective assessment of |

| | | | | | | | | |
|---|---|---|---|---|---|---|---|---|
| | | | | | | cognitive performance. | | their cognitive performance compared to control participants. There were no differences between the groups on anxiety scores. |
| *Siegel 2006* | Impaired natural immunity, cognitive dysfunction, and physical symptoms in patients with chronic fatigue syndrome: preliminary evidence for a subgroup? | CFS (41) (low or normal NKCA levels) | | | U | This study was designed to examine natural killer cell activity (NKCA) as a potential subgroup marker by comparing the clinical presentations of CFS patients with and without clinically reduced NKCA. | Paced Auditory Serial Addition Task (PASAT); Digit Span subtest of the Wechsler Adult Intelligence Scale—Third Edition. | Relative to CFS patients in the normal-NKCA subgroup, low-NKCA patients reported less vigor, more daytime dysfunction, and more cognitive impairment. In addition, low-NKCA patients performed worse on objective measures of cognitive functioning relative to normal-NKCA patients. |
| *Smith 1993* | Behavioural problems associated with the chronic fatigue syndrome | CFS (57) | HC (19) | | C | To evaluate these behavioural problems using a computerized test battery measuring memory, attention and motor skills. | Psychomotor tasks > Variable fore-period simple reaction time task, Five-choice serial response task, Sustained attention task - detection of repeated numbers; Memory task > Free recall task, Delayed recognition memory task, Digit span task, Logical reasoning task, Semantic processing task, Stroop colour-word test. | The patients reported significantly higher levels of depression, anxiety, physical symptoms and cognitive failures than the controls. Similarly, they reported more negative affect. The patients were slower on psychomotor tasks, showed increased visual sensitivity and impaired attention. Digit span and free recall were not impaired but retrieval from semantic memory and logical reasoning were slower. None of the performance differences between patients and controls could be attributed to differences in psychopathology. |

| | | | | | | | | |
|---|---|---|---|---|---|---|---|---|
| *Smith 1999* | Acute fatigue in chronic fatigue syndrome patients | CFS (67) | HC (126) | | C | To investigate whether patients with CFS show greater impairments on performance as tasks progress and especially if they involve continuous responding. | Variable force-period simple reaction time task; Fixed fore-period simple reaction time task; Five-choice serial response task; Detection of repeated numbers; Logical reasoning task; | CFS patients showed impaired performance compared to the controls and these differences increased as the volunteers developed acute fatigue. In addition, differences between the two groups were larger at the end of the test session. In conclusion this study showed that CFS patients were more susceptible to acute fatigue than healthy controls. |
| *Smith 2003* | Examining the Influence of Biological and Psychological Factors on Cognitive Performance in Chronic Fatigue Syndrome: A Randomized, Double-Blind, Placebo-Controlled, Crossover Study | CFS (36) | | | B | The aim of this study was to determine if neuropsychological deficits in CFS are triggered by exposure to chemicals, or perceptions about the properties of these substances. | Participants were asked to complete neuropsychological tests before and after exposure to placebo and chemical. Four tests were selected in accordance with recommended guidelines for neurotoxicity assessment. Measures used assessed auditory–verbal memory; attention (digit span); and motor speed, coordination, and visuospatial ability (letter cancellation) and Symbol Digit Modalities Test (SDMT). | Results showed decrements in neuropsychological tests scores on three out of four outcome measures when participants rated the substance they had been exposed to as "chemical." No change in performance was found based on actual substance type. These results suggest that cognitive attributions about exposure substances in people with CFS may be associated with worse performance on neuropsychological tasks |
| *Sohl 2008* | Memory for Fatigue in Chronic Fatigue Syndrome: Relationships to Fatigue Variability, Catastrophizing, and Negative Affect | CFS (53) | | | | To evaluate if participants with CFS´s discrepancies between recalled and momentary fatigue are related to catastrophizing, anxiety, and depression and to variability of momentary fatigue and whether catastrophizing, anxiety, and depression would be associated with momentary fatigue. | | Recall discrepancy was significantly related to the variability of momentary fatigue. In addition, catastrophizing, depression, and momentary fatigue were all significantly related to recall discrepancy. Catastrophizing, depression, anxiety, and momentary negative affect were all significantly associated with momentary fatigue. |

| | | | | | | | | |
|---|---|---|---|---|---|---|---|---|
| *Thomas 2009* | An Investigation into the Cognitive Deficits Associated with Chronic Fatigue Syndrome | CFS (307) | HC (126) | | C | To investigate cognitive deficits in patients with Chronic Fatigue Syndrome (CFS). | Free Recall - short-term recall; Variable Fore-Period Simple Reaction Time Task; Repeated Digits Vigilance Task; Distraction from Irrelevant Stimuli (Stroop colour-word interference task) | The reaction time task also provides data indicating that there were significant differences between the reaction times of the CFS and control groups when comparing reaction times at minute intervals. In addition, a greater rate of slowing over the three minutes was seen in the CFS group. The Stroop interference task provided evidence to suggest that distraction by irrelevant stimuli is more pronounced in the patient group. Returning to the vigilance task, the detection rate of repeated digits for each minute was greater in the control group than the patient group and both groups' detection rate decreased over time. Patients recording high scores on the Cognitive Failures Questionnaire performed significantly worse than those recording low scores for the two reaction time tasks. |
| *Tiersky 1998* | Neuropsychological Functioning in Chronic Fatigue Syndrome and Mild Traumatic Brain Injury: A Comparison | CFS (30) | post-concussion (33); HC (20) | | U | To examine complex information processing, attention/ concentration, memory, and higher-order cognitive processes in patients with chronic fatigue syndrome (CFS) and mild traumatic brain injury with postconcussive symptoms (MTBI). | Digit Span subtest of the Wechsler Adult Intelligence Scale-Revised (WAIS-R); Paced Auditory Serial Addition Test (PASAT); Trail Making Test, Parts A and B (Trails A and B); Category Test (CT); California Verbal Learning Test (CVLT); Rey Osterreith Complex Figure (ROCF); Logical Memory subtest of the Wechsler Memory Scale- Revised (WMS-R, LM I and II; immediate and delayed recall). | CFS and MTBI patients demonstrated a similar deficit in complex information processing and verbal learning. Overall, however, individuals who suffered a MTBI demonstrated a greater degree of impairment than did individuals with CFS and healthy controls. |

| | | | | | | | | |
|---|---|---|---|---|---|---|---|---|
| *Tiersky 2001* | Longitudinal Assessment of Neuropsychological Functioning, Psychiatric Status, Functional Disability and Employment Status in Chronic Fatigue Syndrome | CFS (47) | | | | To examine the longitudinal course of subjective and objective neuropsychological functioning, psychological functioning, disability level, and employment status in chronic fatigue syndrome (CFS). | The California Verbal Learning Test (CVLT); Paced Auditory Serial Addition Task (PASAT); Rey–Osterreith Complex Figure Test, Immediate Recall (ROCF–I) and Delayed Recall (ROCF–D) subtests; Wechsler Adult Intelligence Scale–Revised (WAIS–R) Digit Span Forward and WAIS–R Digit Span Backward subtests; | Results indicated that objective and subjective attention abilities, mood, level of fatigue, and disability improve over time in individuals with CFS. Moreover, improvements in these areas were found to be interrelated at follow-up. Finally, psychiatric status, age, and between-test duration were significant predictors of outcome. Overall, the prognosis for CFS appears to be poor, as the majority of participants remained functionally impaired over time and were unemployed at follow-up, despite the noted improvements. |
| *Tiersky 2003* | Functional Status, Neuropsychological Functioning, and Mood in Chronic Fatigue Syndrome (CFS) Relationship to Psychiatric Disorder | CFS (60) | CFS + Psy before (26); CFS + Psy after (21); HC (41) | | U | To examine the role of psychiatric status in reducing health-related quality of life in CFS. | Digit span, block design and vocabulary subtests of the WAIS-R; the Paced Serial Addition Test; the Trail Making Test, parts A and B; California Verbal Learning Test. | On the Paced Serial Addition Test, California Verbal Learning Test (trials 1 to 5; short delay, free recall; and long delay, free recall) and on the digit span (backward) and vocabulary subtests of the WAIS-R, no significant group differences were observed. On the Trail Making Test, the healthy control group and the no-psych group were significantly quicker than the pre-psych group in completing parts A and B. No other differences were found for this measure. Finally, on the digit span (forward) subtest of the WAIS-R, all three groups significantly outperformed the pre-psych group, and no other differences were observed. Thus, overall, when differences in neuropsychological functioning were found to exist, the pre-psych group consistently was the most impaired. |

| | | | | | | | | |
|---|---|---|---|---|---|---|---|---|
| *Togo 2015* | Attention network test: Assessment of cognitive function in chronic fatigue syndrome | CFS (21) | CFS+D (19); HC (29) | Indirect cues - no increase in error rates and prolongued RT only in specific conditions. | C | To investigate attention and information processing in patients with chronic fatigue syndrome (CFS). | Simple reaction time task; Attention Network Task; | Comparison of data from two groups of CFS patients (those with and without comorbid major depressive disorder) to controls consistently showed that error rates did not differ among groups across conditions, but speed of information processing did. Processing time was prolonged in both CFS groups and most significantly affected in response to the most complex task conditions. For simpler tasks, processing time was only prolonged in CFS participants with depression. |
| *Vercoulen 1996* | Prognosis in chronic fatigue syndrome: a prospective study on the natural course | CFS (246) | HC (53) | | | To determine spontaneous improvement after a follow up interval of 18 months in patients with chronic fatigue syndrome and to identify factors that predict improvement. | | Three per cent of patients reported complete recovery and 17% reported improvement. At follow up, there were considerable problems at work and consumption of medication was high. Subjective improvement was confirmed by dimensional change: at follow-up recovered patients had similar scores to healthy subjects and improved patients showed significant improvement on four out of seven outcome measures and had higher scores than healthy subjects in all dimensions. In conclusion, the improvement rate in patients with a relatively long duration of complaints is small. Psychological factors are related to improvement, especially cognitive factors. |
| *Vercoulen 1998* | Evaluating Neuropsychological Impairment in Chronic Fatigue Syndrome | CFS (51) | HC (53) | | C | To evaluate the concordance between impairment found on standardized tests and self-reported neuropsychological problems, and to study the relationship between neuropsychological | Motor speed (CRT baseline movement time); concentration (Trail Making Test, Parts A and B, Symbol Digit total score, and mean time and dispersion width of the cancellation task); attention span (Digit Span total score); verbal memory (California Verbal Learning Test: trial 1, trial 5, total words recalled, list B, and the longterm recall conditions); | A minority of participants were impaired in neuropsychological functioning. There was no relationship between neuropsychological impairment on standardized tests and self-reported memory and concentration problems. Neuropsychological functioning was not related to fatigue or depression. Slowed speed of information processing and motor speed were related to low levels of physical activity. |

| | | | | | | | | |
|---|---|---|---|---|---|---|---|---|
| | | | | | | functioning and fatigue severity and psychological processes. | visual memory (Rey Figure recall score); speed of information processing (CRT baseline reaction time and the difference score between reaction time task 1 vs. task 2). | |
| *Vollmer-Conna 1997* | Cognitive deficits in patients suffering from chronic fatigue syndrome, acute infective illness or depression | CFS (21) | D (21); infections (21); HC (21) | | C | To assess cognitive performance in patients with CFS and compare cognitive performance and subjective workload experience of these patients with that of two disease comparison groups (non-melanchonic depression and acute infection) and healthy controls. | Auditory discrimination task; a pursuit-tracking task; divided attention task; short-term memory task; arithmetical task; a left-right discrimination/spatial orientation task; Mackworth clock, vigilance task; | All patient groups demonstrated increased errors and slower reaction times, and gave higher workload ratings than healthy controls. Patients with CFS and non-melancholic depression had more severe deficits than patients with acute infection. All patient groups reported more severe mood disturbance and fatigue than healthy controls, but patients with CFS and those with acute infection reported lesssevere mood disturbance than patients with depression. As all patients demonstrated similar deficits in attention and response speed, suggesting that common pathophysiological processes are involved. The differences in severity of mood disturbance, however, suggest that the pathophysiological processes in patients with CFS and acute infection are not simply secondary to depressed mood. |

| | | | | | | | | |
|---|---|---|---|---|---|---|---|---|
| *Wallman 2005* | Cognitive variables in chronic fatigue syndrome and the role of graded exercise | CFS (61) | | | C | To assess variability in symptoms and physical capabilities in chronic fatigue syndrome (CFS) participants both before and after a graded exercise intervention. | Modified Stroop Color Word test | Post-intervention scores achieved on the cognitive test improved in both groups from moderately to highly reliable, while postintervention scores for activity levels were similar to baseline values and remained classified as highly reliable. Apart from mental and physical fatigue, baseline ICC scores for all variables assessed were moderately to highly reliable, indicating minimal variability. Baseline scores for mental and physical fatigue were of questionable reliability, indicating a fluctuating nature to these symptoms. Variability in scores for mental fatigue was reduced after graded exercise to an acceptable classification. Results from this study support a variable nature to the symptoms of mental and physical fatigue only. Consequently, in order to more accurately report the nature of mental and physical fatigue in CFS, future studies should consider using repeated-measures analysis when assessing these symptoms. Graded exercise resulted in the reclassification of scores for mental fatigue from questionable to acceptable reliability. |

| | | | | | | | | |
|---|---|---|---|---|---|---|---|---|
| *Wearden 1997* | Cognitive performance and complaints of cognitive impairment in chronic fatigue syndrome (CFS) | CFS no-DEP (24) | CFS Dep (24); HC (18) | | C | To investigate cognitive complains and objective performance in patients with CFS. | Experimental paired associate test; Reading test - Circle Island | CFS subjects were more likely than controls to report that they had concentration problems when reading, that they needed to re-read text and that they failed to take in what they were reading. Subjects then performed a task in which their reading behaviour and text recall was measured. While all CFS subjects complained of general cognitive failures and of difficulties with reading, only depressed CFS subjects recalled significantly less of the text than controls. Severity of complaints about reading problems was not related to amount of text recalled, but was related to severity of depressed mood. However, subjects were able to evaluate accurately their ability to remember the text immediately after reading it and before being tested for recall. Additionally, subjects performed a paired-associate learning task on which no significant differences between the subject groups was found. It is concluded that deficits in cognitive functioning in CFS patients are more likely to be found on naturalistic than on laboratory tasks. |

| | | | | | | | | |
|---|---|---|---|---|---|---|---|---|
| *Wessely 1989* | Fatigue syndromes: a comparison of chronic "postviral" fatigue with neuromuscular and affective disorders | unexplained chronic "postviral" fatigue (47) | | | U | To evaluate cognition problems in patients with chronic fatigue. | | Seventy-two percent of the CFS patients were cases of psychiatric disorder, using criteria that excluded fatigue as a symptom, compared with 36% of the neuromuscular group. There was no difference in subjective complaints of physical fatigue between all groups. Mental fatigue and fatigability was equally common in CFS and affective patients, but only occurred in those neuromuscular patients who were also cases of psychiatric disorder. Overall, the CFS patients more closely resembled the affective than the neuromuscular patients. Attribution of symptoms to physical rather than psychological causes was the principal difference between matched CFS and psychiatric controls. The symptoms of "postviral" fatigue had little ability to discriminate between CFS and affective disorder. The fatigue in CFS appeared central in origin, suggesting it is not primarily a neuromuscular illness. The implications for research and treatment of chronic fatigue are discussed. |
