# Supplementary material for: A unifying theory for cognitive abnormalities in functional neurological disorders, fibromyalgia and chronic fatigue syndrome: systematic review
Source: J Neurol Neurosurg Psychiatry. 2018 May 7;89(12):1308–19. doi: 10.1136/jnnp-2017-317823 (PMC6288708; doi:10.1136/jnnp-2017-317823)
Supplement: Supplementary file 5 [file jnnp-2017-317823supp005.pdf]

FM studies - quality and summary of main findings								
**Study**	**Title**	**FM group (n)**	**Control group (n)**	**Performance validity testing**	**Blinded assessment (B - blinded; U - unblinded; C - computerized)**	**Main objective**	**Neuropsychological tests**	**Main findings**
Arnold 2008	Patient perspectives on the impact of fibromyalgia	women with FM (48)				To assess the impact of FM in patient´s lives (focus groups)		The focus groups conducted with fibromyalgia patients identified symptom domains that had the greatest impact on their quality of life including pain, sleep disturbance, fatigue depression, anxiety, and cognitive impairment. Participants reported that FM affected their cognition, particularly memory and thought processes. Difficilties included not being able to operate at the same levels of mental acuity than they had prior to onset, forgeting important tasks, inability to focus and express themselves clearly, feeling more disorganized, difficulty with planning, inability to respond quickly to a question or when asked to perform a task and driving difficulties. Participants found it difficult to become motivated to begin tasks because the constant presence of pain disrupted patients' concentration and depleted them of energy.
Bennet 2007	An internet survey of 2,596 people with fibromyalgia	FM (2569)			C	To investigate what are the main symptoms of patients with FM (survey)		The most common problems were morning stiffness, fatigue, nonrestorative sleep, pain, concentration, and memory.

Brooks 2012	The relationship between performances on neuropsychological symptom validity testing and the MCMI-III in patients with fibromyalgia	FM (72)		An embedded cognitive symptom validity test: Reliable Digit Span); a stand-alone cognitive symptom validity test - Word Memory Test or Test of Memory Malingering;	C	To assess performance validity in FM.		Of these patients, 21% failed a stand-alone SVT, whereas an additional 15% failed both a stand-alone and embedded SVT. Individuals who failed both stand-alone and embedded cognitive SVTs had higher scores on a number of MCMI-III personality subscales and had elevated scores on MCMI-III modifying indices compared to individuals who passed cognitive SVTs. Moreover, SVT performance was significantly correlated with multiple MCMI-III scores, including modifying indices, as well as the somatoform, depression, and anxiety subscales. In sum, cognitive and psychological symptom validity scores were significantly related
Canovas 2009	Virtual reality tasks disclose spatial memory alterations in fibromyalgia	FM (15)	HC (15)	More errors in virtual reality spatial memory tasks	C	To assess performance on virtual reality spatial memory tasks as well as classical neuropsychological tests in patients with fibromyalgia (FM).	Virtual reality spatial memory tasks - virtual versions of the Morris water maze and the hole board (version called Boxes room); classical neuropsychological tests (general intellect, attention/working memory [digit span backward and Corsi block tapping test backward], visuospatial memory [digit span forward, Corsi block tapping test forward and 10/36 Spatial Recall Test (SRT)].	Patients with FM displayed spatial memory impairments as evidenced by significantly more errors in performing both the Bpxes Rooms and the virtual Morris Water Maze. The fact that there was no difference between the groups in navigating to a visible platform suggests that these spatial memory deficits are not due to differences in understanding the tasks, motivational factors, using the joystick or computer programs. These spatial memory deficits corroborate some of the studies indicating hippocampal or temporal lobe abnormalities in people with FM. In contrast, there were no significant differences between the groups in the neuropsychological tests.
Cherry 2011	Physical Performance as a Predictor of Attention and Processing Speed in Fibromyalgia	FM (51)				To determine whether physical function predicts attention and processing speed in persons with FM, and whether actual physical	Adapted Trail Making Test parts A (TMT-A) and B (TMT-B), Digit Symbol Substitution Test, a composite index of TMT-A, TMT-B, and Digit Symbol Substitution Test combined; physical performance assessments.	Better physical performance was significantly associated with better cognitive performance for the TMT-A (complex attention, executive function) and the composite cognitive score, which included all 3 measures of cognitive function (TMT-A, TMT-B, and DSST) after controlling for age and symptom burden, but only when objective physical performance measures were used to assess physical function.

						performance measures were a better predictor of these cognitive domains than perceived or self-reported physical function.		
Cherry 2012	Positive Associations Between Physical and Cognitive Performance Measures in Fibromyalgia	FM (68)				To investigate the associations between perceived physical function (self-report) and physical and cognitive performance (objective assessments) in persons with FM.	Trail Making Test parts A and B, Digit Symbol Substitution Test, a composite score of these 3 cognitive measures, attention/executive function composite (combined measures of TMT-A, TMT-B, DSF, DSB), processing speed composite (combined measures of Stroop Color, DSST, animal fluency), problem solving (number correct on the everyday problems test), interference/inhibition (Stroop Color-Word minus Color) and episodic memory composite (combined measures of immediate and delayed recall, delayed recognition based on the CERAD word list). Other outcome measures were Composite Physical Function scale, Senior Fitness Test (3 items), Fullerton Advanced Balance scale, 30-foot walk.	Self-report measure of physical function (CPF scale) did not show this relationship with cognitive performance. In the first analysis, three measures from the Senior Fitness battery (lower-body strength, overall functional mobility, aerobic endurance) approached significance in relation to performance on the TMT-B (a measure of attention/ cognitive flexibility) and the composite score (TMT-A and TMT-B [attention], DSST [processing speed] combined). In the second analysis, after controlling for age and FM symptoms, results showed that physical performance measurement scores for multidimensional balance (FAB scale) correlated with attention, processing speed, and problem solving; aerobic endurance (6-minute walk) was associated with attention and inhibition; and gait cadence/velocity correlated with attention and showed a trend for processing speed.

Cherry 2014	Cognitive performance in women aged 50 years and older with and without fibromyalgia	FM (43)	non-FM (44)		U	To evaluate objective cognitive performance as part of a larger study of physical/cognitive function in adults aged 50 years and older with or without a diagnosis of FM.	Mini-Mental State Examination. Consortium to Establish a Registry for Alzheimer's Disease (CERAD) 10-item word list; Stroop Color/Word test; Digit Span Forward and Backward; Trails A, B; Naming animals; Digit Symbol Substitution Coding; Everyday Problems Test;	In a study of women aged 50 and older, FM versus non-FM differences were found on two measures of attention/ executive function (Stroop Color/Word, Digit Span Backward [trend]) as well as processing speed as measured by Digit Symbol Substitution Coding. In contrast, group differences were not found for other aspects of attention/ executive function: working memory (Digit Span Forward), set-shifting/complex sequencing (Trails B), verbal fluency/ monitoring (naming animals), or problem solving (Everyday Problems Test), or an additional measure of processing speed (Trails A); although for most of these tasks, the pattern of performance was still better for non-FM than FM women. Mixed findings across cognitive domains among individuals with or without FM is consistent with the literature and suggest that factors beyond those typically controlled for (e.g., heterogeneity in FM) may be influencing findings
Compan 2011	Cognitive factors in fibromyalgia : the role of self-concept and identity related conflicts	women with FM (30)	control (30)		C	To assess the role of cognitive factors in fibromyalgia by using the patients' personal constructs with the repertory grid technique (RGT).	Kelly's personal construct psychology (PCP) considers human activity as a meaning-making process. According to PCP, individuals create informal theories about the self, other people, their health, and so on. Moreover, their response to events (bodily sensations, interpersonal experiences, professional interventions, etc.) is mediated by this interpretation. The repertory grid technique (RGT), derived from personal construct theory, is a method designed to assess the patient's construction of self and others.	Women with FM had a higher present-self / ideal-self discrepancy (lower correlation is associated with a lower self esteem) and a lower perceived adequacy of others (A negative correlation may indicate that the subject is dissatisfied with the people that surround her, and a high positive correlation might suggest a positive (or even an idealized, if extreme) image of others) and it was more likely to find implicative dilemmas among them compared to controls (implicative dilemmas represent a cognitive structure in which the symptom, represented by the nondesirable pole of one construct, is associated with positive characteristics of the self-identity system in which change is not desire). These dilemmas are a type of cognitive conflict in which the symptom is construed as "enmeshed" with positive characteristics of the self. An improvement in these patients' physical health status could be construed by a substantial proportion of women with fibromyalgia as an undesirable change in their current morally appropriate self-image. Similarly,

								they would tend to view people in their lives who do not have pain as being selfish or with any other undesired moral trait. Thus, not having pain involves, in their system of meanings, becoming another type of person characterized by undesirable moral attributes. Conversely, those who suffer from pain are viewed as good people (e.g., altruistic, responsible, or hardworking.
Coppieters 2015	Cognitive Performance Is Related to Central Sensitization and Health-related Quality of Life in Patients with Chronic Whiplash-Associated Disorders and Fibromyalgia	FM (21)	chronic WAD patients (16); pain-free volunteers (22)		C	To investigate cognitive performance and its relationship with central censitization and health-related quality of life in FM and WAD.	Stroop task (incongruent, non-word, negative priming); Psychomotor vigilance task; Operation span task; measures of central sensitization; health-related QoL	Patientd with FMD and chronic WAD encounter significant cognitive impairment, signs of central sensitization and decreased health-related QoL compared to healthy pain-free individuals. The current study revealed more indices of central sensitization, higher cognitive impairment, and more limitations on health-related QoL in FM patients compared with chronic WAD patients. In particular, FM patients showed higher impairment of self-reported physical health, pressure and deep-tissue hyperalgesia, hampered selective attention, and reduced working memory capacity in comparison with chronic WAD patients. Significant correlations between cognitive impairment and indices of CS and self-reported health-related QoL, respectively, were demonstrated among the 3 study groups. Especially in FM patients cognitive impairment appeared to be related to indices of CS. Reduced selective and sustained attention, as well as reduced working memory were correlated with less TS, so less bottom-up sensitization in FM. However, impaired sustained attention was related to increased deep-tissue hyperalgesia, deficient CPM, and reduced QoL in FM patients.

Cuevas-Toro 2014	Neuropsychological Function, Anxiety, Depression and Pain Impact in Fibromyalgia Patients	FM (85)	HC (85)		C	To analyze executive functioning and decision-making performance, and the relationships between these functions and pain, anxiety, depression and medication in patients with fibromyalgia.	Winsconsin card sorting test (executive functioning); Iowa Gambling task (decision making).	The fibromyalgia group showed normal performance in executive functioning and decision-making. Pain was associated with neuropsychological functioning whereas anxiety, depression and medication were not.
Dick 2002	Attentional functioning in fibromyalgia, rheumatoid arthritis, and musculoskeletal pain patients	FM (20)	Rheumatoid Arthritis (20); Muskuloskeletal pain (20); pain-free controls (20)		U	To investigate whether chronic pain patients have deficits in attentional functioning compared with pain-free controls, and whether fibromyalgia patients have larger deficits in attentional functioning compared with rheumatoid arthritis and musculoskeletal pain patients.	Test of everyday attention (TEA) (including an overall score and summary scores for selective attention, sustained attention, attention switching, and auditory-verbal working memory)	All 3 groups of chronic pain patients, regardless of diagnosis, had impaired cognitive functioning on an ecologically sensitive neuropsychological test of everyday attention. Sixty percent of patients had at least one score in the clinical range of neuropsychological impairment, independent of demography and mood. Fibromyalgia patients were more anxious and somatically aware than rheumatoid arthritis or musculoskeletal pain patients, but did not show larger attentional deficits than other patient groups. This study supports previous findings by reporting that many chronic pain patients have significant attentional dysfunction. An important, novel finding is that regardless of disease status, chronic pain patients demonstrate cognitive impairment when performing everyday attentional tasks when compared with matched pain-free controls.

Dick 2008	Disruption of cognitive function in Fibromyalgia Syndrome	Women with FM (30)	matched controls (30)		U	To explore whether working memory disruption is associated with a decline in performance on attentional stimulus competition tasks in individuals with FMS.	Test of Everyday Attention (TEA) (overall score, auditory verbal working memory, selective attention, sustained attention); Auditory Consonant Trigram (ACT) test; Reading Span Test (RST)	Performance of standardized everyday attentional tasks was impaired in the FMS group compared to controls. Working memory was also found to be impaired in this group. Stimulus interference was found to be significantly worse in the FMS group as the demands of the tasks increased. These effects were found to exist independent of the measures of mood and sleep disruption. However, when pain levels were accounted for statistically, no differences existed between groups on cognitive measures. These findings point to disrupted working memory as a specific mechanism that is disrupted in this population. The results of this study suggest that pain in FMS may play an important role in cognitive disruption.
Duschek 2013	Implicit memory function in fibromyalgia syndrome	FM (18)	HC (25)	Word-steam task	C	To assess implicit memory in FMD patients with a word-stem completion task (WST) paradigm.	Word-stem completion	As a main result, the study revealed markedly lower performance on a word-stem completion task for patients with fibromyalgia than for healthy individuals. This suggests impaired implicit memory function in terms of reduced influence of unconscious priming on the patients' behavior. In other words, the lower performance cannot be ascribed to incomplete effort that has been suspected to produce invalid results in cognitive testing with FMS patients. Instead, the functional aberrances may relate to the patients' primary pain complaints, presumably constituting an effect of inference between central nervous nociceptive activity and cognitive processing
Emad 2008	Hippocampus Dysfunction May Explain Symptoms of Fibromyalgia Syndrome. A Study with Single-Voxel Magnetic Resonance	women with FM (15)	healthy age-matched female controls (10)		C	To investigate dysfunction of hippocampus in patients with fibromyalgia syndrome (FM) using proton magnetic resonance spectroscopy (1H-MRS).	MMSE	The hippocampus was dysfunctional in patients with FM, as shown by lower NAA levels compared to controls, representing neuronal or axonal metabolic dysfunction. As the hippocampus plays crucial roles in maintenance of cognitive functions, sleep regulation, and pain perception, we suggest that metabolic dysfunction of hippocampus may be implicated in the appearance of these symptoms associated with this puzzling syndrom. In our study all patients showed variable degrees of cognitive impairment. Subjective cognitive impairment is a common complaint among patients with FM, the so-called "fibro fog". It was

	Spectroscopy							found that patients perform more poorly on tests of immediate and delayed recall, and their ratings of both their memory abilities and sleep quality were lower than those of controls.
Fayed 2012	Brain dysfunction in fibromyalgia and somatization disorder using proton magnetic resonance spectroscopy: a controlled study	FM (10)	STD (10); age-matched healthy control subjects (10)		U	To evaluate the brain metabolite patterns in patients with fibromyalgia (FM) and somatization disorder (STD) through spectroscopy techniques and correlate these patterns with psychological variables.	MMSE	The results from this study demonstrate a significant increase in the levels of Glx, a combined measure of glutamate (Glu) and glutamine (Gln), within the PCC in FM and, to a lesser extent in Somatization disorder, as compared with controls. This factor also correlates with the PCS and the MMSE, suggesting that elevated levels of Glx in the posterior cingulate are associated with increased pain catastrophizing and cognitive impairment. *** The MMSE scores suggested symptoms of cognitive dysfunction in FM and STD, but at levels less severe than those found in patients with dementia.
Follick 2015	Heterogeneity in fibromyalgia based upon cognitive and physical performance and psychological symptomology	women with FM (57)			U	To investigate the existence of subgroups within a fibromyalgia (FM) sample based on physical and cognitive performance measures, as well as self-report psychological measures.	MMSE; Stroop Color/Word Test; Digit Span Forward and Backward; Digit Symbol Substitution Coding; Everyday Problems Test;	Study results support the existence of subgroups among the FM population based on levels of cognitive and physical performance and psychological symptoms: Cluster I - High Physical Performance, High Cognitive Performance, Low Psychological Symptoms (HHL); Cluster II - Moderate Physical Performance, Moderate Cognitive Performance, Low Psychological Symptoms (MML); Cluster III - Moderate Physical Performance, Moderate Cognitive Performance, High Psychological Symptoms (MMH); Cluster IV - Low Physical Performance, Low Cognitive Performance, High Psychological Symptoms (LLH).

Gervais 2001	Effort testing in patients with fibromyalgia and disability incentives	FM (96)	Rheumatoid arthritis (16)	Computerized Assessment of Response Bias (CARB); Word Memory Test (WMT)	C	To assess performance validity in FM.		A large percentage of patients with FM who were on or seeking disability benefits failed the effort tests. Only 2 patients with FM who were working and/or not claiming disability benefits and no patient with RA scored below the cutoffs for exaggeration of memory difficulties.
Gier 2003	Fear of pain, physical performance, and attentional processes in patients with fibromyalgia	FM (81) - high and low fearful			C	To examine the role of pain-related fear and attentional processes on tolerance for physical activity in fibromyalgia patients.	Reaction-time task and dual task	In fibromyalgia patients, pain-related fear is associated with increased pain, decreased tolerance for physical performance, decreased speed of cognitive performance and increased tender point sensitivity. Together, this may be interpreted as indicating heightened attention to somatosensory signals, leading to more pain, increased tender point sensitivity and increase interference with other attention demanding tasks. The relationship between fear and physical performance may be indirect, namely through increased pain, which serves as an occasion setter to signal that it is unwise to continue physical activity.
Glass 2005	Memory beliefs and function in fibromyalgia patients	FM (23)	age- and education-matched controls (23); older controls (22)		C	To investigate memory beliefs and their relationship to actual memory function in fibromyalgia (FM) patients.	Metamemory in adulthood questionnaire; Free recall scores	Overall, FM patients reported lower memory capacity and less stability in memory function than did the age matched controls or the older controls, despite the fact that the objective memory performance between FM patients and older controls was nearly identical [3]. It is plausible that FM patients are more sensitive to their memory loss because their memory function is age inappropriate and may have declined over a shorter time period than is typical with normal aging. Complaints of decreasing memory capacity seem to be accurate in FM patients because they did have lower memory performance than did the age-matched controls, and their perceived memory capacity was well correlated with objective memory performance.

Glass 2011	Executive Function in Chronic Pain Patients and Healthy Controls: Different Cortical Activation During Response Inhibition in Fibromyalgia	FM (18);	age-matched healthy controls (14)		C	To investigate neural correlates of executive function with functional magnetic resonance imaging (fMRI). performed during a simple Go/No-Go task (response inhibition).	Simple Go/No-Go task (response inhibition).	The groups showed no differences in RTs and/or accuracy in executive functioning assessment. The authors commented that this was not surprising, given that a simple Go/No-Go paradigm was used, and deficits in performance in individuals with FM are typically only seen on very demanding tasks. fMRI analyses, on the other hand, revealed significant differences between the FM and HC groups with a hypo-activation in FM in the premotor cortex, SMA, MCC, putamen, and, after controlling for anxiety, in the right IC. Hyperactivation was seen in the right inferior temporal gurys/fusiform gyrus.
Grace 1999	Concentration and memory deficits in patients with fibromyalgia syndrome	FM (30)	HC (30)		U	To assess cognitive performance in patients with FM.	WMS-R (General Memory Index - including Verbal and Visual Memory Indices; Attention/Concentration Index, Delayed Recall Index); Rey Auditory Verbal Learning Test (RAVLT); Paced Auditory Serial Additions Test (PASAT); Symbol Digit Modalities Test – Written Version;	Results indicated that patients with FM performed more poorly on tests of immediate and delayed recall, and sustained auditory concentration, and their ratings of both their memory abilities and sleep quality were lower than those of controls. Furthermore, perceived memory deficits of the FS subjects were disproportionately greater than their objective deficits. Results indicated significant correlations between performance on memory and concentration measures and scores on questionnaires of pain severity and trait anxiety
Grisart 2002	Controlled processess and automacy in memory funcioning in Fibromyalgia Patients: Relation With Emotional Stress and Hypervigilan	a) FM (16); b) FM (12)	a) chronic localized pain (17), pain-free healthy subjects (20); b) chronic localized pain (11), healthy controls (15)		U	To ascertain the relative contribution of controlled and automatic processes in recall performance among FMD, other chronic pain patients and healthy controls.	Cued recall task	Impairment of controlled recall processes was more pronounced in FM than in chronic pain patients, suggesting a higher attentional cost of pain experience in FM. In contrast, there was a rise in automatic recall processes in FM, as compared with LP and healthy controls. The rise in automatic influences in FM may correspond to an adaptative reaction, motivated by the disturbance of controlled processes. * Psychotropic drugs didn´t show an effect, neither any other isolated factor

	ce							
Ickmans 2015	Associations Between Cognitive Performance and Pain in Chronic Fatigue Syndrome: Comorbidity with Fibromyalgia Does Matter	CFS FM (30)	CFS only (18); healthy and inactive controls (30)		C	To examine whether there is an association between cognitive performance and self-reported as well as experimental pain measurements in CFS patients with and without FM.	Stroop task; psychomotor vigilance task (PVT); operation span (OSPAN) task;	The results underline disease heterogeneity in CFS by indicating that a measure of endogenous pain inhibition might be a significant predictor of cognitive functioning in CFS patients with FM, while self-reported pain appears more appropriate to predict cognitive functioning in CFS patients without FM. *** The analyses revealed that, in patients with CFS+FM, conditioned pain modulation (CPM) is a significant predictor for a higher OSPAN total score (= better working memory capacity) and lower Stroop INT score (= better cognitive inhibition). Self-reported pain (SF-36 bodily pain) seemed neither a significant predictor for cognitive performance in patients with CFS+FM without covariates nor after controlling for age, fatigue, and depression.
Iverson 2007	Test of Memory Malingering (TOMM) scores are not affected by chronic pain or depression in patients with fibromyalgia	FM (54)		TOMM		To assess performance validity in FM.		Despite relatively high levels of self-reported depression, chronic pain, and disability, not a single patient failed the TOMM. In this study, the TOMM was not affected by chronic pain, depression, or both.
Johnson-Greene 2013	Relationship between performance validity testing, disability	FM (85)		Word Memory Test or Test of Memory Malingering; Reliable Digit Span	C	To assess performance validity in FM.		Three groups were formed based on effort testing: Two PVTs Failed, One PVT Failed, and No PVTs Failed. We also formed three groups based on disability status: On Disability, Applying for Disability, and Not on Disability. A total of 37% of the patients failed one or both PVTs. PVT group

	status, and somatic complaints in patients with fibromyalgia							analyses were significant for daily pain, weekly pain, and sleep, but not fatigue.
Jose Rodríguez-Andreu 2009	Cognitive impairment in patients with Fibromyalgi a syndrome as assessed by the Mini-Mental State Examination	FM (46)	Age- and sex-matched controls per diagnosis of neuropathic (NeP) or mixed pain (MP) (92)		U	To assess cognitive impairment in patients with FM.	MMSE	Patients with FM showed a high frequency of cognitive impairment (as indexed by total MMSE score), particularly when compared with known prevalence of this condition in the general population. This cognitive impairment remained higher after adjustment by the presence of anxiety and depressive symptoms.
Katz 2004	The prevalence and clinical impact of reported cognitive difficulties (fibrofog) in patients with rheumatic disease with and without fibromyalgia .	FM (57)	rheumatic disease without FM (57)		B	To asssess cognitive dysfunction prevalence, co-occurrence, and impact on symptom severity in fibromyalgia (FMS) and rheumatic disease without FMS.	Information pertaining to memory decline, mental confusion, and speech difficulty was extracted from questions embedded in a health questionnaire and a blind retrospective chart review.	Compared with the non-FMS sample, patients with FMS complained more often of memory decline, mental confusion, and speech difficulty. Memory decline and mental confusion were coupled more often in patients with FMS. Patients with FMS with this combination of cognitive problems reported more pain, stiffness, fatigue, and disturbed sleep compared with patients with FMS with memory problems alone. Patients with rheumatic disease substantially differ in cognitive vulnerability, with patients with FMS at considerably higher risk for cognitive difficulty. More importantly, the prevalence of a combined disturbance in memory and mental clarity is high and closely associated with the perception of increased illness severity and diminished mental health in FMS.
Kim 2012	Spatial versus verbal memory impairment s in patients with fibromyalgia	women with FM (23)	healthy females (24)		C	To investigate the asymmetrical impairment of cognitive functions between verbal and visuospatial memory and	Digit span task; spatial span task; KAVLT - a Korean version of the Rey auditory verbal learning test; nonverbal memory performance is assessed by the K-complex figure test (KCFT), a Korean version of the RCFT; continuous	Female patients with FM showed poorer performance than normal controls on the long-term visuospatial memory test (KCFT), especially during the encoding and further delayed recall phase, and this impairment was more pronounced than those in other domains of memory, such as short-term memory or longterm verbal memory (KAVLT). These findings suggest that visuospatial memory abilities may be more impaired than

						between short-term and long-term memory.	performance test (CPT); Wisconsin card sorting test (WCST)	verbal memory abilities in patients with FM.
Kratz 2015	Development and Initial Validation of a Brief Self-Report Measure of Cognitive Dysfunction in Fibromyalgia	Study 1: FM (1035); Study 2: FM (232)			C	To devise a 10-item short form measure of cognitive functioning for use in FM.		The MISCI is a 10-item measure of cognitive dysfunction in FM, and proved to be brief but comprehensive measure showing evidence of excellent construct validity through large correlations with a lengthy legacy measure of cognitive functioning.
Landro 1997	Memory functioning in patients with primary fibromyalgia and major depression and healthy controls	FM (25)	D (22); HC (18)		U	The aim was to assess short term and long term memory functioning.	Short-term memory - digit span forward, digit span backward; Long-term memory - Randt Memory Test (General Information; Five Item; Paired Words; Short Story; Picture Recognition task; Incidental Memory); Code Memory Test, Word Fluency task, Kimura Recurring Recognition Figures Test;	

Leavitt 2002	Cognitive and dissociative manifestations in fibromyalgia	FM (89)	rheumatic disease patients (64)		U	To screen participants for memory decline and mental confusion using a questionnaire format.		Cognitive complaints (76.4%–43.8%) and dissociative symptoms (37.1%–1.9%) were overrepresented in patients with FM. Among FM patients with high dissociation, cognitive difficulties were reported by 95%; 100% of these cases reported that both memory and mental clarity were affected, a condition referred to as fibrofog. Dissociation in combination with fibrofog was associated with higher levels of FM symptom intensity and decreased mental well being.
Leavitt 2006	Distraction as a key determinant of impaired memory in patients with fibromyalgia	FM presenting with complaints of memory loss (35)	controls presenting with complaints of memory loss, matched for age and sex (35)		U	To assess the role of distraction in memory impairment in patients with FM.	Participants completed cognitive measures with and without stimulus competition. Auditory Consonant Trigram; WAIS-III; Digit Span backward and forward; Paced Auditory Serial Addition Task (PASAT)	The cognitive costs of distraction appear to be considerable and may be a defining feature of memory vulnerability in patients with fibromyalgia. It was a key parameter along which patients with FM differ. With no distraction on the ACT, immediate memory was largely intact. They fully remembered a small file of information. However, following a distraction of 9 seconds, the loss of information was disproportionately large. This distraction erased almost 58% of the same information, suggesting that even limited distraction harms recall of new information. Indeed, people with FM lost simple information at a rate that was 44% greater than an age matched group presenting with memory problems and almost 3 times greater than the normative sample *** Some insight into why FM patients largely perform psychometrically normally on routine tests of memory, yet are troubled by memory gaps for everyday events, may be gained by highlighting skills that are assessed by memory measures in routine use. Most measures encode information into storage in highly structured, distraction-free situations. These conditions are not representative of difficulties encountered in real life, which is full of stimulus competition that actively interrupts the encoding of new information before it can be rehearsed and stored.

Leavitt 2008	Speed of mental operations in fibromyalgia: a selective naming speed deficit	FM with memory complaints (67)	controls presenting with complaints of memory loss (51)		U	To examine the speed of mental operations in people with the fibromyalgia syndrome (FMS) under the pressure of time. The central question addresses whether FMS is associated with processing speed deficits across a spectrum of speeded tasks.	The WAIS III-R Vocabulary Subtest; Paced Auditory Serial Arithmetic Test; Trail-Making Test Parts A & B; Digits Symbol Substitution Test; The Symbol Search Test; The Stroop Color and Word Test; Symbol Digit Modality Test; Beck Depression Inventory II	The majority of FMS patients (70%) performed within 1 standard deviation of the norm on 7 or more of 10 speeded measures. However, more than 49% of FMS patients tested as impaired (1.67 SD below normative mean) on 2 specific validated speed tasks (reading words and naming colors). Compared with controls, the number of FMS patients showing impairment was 2.0 times greater for reading speed, and 1.6 times greater for color naming speed. *** Abnormalities in naming speed are an unappreciated feature of FMS. Selective deficits in naming speed in association with otherwise well preserved global processing speed set patients with FMS apart from controls with memory complaints.
Leavitt 2009	Normalizing Memory Recall in Fibromyalgia With Rehearsal: A Distraction-Counteracting Effect	FM (91)	control subjects (43)		U	To replicate previous findings relating cognitive impairment to distraction while recalling unrehearsed information in people with FMS, and to determine whether distraction impairs the retention of rehearsed information in a similar fashion.	Four neurocognitive measures free of distraction, along with 2 measures with added distraction, were completed. Differences in the retention of rehearsed and unrehearsed information with a source of distraction present were calculated. NP tests: Logical Memory and Paired Associates subtests of the Wechsler Memory Scale; ACT test; Rey Auditory Verbal Learning Test (RAVLT); Wechsler Adult Intelligence Scale.	Patients with FMS showed normal cognitive functioning on verbal memory tests free of distraction. Adding a source of distraction caused unrefreshed information to be lost at a disproportionate rate in patients with FMS. Over 87% of patients with FMS scored in the impaired range on a task of unrehearsed verbal memory. Adding a source of distraction to well-rehearsed information produced a normal rate of recall in FMS. *** Rehearsal mechanisms are intact in patients with FMS, and play a beneficial role in managing a source of distraction. The retention of repeated information interrupted by a source of distraction is normal in patients with FMS, indicating that distraction does not disrupt memory for well-rehearsed information in patients with FMS. Patients with FMS forget well-rehearsed information at a rate that is very similar to a healthy population following a distraction.

Leavitt 2015	Cross-sectional Neurocognitive Data Do Not Support a Transition From Fibrofog to Alzheimer Disease in Fibromyalgia Patients	a) cohort of FM with a short duration of cognitive problems (≤12 months); b) cohort of FM with a long duration of cognitive problems (≥84 months)			U	To determine if deficits in episodic memory and progressive cognitive decline, hallmarks of Alzheimer's disease, are prominent in the cognitive makeup of fibromyalgia patients.	Stroop Color and Word Test; Controlled Oral Word Association Test, COWAT; The Logical Memory and Paired Associates; Digit Span; The Auditory Consonant Trigram; Paced Auditory Serial Arithmetic Test (PASAT); Digits Symbol Substitution Test; Symbol Search Test; Processing Speed Index; Trail-Making Tests A and B;	Fibromyalgia patients' fear of developing Alzheimer's disease was not borne out by the data. The cognitive pattern of fibromyalgia appears distinct from that of Alzheimer's disease. Fibrofog is not associated with either episodic memory loss on standard tests of episodic memory or progressive cognitive decline. Patients with fibrofog remember personally experienced events termed episodic memory at a normal rate in quiet, distraction-free conditions. At the heart of memory loss in fibromyalgia is the inability to appropriately filter out relevant distractions. Encoding mechanisms that otherwise operate normally in forming episodic memories for everyday events in fibromyalgia appear to malfunction when 2 streams of information operate concurrently (relevant information and a source of distraction overlap). The findings should allay the worries of many with fibromyalgia who fear that fibrofog is the start of a dementing process
Luerding 2008	Working memory performance is correlated with local brain morphology in the medial frontal and anterior cingulate cortex in fibromyalgia patients: structural correlates of pain cognition interaction	FM (20)			U	To determine whether neuropsychological deficits found in FM patients may be correlated with changes in local brain morphology specifically in the frontal, temporal or cingulate cortices.	Revised Wechsler Adult Intelligence Scale; California Verbal Learning Test (CVLT); Rey Visual Design Learning Test (RVDLT); digit span backward; Corsi block span; Trail Making Tests (Parts A and B; TMT A and B)	Performance on non-verbal working memory was positively correlated with grey matter values in the left dorsolateral prefrontal cortex, whereas performance on verbal working memory was positively correlated with grey matter values in the supplementary motor cortex. Pain scores were negatively correlated with grey matter values in the medial frontal gyrus. White matter analyses revealed comparable correlations for verbal working memory and pain scores in the medial frontal and prefrontal cortex and in the anterior cingulate cortex. *** This suggested a dysfunction of the frontal cortex (rather than the temporal/hippocampal regions) which is critically involved in working memory and free recall of memory contents.

Martinsen 2014	Fibromyalgia Patients Had Normal Distraction Related Pain Inhibition but Cognitive Impairment Reflected in Caudate Nucleus and Hippocampus during the Stroop Color Word Test	FM (29)	HC (31)		C	To investigate distraction-induced analgesia, cognitive performance and cerebral activation patterns in FM.	Stroop color word task (SCWT)	In study one, it was found reduced pressure pain sensitivity during SCWT in both groups alike and no statistically significant differences were seen between the incongruent and congruent conditions. The study two revealed longer RTs during the incongruent compared to the congruent condition in both groups. FM patients had longer RTs than HC in both conditions. Furthermore, the authors found a significant interaction between group and congruency; that is, the group differences in RTs were more pronounced during the incongruent condition. This was reflected in a reduced activation of the caudate nucleus, lingual gyrus, temporal areas, and the hippocampus in FM patients compared to HC. In conclusion, there was normal pain inhibition during SWTC in FM patients. The cognitive difficulties seen in FM patients, reflected in longer RTs, were related to reduced activation of the caudate nucleus and hippocampus during incongruent SCWT, which most likely affected the mechanisms of cognitive learning in FM patients.

Melo 2012	Neuropsychological assessment of cognitive disorders in patients with fibromyalgia, rheumatoid arthritis, and systemic lupus erythematosus	FM (13)	RA (13); SLE (11)		U	To assess the possible existence of cognitive disorder associated with fibromyalgia (FM), rheumatoid arthritis (RA) and lupus (SLE).	Mini- Mental State Examination (MMSE); digits forward (DF) and digits backwards (DB) subtests, and Logical Memory I (LM-I) and II (LM-II) of the Wechsler Memory Scale (WMS); Similarities subtest (SIM) of the Wechsler Adult Intelligence Scale (WAIS-III);17 Phonemic Verbal Fluency test (VFT-Pho) and Semantic Verbal Fluency test – Animals (VFT-SeAn) and Fruits (VFT-SeFr) categories;18 clock drawing test (CDT);19 Five-Points test (5PT);20 and neuropsychiatric inventory (NPI).21	Patients with FM, on the other hand, had defi cits in the tests assessing operational memory (LM-I, VFT-SeAn, SIM, and DB), as well as perseverance errors in 5PT, which also refer to executive function disorders. Low educational level and advanced age were associated with various degrees of impairment in the different cognitive functions in the three pathological groups. FM and SLE groups showed significantly higher means of the neuropsychiatric symptoms of anxiety, irritability and hallucinations than the RA group in the neuropsychiatric inventory.
Mercado 2013	Brain correlates of cognitive inhibition in fibromyalgia: Emotional intrusion of symptom-related words	FM (25)	HC (25)		C	To characterize cognitive inhibition mechanisms, as part of the attentional control functions, in patients with fibromyalgia.	Emotional Stroop task	Symptom-related words elicited greater frontal P450 amplitudes and enhanced activation within right inferior frontal gyrus as compared to the rest of stimuli. This effect was only true for the fibromyalgia group. Behavioral contrasts, however, did not produce significant differences. Scalp and source estimation findings suggest the presence of a specific difficulty in cognitive inhibition in fibromyalgia patients (under conditions intimately linked with the core concerns of their disease). Data point to the involvement of right inferior frontal cortices in this inefficient mechanism, which might cause an enhanced and dysfunctional effort of processing to achieve only a comparable

								performance to healthy people. Implications of these results are discussed.
Miro 2011	Attentional deficits in fibromyalgia and its relationships with pain, emotional distress and sleep dysfunction complaints	women with FM (33)	HC (28)		C	To explore whether the three attentional networks (alertness, orienting and executive control) are affected in FM patients as compared to a matched control groups, and to ascertain if these impairments are related to psychological variables such as pain, depression, anxiety and sleep dysfunction.	ANT-I (alertness, orienting and executive control functions of attention)	Results showed that FM patients have impaired executive control (greater interference), reduced vigilance (slower overall reaction time) and greater alertness (higher reduction in errors after a warning cue). With regards to the other attentional networks, no effect was observed in orienting.

Miro 2015	Men and women with fibromyalgia : Relation between attentional function and clinical symptoms	woman with FM (58); men with FM (20)	healthy controls women (21); healthy controls men (27); aged between 30 and 60 years old		C	To explore whether individuals with fibromyalgia (FM) have different attention alterations (i.e., in alertness, orienting, and executive control) depending on their sex.	Attentional function was assessed with the ANT-I task (Attentional Network Test-Interactions).	Patients with FM had impairments in vigilance, alertness, and executive control compared with healthy controls. This attentional alteration was similar in FM men and women. Attention deficit was associated with worse daily functioning in FM women but not in FM men. Emotional distress and sleep disruption may be linked to these cognitive findings, although relationships differed according to sex. The findings suggest that therapy strategies aimed at reducing emotional distress can improve attention to a greater extent in women than men. Srategies aimed at improving sleep quality might have a positive effect mainly on the vigilance/alertness deficit in women and on executive control in men.
Park 2001	Cognitive function in fibromyalgia patients	FM (23)	age- and education-matched controls (n = 23); education-matched older controls who were individually matched to be 20 years older (63 years) than the FM patients (n = 22).		C	To evaluate fibromyalgia (FM) patients for the presence of cognitive deficits and to test the hypothesis that abnormalities would fit a model of cognitive aging.	Speed of information processing, working memory function, free recall, recognition memory, verbal fluency, and vocabulary. Performance on cognitive tasks was correlated with FM symptoms, including depression, anxiety, pain, and fatigue. It was also determined whether memory complaints were correlated with cognitive performance.	The major findings from this study are as follows. First, FM patients performed more poorly on most cognitive measures compared with age- and education matched controls, although they did evidence intact speed of information processing. Second, FM patients performed no differently from healthy adults 20 years. older than their chronological age on most cognitive tasks, except that FM patients had a faster rate of information processing and poorer verbal knowledge than older adults. Third, within the FM group, only self-reported pain on the AIMS predicted poor cognitive performance. Measures of depression, anxiety, and the MPQ scores were all unrelated to poor cognitive performance in FM patients. Finally, cognitive complaints in FM patients were significantly correlated with poorer memory performance.

Paso 2012	Cognitive impairment in fibromyalgia syndrome: The impact of cardiovascular regulation, pain, emotional disorders and medication	FM (35);	HC (29) matched		C	To investigate cognitive performance in fibromyalgia syndrome (FMS) and its association with cardiovascular and clinical parameters.	Uchida–Kraepelin test - a mental calculation task measuring attention and arithmetic processing	The present study revealed reduced cognitive performance in FMS patients in terms of a lower number of arithmetic operations performed in a limited time. In the Uchida–Kraepelin test the number of calculations represents mental speed in comprehensive cognitive functioning, including the storage, manipulation and temporal actualization of information. Results thereby corroborate previous findings of reduced processing speed. However, study groups did not differ with respect to the number of errors on the task. This parameter is supposed to reflect the ability to maintain attention across task execution, thereby predominantly relating to lower order cognitive processing. Thus, in accordance with previous findings, the pattern of results points towards FMS-related deficits in working memory-executive processes, while more automatic processing is largely unaffected. * While the experience of chronic pain was associated with the deficits, co-morbid depression, anxiety, fatigue and sleep complaints appeared to play only a subordinate role.
Schmaling 2015	Neurocognitive complaints and functional status among patients with chronic fatigue syndrome and fibromyalgia	CFS/FM (43)	CFS (50)		U	To examine cognitive complaints and functional status among patients with CFS alone and CFS/FM, and the association of cognitive complaints with functional status over an 18-month longitudinal study.		Patients with CFS/FM reported significantly worse physical functioning, more bodily pain, and more cognitive difficulties (visuo-perceptual ability and verbal memory) than patients with CFS alone. Over time, bodily pain decreased only for participants with CFS alone. Verbal memory problems were associated with more bodily pain for both patient groups, whereas visuo-perceptual problems were associated with worse functional status for patients with CFS alone.

Seo 2012	Working Memory Impairment in Fibromyalgia Patients Associated with Altered Frontoparietal Memory Network	FM (19)	HC (22)		U	The goal was to elucidate the differences in neural correlates of working memory between FM patients and healthy subjects, using fMRI.	n-back memory task	Between-group analyses, after controlling for depression and anxiety level, revealed that within the working memory network, the inferior parietal cortex was strongly associated with the mild and moderate pain ratings. In addition, between-group comparison revealed that within the working memory network, the left DLPFC, right VLPFC, and right inferior parietal cortex were associated with the rating of depression and anxiety. The results suggest that the working memory deficit found in FM patients may be attributable to differences in neural activation of the frontoparietal memory network and may result from both pain itself and depression and anxiety associated with pain.
Shmygalev 2014	Assessing Cognitive and Psychomotor Performance in Patients with Fibromyalgia Syndrome	women with FM (43)	historical control group of healthy volunteers (129)		C	To assess the impact of FM on tests that predict driving ability, assessing visual orientation, concentration, attention, vigilance, motor coordination, performance under stress, and reaction time.	Reaction Time Under Pressure (Determination Test); Attention Test (Cognitrone Test); Test for Motor Coordination (2-Hand); Vigilance Test.	The results indicated that the patients' psychomotor and cognitive performances were significantly non-inferior when compared to healthy controls, with the exception of motor coordination. However, variables such as younger age, depression, anxiety, fatigue, pain, and poor motor coordination, likely contribute to the subjective perception of cognitive dysfunction in FM.
Suhr 2003	Neuropsychological impairment in fibromyalgia: relation to depression, fatigue, and pain	FM (28)	chronic pain patients (27); healthy controls (21)	AVLT	U	To assess the relationship of depression, pain, and fatigue to subjective cognitive complaints and objective impairment in patients with FM.	General intellect - Block Design, Wechsler Adult Intelligence Scale—III; Executive functioning - Wisconsin Card Sorting Test, interference subtest of the Stroop Color and Word Test; Visual and verbal memory - Auditory Verbal Learning Test immediate and delayed recall and delayed recognition and the Complex Figure Test delayed recall;	FM patients had more memory complaints and reported more fatigue, pain, and depression than other groups. Groups were not different in cognitive performance, after controlling for fatigue, pain, and depression; depression was related to memory performance and fatigue was related to psychomotor speed. Neuropsychological test results did not add significantly to the variance accounted for in subjective cognitive complaints, after accounting for depression, pain, and fatigue. In conclusion psychological factors, particularly effort, depression, and fatigue, are important in understanding both subjective cognitive

							Attention/working memory - Arithmetic, Digit Span, and Letter Number Sequencing age-corrected scaled scores from the WAIS-III, and the Paced Auditory Serial Addition Test; complex psychomotor speed - Digit Symbol and Symbol Search scores from the WAIS-III, Controlled Oral Word Association number of words, and Trailmaking Test Parts A and B.	complaints and objective cognitive impairment in FM and other chronic pain disorders.
Tella 2015	Theory of Mind and Emotional Functioning in Fibromyalgia Syndrome: An Investigation of the Relationship between Social Cognition and Executive Function	women with FM (40)	healthy women matched for education and age (41)		U	To investigate a large set of social-cognitive abilities, and the possible relationships between these abilities and the performance on executive function tasks in patients with FM.	Tests for short-term memory (Digit Span-Forward), learning (Rey auditory-verbal learning test), and attention (Trail-making test); Executive functions - Digit Span-Backward, TMT B, Tower of London, verbal fluency. Social cognition - Twenty-Item Toronto Alexithymia Scale (TAS-20); empaty quotient; Ekman 60 faces; Reading the Mind in the Eyes test.	Patients with FM have impairments both in the regulation of their own affect and in the recognition of other's emotions, as well as in representing other people's mental states. No significant correlations were found between social cognition tasks and the subcomponents of the executive function that were analysed.<br>The results showed the presence of several impairments in social cognition skills in patients with FM, which are largely independent of both executive function deficits and symptoms of psychological distress. The impairments reported highlight the importance of adequately assessing ToM and emotional functioning in clinical practice.
Tesio 2015	Are Fibromyalgia Patients Cognitively Impaired? Objective and Subjective Neuropsychological Evidence	FM (30)	HC (30)		C	To assess the neuropsychological performance of FM patients.	Executive function: inhibition - Tower of London; shifting - trail-making test; updating (DS-B and F); access (verbal fluency); Memory: RAVLT; Working memory: n-back paradigm	The results confirmed the presence of impairments of attention, long-term memory, working memory, and shifting and updating executive functions in FM patients compared with healthy controls. These impairments were reflected in patient reports independently of depressive symptoms.

Veldhuijzen 2012	Intact Cognitive Inhibition in Patients With Fibromyalgia but Evidence of Declined Processing Speed	FM (35)	HC (35) - age matched		C	The aim of the present study was to examine performance on cognitive inhibitory tests, operationalized as interference control, in fibromyalgia patients.	To investigate performance on 2 cognitive inhibition tests, the Stroop Color-Word Test (SCWT) and the Multi-Source Interference Test (MSIT).	Significant group differences were found for Stroop Color-Word Test (SCWT) and Multi-Source Interference Test (MSIT) performance in both the neutral (N) and interference (I) conditions with slower reaction times in patients versus controls. However, no significant group differences were found for the difference (I–N) or proportion (I/N) scores, or on the number of errors made. Experimental pressure pain thresholds correlated significantly to several indices of cognition. Psychosocial variables were not related to cognitive test performance. Fibromyalgia patients performed worse on both cognitive inhibition test but to a similar extent for the neutral condition and the interference condition, indicating that there is no specific problem in cognitive inhibition. Evidence of decreased mental processing and/or psychomotor speed was found in patients with fibromyalgia.

Verdejo-Garcia 2009	Executive function and decision-making in women with fibromyalgia	women FM (36)	healthy women (36)-matched in age education and socio-economic status		C	The aim was to examine possible impairment of executive function and decision making in FM.	Performance of in F and HC were compared for two measures of executive functioning: the Wisconsin Card Sorting Test (WCST), which assesses cognitive flexibility skills, and the Iowa Gambling Tasks (IGT; original and variant versions), which assess emotion-based decision-making.<br>They also examinated the relationship between executive function performance and pain experience, and between executive function and personality traits of novelty-seeking, harm avoidance, reward dependence, and persistence (measured by the Temperament and Character Inventory-Revised).	Results showed that on the WCST, FM women showed poorer performance than healthy comparison women on the number of categories and non-perseverative errors, but not on perseverative errors. FM patients also showed altered learning curve in the original IGT (where reward is immediate and punishment is delayed), suggesting compromised emotion-based decision-making; but not in the variant IGT (where punishment is immediate but reward is delayed), suggesting hypersensitivity to reward. Personality variables were very mildly associated with cognitive performance in FM women.
Walitt 2008	Automated neuropsychiatric measurements of information processing in fibromyalgia	FM (27)	control (27); MSK (18)	Estimated based on accuracy on ANAM	C	To compare objective cognitive function between FM and healthy controls at baseline and to determine if symptomatic improvement at follow-up was related to	Automated neuropsychological assessment metrics (ANAM) was used to quantify neurocognitive function. This study used seven of the subtests available in ANAM: Code substitution delay (CDD): cued visual short-term memory; Code substitution (CDS): visual scanning and learning; Matching grids (MTG):	No differences were noted on ANAM between controls and subjects with pain disorders. A clinical improvement was noted with treatment without concomitant change in ANAM scores. No cognitive impairment in FM was demonstrated using ANAM. FM and MSK pain groups performed worse than controls in Stroop color-word test.

						objective cognitive improvement.	spatial processing eficiency; Match to sample (MSP): forced choice short term working memory; Math processing (MTH): eficiency in arithmetic processing; Continual performance (CPT): working memory and sustained attention; Simple reaction time (SRT): simple reaction time. In addition trail making test (Trails A/B) and the Stroop color and word test (Stroop) were also included.		
