# Supplementary material for: A unifying theory for cognitive abnormalities in functional neurological disorders, fibromyalgia and chronic fatigue syndrome: systematic review
Source: J Neurol Neurosurg Psychiatry. 2018 May 7;89(12):1308–19. doi: 10.1136/jnnp-2017-317823 (PMC6288708; doi:10.1136/jnnp-2017-317823)
Supplement: Supplementary file 6 [file jnnp-2017-317823supp006.pdf]

FND studies - quality and summary of main findings								
**Study**	**Title**	**Type of FND (n)**	**Control group (n)**	**Performance validity testing**	**Blinded assessment (B - blinded; U - unblinded; C - computerized)**	**Main objective**	**Neuropsychological tests**	**Main findings**
Bakvis 2009	Basal cortisol is positively correlated to threat vigilance in patients with psychogenic nonepileptic seizures	NEA (19)	ES (17); HC (20) (matched)	No	C	To assess if cortisol levels are positively correlated with enhanced atentional bias scores for angry faces in patients with NEA;	Threat vigilance (Emotional Stroop test);	NEA patients showed a significant attentional bias for angry faces, when compared with ES and HC. Patients with NEA were the only to show a positive correlation between baseline cortisol levels and attentional bias scores for threat stimuli (angry faces).
Bakvis 2009	Trauma, stress, and preconscious threat processing in patients with psychogenic nonepileptic seizures	NEA (19)	HC (20)	No	C	To examine the attentional processing of social threat in NEA in relation to interpersonal trauma and acute psychological stress;	Masked emotional Stroop test (main outcome was the attentional bias score);	No group differences related to the acute stress induction were found. Compared to controls, however, patients displayed a positive attentional bias for masked angry faces at baseline, which was correlated to self-reported sexual trauma. Moreover, NEA patients showed lower HRV at baseline and during recovery. These findings were considered to reflect a state of hypervigilance in patients with NEA, linked with self-reported sexual trauma.

Bakvis 2010	The effect of stress induction on working memory in patients with psychogenic nonepileptic seizures	NEA (19)	HC (20) (matched)	Amsterdam Short-Term Memory test	C	To test whether WM performance in patients with NEA would be more negatively affected by social threat distracter pictures compared with healthy controls (HCs).	For working memory: a N-back task was combined with emotional distracters (neutral, happy and angry faces or no distracter), and performed at baseline and after physiological stress induction with a cold pressor test;	Emotional distracters impaired patients' working memory, both at baseline and after stress induction. Although patients' working memory performance without distractors was unimpaired at baseline, a significant group difference emerged after stress induction. Contrary to NEA patients, HC´s performance improved after stress induction. NEA patients with high cortisol stress responses showed larger stress-induced working memory impairments in the no-distracter condition.
Bakvis 2011	Automatic avoidance tendencies in patients with Psychogenic Non Epileptic Seizures	NEA (12)	HC (20) (matched)	No	C	To test automatic threat avoidance tendencies in relation to stress and cortisol levels in patients with NEA and healthy controls (HCs).	Approach and avoidance (AA) task at baseline and following stress-induction Cold-Pressure Test (CPT);	Patients, but not HCs, showed increased approach-avoidance congruency-effects for angry faces on the AA task at baseline, with relatively slower approach of angry faces, which was overall associated with basal pre-task cortisol. This congruency-effect disappeared after the CPT.<br>The authors commented that these findings provide an objective confirmation of previous suggestions from self-report studies indicating that NEA patients show relatively increased avoidance tendencies to social threat cues.

Binder 1994	Psychological Correlates of Psychogenic Seizures	NEA (19) 19	ES (34)	Portland Digit Recognition Test (PDRT)	U	To assess the neuropsychological correlate of NEA.	MMPI or MMPI-2, Face-Hand Test and Finger Agnosia evaluation;	MMPl correctly identified 71% of the PS group and 65% of the ES group for an overall accuracy rate of 67%. Of the four psychometric measures, the MMPI was the most sensitive to PS and the most accurate overall. Performance on Face-Hand test fell short of significance. NEA made more errors on Finger Agnosia evaluation.<br>PVT: Although NEA scored significantly lower on PDRT, only one NEA patient performed significantly below chance on the PDRT; two other patients were considered to have clinical features suggestive of malingering.
Binder 1998	Neuropsychologic Impairment in Patients with Nonepileptic Seizures	NEA (30)	ES (42), HC (47)	Portland Digit Recognition Test (PDRT)	U	To compare patients with either NEA or ES to normal control subjects on a battery of NP measures.	Part 1 - Assessed in all groups: intelligence coefficient (IQ), Wisconsin card sorting test (WCST) Categories and Perseverative Responses, TMT A and B, finger tapping, grooved pegboard, finger agnosia errors and finger graphesthesia errors;<br>Part 2 - Assessed in NEA and ES groups: WRAT-R Reading Standard Score, Boston Naming, COWAT, Digits Forward, Digits Backward,	Part 1: There were no significant differences between the ES and NEA groups on any variable. HC performed significantly better than NEA subjects on all variables at the 0.01 level except Verbal IQ (p=0.03), WCST categories (p= 0.02) and WCST perseverative responses (p =0.37).<br>Part 2: ES and NEA groups showed no significant differences except for a trend towards worse performances in ES group on Boston Naming Test (p=0.02) and Logical Memory savings percentage (p= 0.09).<br>PVT: PDRT was significantly below chance in only one patient with NEA and below the cut-offs for motivational impairment in two additional NEA patients.

							Rey AVLT Trial 1, Rey AVLT Trial 5, Rey AVLT Trials 1–5, Total, Rey AVLT Brief Delayed Recall, Rey AVLT 20 Minute Delayed Recall, Rey AVLT Recognition, Right Minus Wrong, Rey Complex Figure Copy, Rey Complex Figure Delayed Recall, WMS-R Logical Memory I percentile, WMS-R Logical Memory II percentile, WMS-R LM Savings percentage, CVMT Total Correct and Face Hand Errors;	
Black 2010	The effect of seizures on working memory and executive functioning performance	NEA (216)	ES (207) (TLE)	No	U	To assess whether duration of seizure disorder and lifetime seizure load are associated with deficits in higher cognitive functions in patients with temporal lobe epilepsy (TLE) or psychogenic nonepileptic seizures (PNES) .	Working memory deviation quotient, verbal/conceptual deviation quotient and perceptual/ constructional deviation quotient fro3m WAIS-III or WAIS-R, Trails A, Trails B, Color/Word interference from the Stroop Task, number of perseverative errors and number of categories achieved on the Wisconsin Card Sort Test and number of words	In the NEA group, age at onset and prospective lifetime seizure (contrary to duration) were predictors of neuropsychological performance. This suggests that the earlier the onset of events and the more frequently the events occur, the more influence NEA will have on higher cognitive functioning. TMT-A and TMT-B were affected negatively by one or both of the predictors. As Age at Onset increased, the number of categories achieved on the WCST decreased. The authors hypothesized that the fact that age at event onset and prospective lifetime seizure load had a significant negative effect on neuropsychological

							generated on a verbal fluency test;	performance may also be explained by poor health perceptions.
Bortz 1995	Differential response characteristics in nonepileptic and epileptic seizure patients on a test of verbal learning and memory	NEA (18)	ES (23) [TLE; 12 with left temporal foci (LT), 11 with right temporal foci (RT)] (matched for age, sex, education and IQ)	No	U	We examined whether differences in performance in the California Verbal Learning Test allows a distinction between NEA and epilepsy with left or right temporal lobe foci.	California Verbal Learning Test (CVLT);	NEA patients explicitly recognized fewer target words compared with ES patients. In addition, NEA patients rarely made false-positive errors, which resulted in failure to endorse a significant number of items on the recognition list. This response tendency is called a negative response bias.<br>LT patients endorsed a high number of items on the recognition test, which resulted in a positive response bias. RT patients demonstrated no consistent response tendency. The authors proposed a negative response bias might be hekpful to identify patients with NEA and also speculated that it might reflect aspects of psychological denial.

Breier 1998	Quality of life perception in patients with intractable epilepsy or pseudoseizures	NEA (25)	ES (43) (unilateral, LTL or RTL epilepsy) (NEA patients were older and had a shorter disease duration)	No	U	To examine the relationship between self-reported cognitive difficulties and objective measures in NEA and temporal lobe epilepsy.	Verbal selective reminding test, non-verbal selective reminding test, Boston naming test (Language) and digit span subtest of the Wechsler Adult Intelligence Scale-Revised (Attention);	In comparison with NEA, patients with LTLE showed reduced verbal memory and language abilities and patients with RTLE showed reduced non-verbal memory. NEA and ES groups tended to perceive themselves as functioning similarly in the seizure specific and cognitive domains. This is despite de findings that patients with NEA perform at significantly higher levels than patients with CPS on objective language and memory tests. For both NEA and ES, depression correlated significantly with memory, language and attention/concentration scales. In the NEA group there was no relationship between subjective and objective measurements of cognitive status within this group independent of mood disorder. For the ES group, relationship between subjective and objective measures of cognitive function was dependent on the side of seizure onset, and independent of depression.

Cragar 2006	Performance of patients with epilepsy or psychogenic non-epileptic seizures on four measures of effort	NEA (21)	ES (41); NEA+ES (18)	Digit Memory Test (DMT), Letter Memory Test (LMT), Test of Memory Malingering (TOMM), and Portland Digit Recognition Test (PDRT)	C (partially)	To assess performance validity on cognitive testing in patients with NEA and ES.		There were no differences in the mean scores between groups in DMT, LMT, TOMM and PDRT. The proportion of patients below cut-off was not significantly different for LMT, DMT and PDRT. Only for TOMM NEA patients failed significantly more. Across diagnostic groups, 76% of NEA patients, 89% of ES/NEA patients and 78% of ES patients passed all the effort measures. The authors observed that in general the number of patients performing in a manner suggesting poor effort was fairly small.
Demartini 2014	The role of alexithymia in the development of functional motor symptoms (conversion disorder)	FMD (55)	Organic Movement Disorders (OMD) (33); HC (34)	No	C (partially)	The investigate the role of alexithymia in the development of functional motor symptoms	20-item Toronto Alexithymia Scale (TAS-20); Reading the Mind in the Eyes´ test;	Alexithymia was present in 34.5% of patients with FMD, a significantly higher percentage than in OMD (9.1%) and HC (5.9%) even after controlling for the severity of symptoms of depression. The authors hypothesized that inability to identify emotions at a cognitive level might cause some patients to misattribute autonomic symptoms of anxiety, for example tremor, paraesthesiae or paralysis to that of a physical illness.

Dodrill 2008	Do patients with psychogenic nonepileptic seizures produce trustworthy findings on neuropsychological tests?	NEA (32)	ES (65)	WMT	U	To attempt to replicate Drane et al. (2006)´s findings on effort testing in NEA and ES but with a more broadly selected sample of patients.	Wechsler Adult Intelligence Scale-III (WAIS-III), tests from the "Neuropsychological Battery for Epilepsy" (including Stroop Test, Wechsler Memory Scale-III Auditory Memory (Immediate), Wechsler Memory Scale-III Visual Memory (Immediate), Name Writing speed, Finger Tapping Total, Trail Making Test Part B, Aphasia Screening Test).	The essential findings of the "Drane 2006" study could not be replicated.<br>Except for "Finger Tapping" (worse in NEA group), neuropsychological performances were not significantly different for ES and NEA groups.<br>PVT: the proportions of patients with NEA (28%) vs ES (25%) failing on WMT were not significantly different.<br>People who do poorly on any of the tests in this study do poorly on the battery as a whole. The authors consider that it would have been a mistake to look only at the WMT and to conclude that since scores on the WMT are related to test performance overall, the WMT must be “driving” general neuropsychological performance. A unique role for the WMT in measuring test-taking motivation could not be confirmed in this study.<br>The interaction between the effects of group (NEA vs ES) and PVT test performance (passing vs failing) was not significant for any of the neuropsychological tests.

Drake 1992	Neuropsychological and psychiatric correlates of intractable pseudoseizures	20 NEA (all female, and all but one were under 40 years of age; intractable and at times continuous NEA)	Uncontrolled	No	U	To study neuropsychiatric features a sample of patients with intractable psychogenic seizures.	WAIS-R, Wechsler Memory Scale and Halstead-Reitan Battery	Wechsler Adult Intelligence Scale (WAIS-R), Wechsler Memory Scale and Halstead-Reitan Battery suggested cognitive impairment in 13 of 16 non-retarded individuals: bilateral and predominantly frontal dysfunction was indicated in seven, and six patients had evidence of impairment of either right or left hemisphere. Mental retardation was present in four patients.
Drake 1993	Neuropsychological and Event-Related Potential Correlates of Nonepileptic Seizures	NEA (11)	ES (14)	No	U	To investigate neuropsychological and event-related potential correlates of nonepileptic seizures.	Wechsler Adult Intelligence Scale-Revised (WAIS-R);	Patients with NEA had generally higher IQs (although not significantly different) and greater psychopathology on neuropsychological scales (MMPI).<br>Auditory evoked potentials: P160, N200, P300 latencies were significantly longer in epileptics; amplitudes were higher in epileptics, but the difference was not significant;
Drane 2006	Cognitive Impairment Is Not Equal in Patients with Epileptic and Psychogenic Nonepileptic Seizures	NEA (43)	ES (41)	Word Memory Test (WMT)	U	To assess neurocognitive impairment and performance validity on cognitive testing in patients with PNES and ES.	"The Neuropsychological Battery for Epilepsy" (comprising the entire Halstead–Reitan Battery, the Stroop Test, the Seashore Tonal Memory Test and portions of the 3rd edition of the Wechsler Memory Scale).	NEA´s and ES´s group performances on the "The Neuropsychological Battery for Epilepsy" did not differ significantly (Dodrill Discriminatory Indexes were similar)<br>PVT: Patients with NEA failed more frequently on WMT (51.2%) than patients with ES (8.1%) (p = <0.001).<br>Importantly, patients with NEA who passed the WMT produced neurocognitive scores more similar to those expected from HC than to those expected from

								patients with epilepsy. In contrast, patients with NEA who failed performed significantly worse than patients with confirmed epilepsy.
Fargo 2004	Accuracy of self-reported neuropsychological functioning in individuals with epileptic or psychogenic nonepileptic seizures	NEA (37)	ES (45)	No	U	To assess the relationship between self-perceptions of neuropsychological function and actual objective performance among individuals with NEA and ES.	Verbal memory - immediate and delayed portions of the Logical Memory, Verbal Paired Associates, and Word Lists subtests of the Wechsler Memory Scales, Third Edition (WMS-III). Language - Visual Naming Test (VNT) of the Multilingual Aphasia Examination, the Boston Naming Test (BNT), and the Information subtest of the WAIS. Attention and concentration - Digit Span and Arithmetic subtests of the WAIS as well as the Spatial Span subtest of the WMS-III	Subjective ratings of neuropsychological functioning were only partially accurate within each seizure group: 1. Although patients with ES accurately rated their memory performance, they tended to overestimate both language and attention/concentration abilities; 2. Patients with NEA accurately rated attention/ concentration but underestimated memory and overestimated language abilities.<br>In both groups, poorer self-reported neurocognitive functioning was strongly related to poorer mood state. However, mood state did not predict objectively measured neurocognitive abilities

Griffith 2008	Optimism, pessimism, and neuropsychological performance across semiology-based subtypes of psychogenic nonepileptic seizures	NEA (catatonic, minor motor and major motor NEA)	Uncontrolled	No	U	To investigate the relationships between optimism, pessimism, severity of depressive symptoms, and neuropsychological performance across semiology-based subtypes of PNES.	Measures of optimism and pessimism: Revised Optimism–Pessimism Scale (PSM-R); Optimism and Pessimism Scale/Generalized Outcome Expectancies (OPS/GOE). Intelligence: WAIS-3 and WAIS-R FSIQ scores; Verbal memory composite: Auditory Immediate Index and Auditory Delayed Index standard scores of the Wechsler Memory Scale. Language composite: Boston Naming Test (BNT); Controlled Oral Word Association Test (COWAT); Animal Naming total score. Executive functioning composite - Ruff Figural Fluency Test error ratio, Trail Making Test (Part B) completion time, and Wisconsin Card Sorting Test (WCST) - number of perseverative errors.	There were no significant differences in optimism, pessimism, or severity of depressive symptoms across NEA subtypes. However, it was noted a trend toward significance (P = 0.11) in comparing PSM-R Optimism among subtypes, with catatonic more optimistic than the other subtypes. In addition, the catatonic subtype was the only subtype without an elevated MMPI-2 D T score. The catatonic subtype also had a lower frequency of super pessimists than the major motor subtype. There were no significant differences in neuropsychological performance across PNES subtype. However, we noted a trend toward significance in comparing verbal memory across subtype, with catatonic better than the other subtypes.

Gul 2014	Cognitive deficits and emotion regulation strategies in patients with psychogenic nonepileptic seizures: A task-switching study	NEA (72)	HC (72)	No	C	To examine task-switching ability and emotion regulation strategies.	The Emotion Regulation Questionnaire; The task-switching paradigm examines cognitive flexibility in performing various tasks;	NEA showed cognitive impairment in terms of the interrupted ability to switch between emotion and non-emotion face categorizations. In contrast, HC exhibited efficient switching between these face categorizations.<br>Results suggested that patients with NEA used expressive suppression to regulate their emotions more frequently than HC. On the other hand, patients with NEA less frequently reappraised their cognitions than HC. Switching deficits in patients with NEA were positively correlated with expressive suppression but were negatively correlated with cognitive reappraisal.<br>The authors commented that this study demonstrated the presence of switching deficits in terms of inferior cognitive control of emotion in patients with NEA as compared to HC and that the switching deficits are associated with emotion regulation strategies.

Heintz 2013	Neuropsychological profile of psychogenic jerky movement disorders: importance of evaluating non-credible cognitive performance and psychopathology	FMD (26)	GTS (16), HC (22)	No	U	To examine the neuropsychological profile of patients with psychogenic movement disorders.	Attention - Trail-making Test (TMT) parts A, Stroop Task (word reading and color naming); Executive function - TMT part B, Stroop task (color-word interference), category and phonemic fluency tasks; Memory - Rey Auditory Verbal Learning Test (AVLT), Rivermead Behavioral Memory Test, Wechsler Memory Scale (visual reproduction); Reaction time - Vienna Test System; Effort test: Amsterdam Short Term Memory Test (ASTM)	FMD reported more cognitive complaints in daily life.<br>However, FMD only performed worse on a verbal memory task (AVLT immediate reproduction).<br>PVT: Although FMD scored lower in ASTM, the number of subjects below the cut-off was not significantly different.<br>Group differences for AVLT persisted after covarying for ASTM scores.<br>In FMD patients, verbal learning and PVT performance were associated with depression and anxiety complaints.

Hill 2011	Neuropsychological characteristics of nonepileptic seizure semiological subgroups	NEA (173) (phenomoneology: 46 "subjective"; 24 "catatonic"; 48 "minor motor"; 55 "major motor")	ES (180) (TLE)	No	U	To identify neuropsychological characteristics of patients with NEA based on semiological subgroups and to make comparisons among NEA subgroups and with patients with temporal lobe epilepsy (TLE).	Wechsler Abbreviated Scale of Intelligence (WASI), Wechsler Adult Scale of Intelligence-III, Digit Span subtest (WASI-III), Halstead Reitan Trail Making Test, Parts A (Trails A) and B (Trails B), Boston Naming Test (BNT), Brief Visuospatial Memory Test—Revised (BVMT-R) and Rey Auditory–Verbal Learning Test (RAVLT)	General trend for declining neurocognitive performance across NEA subgroups, subjective - catatonic - minor motor - major motor), with increasing percentages of abnormal and impaired scores across the same subgroups.<br>A trend toward significant diferences was noted between the non-motor and motor NEA subgroups on global intelligence, verbal intelligence, and verbal learning.<br>The NEA two-subgroup model (non-motor and motor), in comparison to patients with TLE, showed superior performance by the non-motor NEA subgroup compared with the TLE group on verbal intelligence, confrontation naming, and verbal learning and memory, with a trend toward superior performance across nearly all measures. Conversely, the motor NEA subgroup showed performance generally comparable to that of patients with TLE.

Kemp 2008	The base rate of effort test failure in patients with medically unexplained symptoms	43 Neurological "Medically Unexplained Symptoms" (MUS); non-litigant	39 "Strong simulators"; 35 "Mild simulators "; matched for age, gender and IQ;	Green's Medical Symptom Validity Test (MSVT); Coin-in-the-Hand Test (CIH); Autobiographical Memory Index (AMI); Camden Pictorial Recognition Memory Test; Mental Control Test (MCT);	U	To establish the extent to which patients with medically unexplained symptoms (MUS) presenting to neurology fail effort testing.	IQ - Wechsler Test of Adult Reading (WTAR)	The failure rate (defined as failing two or more effort tests) was 11% in the MUS group, 94% in the mild simulation groups, and 100% in the strong simulation group.<br>None of the MUS patients performed below chance on forced-choice testing, which does not support an explanation of conscious exaggeration among the small minority that failed.<br>The low but significant effort test failure base rate of 11% is open to a variety of interpretations. Non-credible performance on effort tests might be a feature of the psychopathology of patients with MUS. Non-credible performance could then be seen as an extreme and uncommon symptom of somatoform disorder or as a disorder of information processing.<br>The authors discussed that it is also possible, that some patients with somatoform disorder are likely to fail effort testing due to consciously feigning or symptom exaggeration (i.e., factitious disorder or malingering).
Lelliot 1991	Cerebral pathology in pseudoseizures	NEA (20)	NEA + ES (18); NEA + possible ES (5)	No	U	To investigate abnormalities in psychometric testing, electroencephalograms (EEG) and computerised tomography (CT scan) in NEA:	IQ (full scale, verbal and performance); memory function (Weschler logical memory, Benton visual retention and Rey-Osterreith)	Psychometry had been performed on 36 (83%).<br>The average IQ of the whole sample was normal and not significantly different in those with NEA or NEA+ES.<br>Twenty-nine of the sample had had memory tests, and 16 (55%) were judged to have memory deficits, with a similar

								proportion in NEA and NEA+ES groups.
Locke 2006	Relationship of Indicators of Neuropathology, Psychopathology, and Effort to Neuropsychological Results in Patients with Epilepsy or Psychogenic Non-epileptic Seizures	NEA (74)	ES (92); NEA+ES (12); other diagnosis (6)	Test of Memory Malingering (TOMM)	U	To investigate the associations of neuropathology, psychopathology, and effort to neuropsychological test results in a sample of epilepsy and NEA patients.	Intellectual composite: Wechsler Adult Intelligence Scale-III (WAIS-III); Memory composite: Wechsler Memory Scale-III (WMS-III) immediate memory and general memory; Language composite: Boston Naming Test (BNT), Controlled Oral Word Association (COWA), Animal Naming, Wide Range Achievement Test-3 reading subtest (WRAT-3), Verbal comprehension of WAIS-III; Executive functioning composite: Ruff Figural Fluency Test, Wisconsin Card Sorting Test-64 (WCST-64), TMT-B; Ruff 2&7 Selective Attention	NEA scored significantly better than ES in the memory composite. Diagnosis didn´t have a significant impact in any of the other composites.<br>Anti-epileptic drugs had a significant impact on intellectual, memory, language, and motor functioning scores.<br>Longer durations of disorder were associated with poorer memory and language functioning.<br>Psychopathology, as measured by select MMPI-2 clinical scales, was associated with poorer neuropsychological outcomes in both patient groups.<br>Poor effort was a significant predictor of worse intellectual, memory, language, visual-spatial, and motor functioning in both diagnostic groups.

							test (RSAT); Visual-spatial composite: Ruff Figural Fluency Test, Judgment of Line Orientation (JOLO), perceptual-organization index of WAIS-III; Motor composite: Finger Tapping Test, Grooved Pegboard. MMPI-2	
Myers 2014	Cognitive differences between patients who have psychogenic nonepileptic seizures (PNESs) and posttraumatic stress disorder (PTSD) and patients who have PNESs without PTSD	NEA + trauma + PTSD (17)	NEA + trauma - PTSD (29)<br>NEA - trauma - PTSD (17)<br>(All woman)	TOMM	U	To examine the cognitive profile in patients with PNESs, posttraumatic stress disorder (PTSD), history of trauma but no PTSD, and those without a history of trauma.	Delis–Kaplan Executive Function System (D-KEFS) (higher level cognitive functions), Wechsler Memory Scale (WMS-III) (Logical Memory I and Logical Memory II assess immediate and delayed auditory verbal memory), Continuous Visual Memory Test (CVMT), California Verbal Learning Test-II (CVLT-II) (repetition learning, serial position effects, semantic	Patients with NEAs with comorbid PTSD performed significantly worse on episodic verbal memory (narrative memory) and self-reported greater total, verbal, and visual memory impairments. They also had higher substance abuse history and use of psychopharmacological agents compared with patients without PTSD, regardless of a history of trauma.<br>PVT: 14 participants were excluded after being classified by the neuropsychologist as putting forth insufficient effort (malingering) based on Test of Memory Malingering (TOMM)

							organization, intrusions, and proactive interference), Boston Naming Test (BNT) (visual confrontational word retrieval), Memory Complaints Inventory (MCI); Wechsler Abbreviated Scale of Intelligence Full Scale IQ (WASI FSIQ), TSI-2	
O'Brien 2015	Psychiatric and neuropsychological profiles of people with psychogenic nonepileptic seizures	NEA (20)	HC (20) (matched for gender and age)	Medical Symptom Validity Test (MSVT)	C	To examine the neuropsychological and psychiatric profiles of people with psychogenic nonepileptic seizures (PNES).	Cambridge Neuropsychological Test Battery (CANTAB) (comprising attention, visual memory, semantic/verbal memory, decision-making and response control and executive function domains), Wechsler Abbreviated Scale of Intelligence (WASI) and Wechsler Test of Adult Reading (WTAR).	Patients with NEA showed impaired spatial working memory and attention when compared with HC.<br>Moreover, patients with NEA had significantly higher levels of depression, anxiety, dissociation and alexithymia.<br>PVT: One patient failed the MSVT and was excluded from the analysis.

Prigatano 2009	Self-appraisal and objective assessment of cognitive and affective functioning in persons with epileptic and nonepileptic seizures	NES (23)	ES (22) (similar age, educational level, handedness, age at seizure onset and chronicity of seizures and seizure-like events)	No	U	To compare subjective and objective assessments of cognitive and affective functioning of patients admitted with epileptic and psychogenic nonepileptic seizures.	Delayed recall from Rey Auditory Verbal Learning Test (RAVLT), Brief Visuospatial Memory Test—Revised (BVMT-R), BNI Screen for Higher Cerebral Functions (BNIS) Memory subscale [6], the Trail Making Test Part B, the Wechsler Adult Intelligence Scale III (WAIS-III) Digit Span subtest, the Wechsler Abbreviated Scale of Intelligence (WASI) Verbal IQ, the Boston Naming Test (BNT) and the WASI Performance IQ	Patients with NEA reported greater word-finding difficulty than those with ES, but performed better than the patients with ES on the Boston Naming Test, suggesting a tendency to overestimate word-finding difficulty. NEA also performed better than ES patients in WASI Verbal IQ and BNIS Memory Subscale.<br>Patients with NEA and ES did not otherwise differ in subjective ratings of cognitive functions (memory, concentration and directionality) or emotional state (irritability, anxiety and depression).<br>NEA performed similarly to ES in RAVLT delayed recall, BVMT-R delayed recall, WAIS-III Digit Span, Trail Making Test part B<br>NEA group performed worse on a test of affect expression/ perception (BNIS Affect subscale raw score) when compared with ES group.<br>For patients with NEA, only performance on memory tests was significantly correlated with their anxiety level.

Reuber 2002	Evidence of brain abnormality in patients with psychogenic nonepileptic seizures	NEA (206)	NEA+ES (123)	No	U	To investigate brain abnormalities associated with psychogenic nonepileptic seizures (PNES).	Neuropsychological evaluation included frontal motor skills (finger tapping and Luria sequences), psychomotor speed and attention (d2 letter cancellation), symbol counting interference inhibition, verbal and figural memory and written phonemic word fluency. Mehrfachwortschatz Intelligenztest (MWT-B24) for Verbal IQ	Neuropsychological testing was performed in 33 patients with NEA (16.0%) and 72 patients with NEA + ES (58.5%).<br>Of the NEA patients tested, 20 (60.6%) performed 1.5 SD below the norm in at least one part of the examination. Abnormalities were observed in alertness (14), verbal memory (13), figural memory (7), motor skills (7), verbal fluency (5), and IQ (3).<br>Of the NEA + ES patients tested, 65 (90.3%) had abnormal or highly abnormal scores in at least one of the assessed functions [alertness (40), verbal memory (40), figural memory (32), motor skills (36), verbal fluency (31), abstraction (22), and IQ (13)]. The proportion of patients with NPS deficits was greater in the NEA+ES.

Roberts 2012	Emotion in psychogenic nonepileptic seizures: Responses to affective pictures	NEA (18)	PTS-high (18); PTS-low (18) (PTS = Post-traumatic stress)	No	C (partially)	We assess emotional responses to standard affective pictures in psychogenic nonepileptic seizure (PNES).	Participants viewed pleasant, neutral, and unpleasant pictures from the International Affective Picture System.	NEA patients reported more emotional intensity in response to neutral and pleasant pictures than PTS-low and more intensity to neutral pictures than PTS-high.<br>NEA patients showed less positive emotional behavior to pleasant pictures than PTS-high. The groups did not differ in valence ratings (pleasantness/ unpleasantness), negative emotional behavior, cardiac interbeat interval, or RSA reactivity to the pictures. In terms of emotion regulation, NEA patients reported more overall emotion regulation difficulties and showed lower baseline RSA than PTS-low.<br>NEA patients reported more clinical symptoms on all subscales of the SCL-90-R than PTS-low. Patients with NEA and PTS-high did not differ on these emotion regulation or symptom measures, with the important exception that NEA patients reported more somatic symptoms than PTS-high.

Sackellares 1985	Patients with pseudoseizures: Intellectual and cognitive performance	NEA (19)	NEA+ES (18); ES(20) (patients with NEA tended to be older and more educated)	No	B	To assess the cognitive performance of patients with NEA and/or epileptic seizures.	"Halstead-Reitan Neuropsychology Test Battery and Allied Procedures"; WAIS. Tests were grouped into categories: (1) motor function (Finger Oscillation Test and Hand Dynamometer Score); (2) tactual-kinesthetic problem solving (Tactual Performance Test); (3) alertness, focused attention, and memory (Speech Perception Test, Seashore Rhythm Test, and Memory and Location subtests of the Tactual Performance Test); (4) nonverbal abstraction and mental flexibility (Category Test); (5) intelligence (WAIS) and (6) overall performance (Impairment Index)	The NEA group performed significantly better than the NEA+ES group on each of the IQ measures, on the three Tactual Performance Test subscales and on the Seashore Rhythm Test. Marginal differences between were seen for the Speech Test and the Impairment Index. The NEA group performed significantly better than the ES group on all three measures of intellectual function and on all cognitive measures in the Halstead- Reitan Neuropsychology Test Battery. There were no significant differences between the ES+NEA and ES groups All three groups performed normally on motor performance tasks. The NEA group performed significantly better than the NEA+ES group on each of the IQ measures, on the three Tactual Performance Test subscales and on the Seashore Rhythm Test. Marginal differences between were seen for the Speech Test and the Impairment Index. The NEA group performed significantly better than the ES group on all three measures of intellectual function and on all cognitive measures in the Halstead- Reitan Neuropsychology Test Battery. There were no significant differences between the ES+NEA and ES groups All three groups performed normally on motor performance tasks.

Sackellares 1999	Intellectual and neuropsychological features of patients with psychogenic pseudoseizures	NEA (44)	NEA+ES (8) (Normative data)	No	U		Wechsler Adult Intelligence Test-Revised (WAIS-R); Halstead Neuropsychological Test Battery	For the total sample, the WAIS-R IQ scores were quite variable. A striking finding was the high incidence of impaired performance on the Halstead Neuropsychological Test Battery in both the NEA and NEA+ES groups.<br>Considering the neuropsychological variables for which published cut-off scores are available, performance was impaired in more than 50% of the total sample as well as the subgroups (for NEA only - Halstead index, categories, TPT location, Speech Sounds Perception test, Seashore Rhythm, Finger tapping dominant hand were impaired in > 50%; TPT total and memory, TMT-A and TMT-B in <50%). The Halstead Impairment Index, which reflects the overall level of neuropsychological performance, revealed impairment in 63% of the sample.<br>The authors related the significant neuropsychological impairment in this sample with head trauma, as patients reported a high incidence of accidents and physical trauma.

Schönenberg 2015	Theory of mind abilities in patients with psychogenic nonepileptic seizures	NEA (15)	HC (15) (matched for sex, age, and education level)	No	C	To examine basal facial affect recognition as well as higher-order cognitive mind-reading skills in NEA, in order to investigate specific perceptual and cognitive biases in the processing of social affective information.	Self-report questionnaires measured alexithymia and perceived stress vulnerability. Affect perception - computerized movies of models whose facial expressions slowly change from neutral to full-blown emotions. Movie for the Assessment of Social Cognition - video-based test for the evaluation of mind-reading capabilities; Toronto Alexithymia Scale; animated morph task; Movie for the Assessment of Social Cognition (MASC)	NEA patients showed increased alexithymic traits and impaired mentalizing skills, revealing deficits in reasoning about their own and other people's mental states. However, basal facial expression recognition was not compromised. The authors commented that patients with NEA exhibited specifically impaired social–cognitive mind-reading skills, while social–perceptual abilities tended to be normal.
Stewart 1982	Are hysterical seizures more than hysteria? A research diagnostic criteria, DSM-III and Psychometric Analysis	NEA (13)	ES+NEA (13), ES(11)	No	B	To investigate the neuropsychological profile of NEA.	WAIS	NEA group showed higher verbal, performance and full scale IQ, whereas alexythimia was more frequent in ES group

Strutt 2011	A comprehensive neuropsychological profile of women with psychogenic nonepileptic seizures	NEA (33)	ES (25) - LTLE (NEA also compared with age-matched normative sample data)	TOMM	U	To investigate the neuropsychological profile of NEA.	Sattler's IQ Estimate; Digit Span, Arithmetic, Similarities, Letter–Number Sequencing and Working Memory Index from the Wechsler Adult Intelligence Scale (WAIS-III); Spatial Span, Logical Memory I and II (LM-I and LM-II), Verbal Paired Associates I and II (VPA-I and VPA-II), Visual Reproduction I and II (VR-I and VR-II), and Mental Control from the Wechsler Memory Scale (WMS-III); Boston Naming Test (BNT), Letter Fluency (FAS), Semantic Fluency (Animals), and Trail Making Test Parts A (Trails A) and B (Trails B) from the Revised Comprehensive Norms for the Expanded Halstead–Reitan Battery.	NEA group versus age-matched normative sample data - NEA group scored on the "below average range" in measures of attention, working memory, and information processing speed, in two-thirds of language measures, in only 1 executive functioning task and also only in the encoding trials of both visual memory and non-contextual verbal memory. Sattler's IQ Estimate was in the average range.<br>NEA group versus ES (LTLE) group - for measures of verbal memory and language, NEA group means fell within the average to below average range, whereas the ES group was classified within the borderline impaired range. The NEA group scored significantly better on a measure of verbal memory, including memory acquisition and later retention. Among the remaining neuropsychological variables, significant between-group differences were also found for IQ estimates, auditory attention, spatial attention, and set shifting/mental flexibility, with the NEA group scoring lower than their counterparts with ES.<br>The authors concluded that NEA group showed a consistent relative area of weakness in attention and working memory, in addition to below average performance on neuropsychological measures that can be influenced by fluctuating levels of

								attention/concentration.<br>PVT: Suspect effort/motivation was within normal limits for all participants.

Turner 2011	Patients with epilepsy and patients with psychogenic non-epileptic seizures: Video-EEG, clinical and neuropsychological evaluation	NEA (22)	PNES+ES (10); ES (21)	No	U	To investigate the neuropsychological profile of NEA.	Neuropsychological assessment included a complete examination of intelligence, attention, memory (verbal and visuo-spatial, short term and long term), visuo-construction functions, frontal functions and language comprehension with: Raven's colored progressive matrices, trail making test, attentional matrices, digit span, corsi span, short story test, paired associated words test, Rey–osterrieth complex figure, verbal fluency test (phonemic and semantic) and token test.	There are no statistical significant differences between the three groups regarding the cognitive profile.

van Beilen 2009	Psychological assessment of malingering in psychogenic neurological disorders and non-psychogenic neurological disorders: relationship to psychopathology levels	FMD (26)	Neurological disorders (organic) (ND) (26); HC (18)	"Amsterdamse Korte Termijn Geheugen test" (AKTG) and the "Structured Inventory of Malingered Symptomatology" (SIMS)	U	To investigate performance validity on cognitive testing in patients with functional neurological symptoms.		FMD patients scored significantly higher in SIMS as compared with both control groups individually (ND and HC).<br>This was not true for the AKTG, which lost significance when FMD was compared with ND. Thus, the SIMS differentiated between PMD and ND, but the AKTG did not.<br>The authors observed that although functional patients showed the highest levels of psychological complaints and malingering, but non-functional neurological patients also showed significantly more psychological distress and malingering than HC.<br>Psychological distress was related to the degree of malingering, in both patient groups.
Walterfang 2011	Utility and validity of a brief cognitive assessment tool in patients with epileptic and nonepileptic seizures	NEA (50)	ES (87)	No	C (partially)	The aim of the current study was to test the utility and validity of a cognitive screening tool, the Neuropsychiatry Unit Cognitive Assessment Tool (NUCOG) in patients with NEA and ES.	NUCOG battery comprises five domains: Attention, Memory, Language, Visuo-constructional, and Executive Function. Additionally, neuropsychological assessment also included Wechsler Adult Intelligence Scale, Third Edition (WAIS-III), and Wechsler Memory Scale, Third Edition (WMS-III), Boston Naming Test (BNT) [42], Delis–Kaplan Executive Function	Cognitive performance on NUCOG by seizure diagnostic group: There were no significant differences in cognitive performance assessed with the NUCOG between patients with ES, PNES, ES and PNES, and other NES. The mean NUCOG total scores were within the control range of a healthy population, as were all domain scores.

							System (DKEFS) (verbal function), Trail-Making Test (TMT) (executive function) and Wechsler Test of Adult Reading (WTAR)	
Wilkus 1984	Intensive EEG Monitoring and Psychological Studies of Patients with Pseudoepileptic Seizures	NEA (25)	ES (25) (matched for age and educational levels); normative data	No	B (for objective and sometimes for the diagnosis)	To investigate the neuropsychological profile of NEA.	WAIS for intelligence; Neuropsychological Battery for Epilepsy (including 16 perceptual, motor, and cognitive test measures)	The groups did not differ significantly on any of the 11 subtests of the WAIS or any of 16 measures from the Neuropsychological Battery for Epilepsy.<br>However, performance was abnormal in both groups in about one-half of tests of the Neuropsychological Battery.
Wilkus 1989	Factors Affecting the Outcome of MMPI and Neuropsychological Assessments of Psychogenic and Epileptic Seizure Patients	NEA (25)	Partial ES (25); generalized ES A (25); generalized ES B (25); NEA, Partial ES and generalized ES A were matched for age, gender and education; generalized ES B were less educated	No	B (for the objective)	To investigate neuropsychological deficits and emotional disorders in patients with NEA.	WAIS (VIQ, PIQ FSIQ), Neuropsychological Battery for Epilepsy (Stroop parts I and II-I; WMS logical memory and visual reproduction; Perceptual Exam; Speech erception; Name Writing; Category Test; Tactual Performance Test - total time, memory, localization; Seashore rhythm and tonal memory; Tapping, total; TMT-B; Aphasia screening test; Construct, dyspraxia;	Closely matched groups of patients with solely psychogenic seizures, partial seizures, or generalized seizures did not differ in neuropsychological performance (but performed abnormally for about half of the scores) However, patients with NEA performed much better than a less well-educated, but otherwise matched group of patients with generalized epileptic seizures (50% vs 75% abnormal scores).

							Halstead Imp. Index;	
Williamson 2012	Abuse, Not Financial Incentive, Predicts Non-Credible Cognitive Performance in Patients With Psychogenic Non-Epileptic Seizures	NEA (103)	Uncontrolled	Word memory test	U	To investigate the relationship between failure to SVT and the likelihood of seeking financial compensation, presence of severe psychopathology and a previous history of abuse.		PVT: 58 passed vs 32 failed WMT<br>The NEA group failed the WMT at a rate (35%) similar to that of compensation-seeking populations<br>1. Were those participants who failed WMT more likely to have an obvious financial incentive? Contrary to authors' expectations, patients with financial incentives did not fail WMT at a greater rate than those without them.<br>2. Did those participants failing WMT report greater levels of psychopathology on the MMPI-2? An independent group t-test failed to reveal a significant difference between the groups.<br>3. Are those failing WMT more likely to report physical, emotional, or sexual abuse? Participants failing WMT were more likely to report a history of some form of abuse.
